# Decarboxylative aldol reaction of α,α-difluoro-β-ketocarboxylate salt: a facile method for generation of difluoroenolate†

**DOI:** 10.1039/c8ra02440e

**Published:** 2018-06-05

**Authors:** Atsushi Tarui, Mayuna Oduti, Susumu Shinya, Kazuyuki Sato, Masaaki Omote

**Affiliations:** Faculty of Pharmaceutical Sciences, Setsunan University 45-1 Nagaotogecho Hirakata Osaka 573-0101 Japan Omote@pharm.setsunan.ac.jp

## Abstract

We developed a decarboxylative aldol reaction using α,α-difluoro-β-ketocarboxylate salt, carbonyl compounds, and ZnCl_2_/*N*,*N*,*N*′,*N*′-tetramethylethylenediamine. The generation of difluoroenolate proceeded smoothly under mild heating to provide α,α-difluoro-β-hydroxy ketones in good to excellent yield (up to 99%). The α,α-difluoro-β-ketocarboxylate salt was bench stable and easy to handle under air, which realizes a convenient and environmentally friendly methodology for synthesis of difluoromethylene compounds.

## Abstract 2

A ZnCl_2_/*N*,*N*,*N*′,*N*′-tetramethylethylenediamine complex promoted decarboxylative aldol reaction of α,α-difluoro-β-ketocarboxylate salt with carbonyl compounds has been developed.

## Graphical abstract

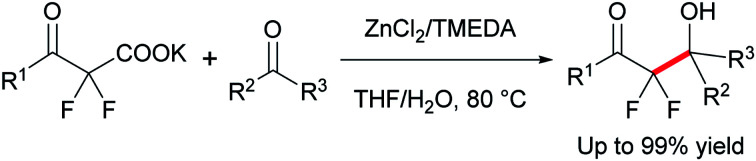

## Introduction

**Fig. 1:**
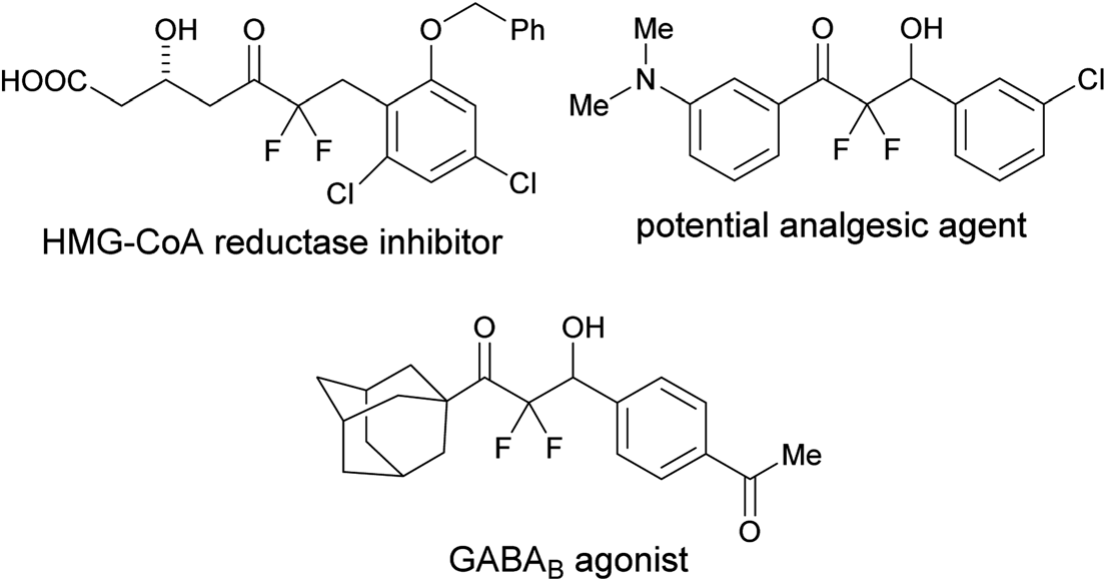
Bioactive α,α-difluoroketones.

Organic molecules containing a difluoromethylene group are particularly useful in medicinal chemistry.^1^ Among these, α,α-difluoroketones show a variety of bioactivities such as cholesterol-lowering, analgesic, and GABA_B_ agonist activities (Fig. 1).^2^ Thus, simple and mild strategies for accessing α,α-difluoroketone substructures should be useful for developing new therapeutic candidates.

**Scheme 1:**
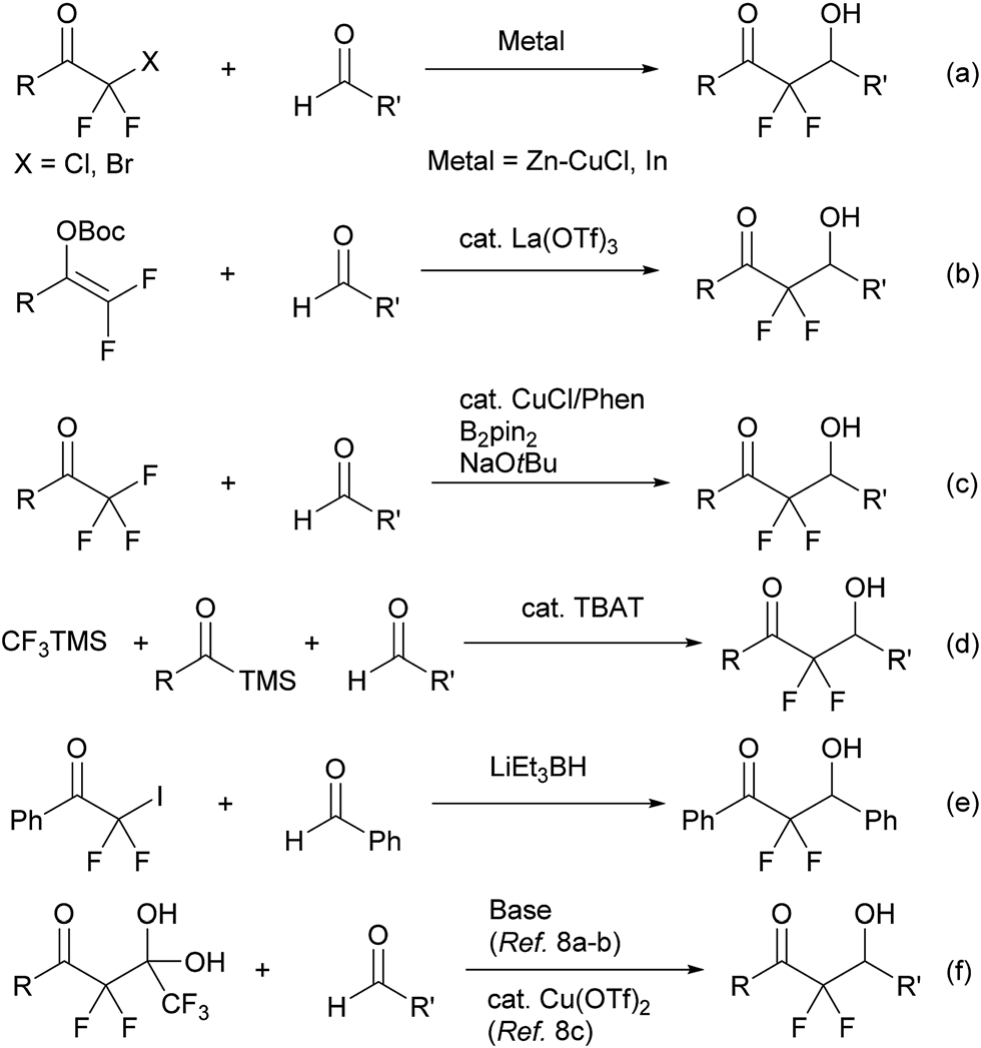
Various methods for a generation of α,α-difluoroenolate.

α,α-Difluoroenolate plays a key role in the construction of CF_2_–carbon bonds and many synthetic routes have been reported based on this nucleophilic synthon, including a metal-mediated Reformatsky reaction of halodifluoromethyl ketone (Scheme 1, eqn (a)),^3^ a Lewis acid-catalyzed aldol reaction of difluoroenol *O*-Boc esters (Scheme 1, eqn (b)),^4^ a copper-catalyzed reaction of α,α,α-trifluoromethylketones *via* β-fluoro elimination (Scheme 1, eqn (c)),^5^ a one-pot reaction of acylsilanes and trifluoromethyltri-methylsilane (TMSCF_3_) with aldehydes (Scheme 1, eqn (d)),^6^ an aldol reaction of halodifluoromethyl ketone *via* reduction of halogen process by lithium triethylborohydride (Scheme 1, eqn (e)),^7^ and a detrifluoroacetylative aldol reaction of trifluoromethyl α,α-difluoro-β-keto *gem*-diols (Scheme 1, eqn (f)).^8^ In the course of these studies, decarboxylation of β-keto acids is a mild and convenient method for providing the corresponding enolate and is an environmentally friendly system.^9^ Wennemers has reported the first decarboxylative process for the preparation of fluorinated enolate using fluoromalonic acid halfthioesters (F-MAHT).^10^ Recently, two groups have reported the synthesis of α,α-difluoro-β-hydroxy ketones using 2,2-difluoro-3-oxo-3-phenyl-propanoic acid (1). The first route was based on an aldol reaction of 1 with aldehydes under metal-free conditions, from the Mao group.^11^ The other route involved copper-catalyzed difluoroalkylation of aromatic aldehydes using 1 from the Mai group.^12^ However, the former reaction needed relative long reaction times and a high reaction temperature (100 °C). In the latter reaction, the scope of aldehydes was limited and only aromatic aldehydes were suitable for the transformation. In this paper, the simple potassium 2,2-difluoro-3-oxo-3-phenylpro-panoate (2a) was used as a precursor of difluoroenolate for the decarboxylative aldol reaction with carbonyl compounds under mild reaction conditions.

## Results and discussion

**Scheme 2:**
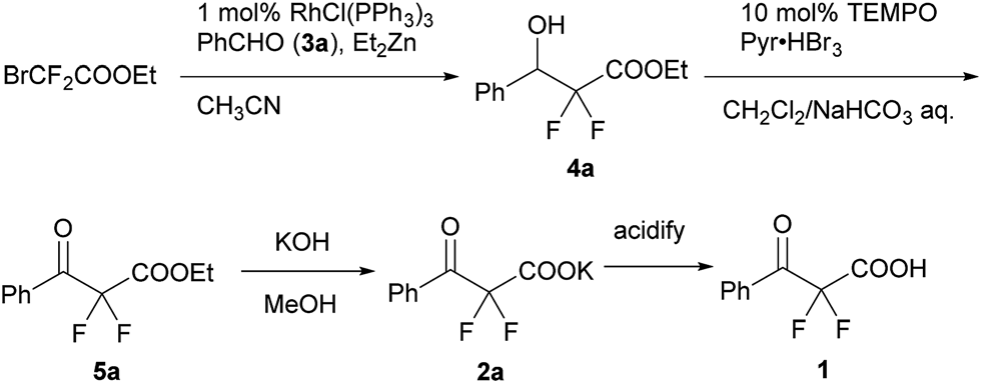
The synthesis of a potassium α,α-difluoro-β-keto carboxylate (2a) and its carboxylic acid (1).

We used a potassium salt of α,α-difluoro-β-keto acid 1 (2a) as a model precursor of difluoroenolate to examine the decarboxylative aldol reaction with benzaldehyde (3a). The model substrate 2a was synthesized as following 3 steps (Scheme 2);^13^ Honda–Reformatsky reaction of ethyl bromodifluoroacetate with 3a gave β-hydoroxy-α,α-difluoroacetate 4a, then 4a was oxidized to β-keto-α,α-difluoroester 5a by TEMPO oxidation. The saponification of β-ketoester 5a gave rise to the potassium carboxylate 2a. The substrate 2a was isolated easily by filtration from the reaction mixture as a non-hygroscopic compound which enabled easy handling even under air atmosphere. Another substrate 1 was obtained by the acidified of 2a and was used to the desired reaction without purification. The results for the decarboxylative aldol reaction of 2a and 1 are summarized in Table 1. To examine decarboxylation under mild heating (50 °C) efficiently, ZnCl_2_ was used as an acceptor of the enolate (Table 1, entry 1). The reaction of 2a provided the desired product in low yield (20% yield). In the case of using carboxylic acid 1, only trace amounts of the desired product 6aa were obtained (entry 2). To improve the yield of 6aa, the reaction temperature was elevated to 80 °C and a moderate yield of product was obtained (60%, entry 3). The current screening of the reaction conditions was performed in dry THF (entries 1–3). Using wet THF in this system, we observed a considerable improvement of the yield of 6aa. Thus, when an equimolar amount of H_2_O for 2a was added to dry THF, the desired product was obtained in 84% yield reproducibly (entry 4). It was important to use ZnCl_2_ in the current system and a lower yield of 6aa was observed in the absence of ZnCl_2_ (entry 5). To evaluate the effects of the metal, other Lewis acids, including boron and ytterbium, were examined (entries 6–8). Zinc metal was shown to be the most effective and the bench stable reagent ZnCl_2_*N*,*N*,*Nʹ*,*Nʹ*-tetramethylethylenediamine complex (ZnCl_2_/TMEDA) was the best metal source in this decarboxylative aldol process (Entry 8). ZnCl_2_/TMEDA was commercially available and was easy to handle compared with hygroscopic ZnCl_2_.^14^ Further optimization of the reaction conditions showed that the 1.2 equivalent of 2a and ZnCl_2_/TMEDA provided a 98% yield of 6aa (entry 9).^15^ Among the solvents tested, THF was the best solvent for this reaction.^16^ Furthermore, we examined the effects of the addition of water using dry THF solvent. Under the anhydrous conditions, a lower yield of product was observed (entry 10). When a catalytic amount of H_2_O (10 mol%) was added, there was no effect on the yield of product and benzaldehyde was recovered from the reaction mixture after 5 h (entry 11). Other proton sources such as ethanol and 2,2,2-trifluoroethanol were added to the reaction media. However, these additives did not markedly affect the yield of 6aa. In these cases (entries 10–13), the decarboxylation of 2a did not occur effectively and α,α-difluoro-β-keto acid (1) was detected in ^19^F NMR of the crude reaction mixture.

**Table 1:** Screening reaction conditions

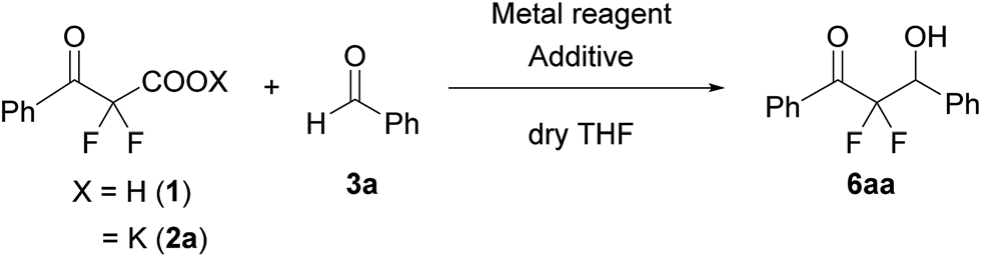									
Entry	Metal reagent	(Equiv.)	Substrates	(Equiv.)	Additive	(Equiv.)	Temp. (°C)	Time (h)	Yield of 6aa (%)^a^
1	ZnCl_2_	(1.0)	2a	(1.0)	None		50	24	20
2	ZnCl_2_	(1.0)	1	(1.0)	None		50	24	Trace
3	ZnCl_2_	(1.0)	2a	(1.0)	None		80	8	60
4	ZnCl_2_	(1.0)	2a	(1.0)	H_2_O	(1.0)	80	8	84
5	None		2a	(1.0)	H_2_O	(1.0)	80	5	46
6	BF_3_–Et_2_O	(1.0)	2a	(1.0)	H_2_O	(1.0)	80	16	59
7	Yb(OTf)_3_	(0.1)	2a	(1.0)	H_2_O	(1.0)	80	26	32
8	ZnCl_2_–TMEDA	(1.0)	2a	(1.0)	H_2_O	(1.0)	80	5	88
9	ZnCl_2_–TMEDA	(1.2)	2a	(1.2)	H_2_O	(1.0)	80	5	98
10	ZnCl_2_–TMEDA	(1.2)	2a	(1.2)	None		80	5	42^b^
11	ZnCl_2_–TMEDA	(1.2)	2a	(1.2)	H_2_O	(0.1)	80	5	50^b^
12	ZnCl_2_–TMEDA	(1.2)	2a	(1.2)	EtOH	(1.0)	80	7	59^b^
13	ZnCl_2_–TMEDA	(1.2)	2a	(1.2)	CF_2_CH_2_OH	(1.0)	80	7	58^b^

a Isolated yield.

b ^19^F NMR yields.

After optimization of the reaction conditions for this decarboxylative aldol reaction, various substrates were tested (Table 2). Various aldehydes 3 reacted with 2,2-difluoro-3-oxo-3-phenylpropanoate (2a) to provide the desired products in good to excellent yields. Aromatic aldehydes were especially suitable for the reaction (6ab–6al). The electroproperties and positions of the substituents on the phenyl ring of the aldehydes did not affect the yield of the reaction. Among these, the reaction of functionalized aldehydes, such as methoxycarbonyl and cyano groups, also provided the corresponding products (6ag and 6ah) in good yields. However, in the case of 2-pyridinecarboxaldehyde, only 39% of product (6am) was obtained. Furthermore, enolizable aliphatic aldehydes were also tolerated, providing the corresponding aldol products 6an and 6ao in good yields. The scope of the 2,2-difluoro-3-oxo-propanoates (2b–d) was also tested.^17^ Both electron-donating (CH_3_O) and electron-withdrawing groups (Cl) on the phenyl ring of the substrate 2 were well tolerated in the decarboxylative aldol process. Furthermore, the aliphatic substrate 2d produced the corresponding product 6da in good yield. For the reaction with ketones, when an excess amount of the ketones was used, the desired products 6ap and 6aq were obtained in moderate yields of 60% and 53%, respectively.

**Table 2:** Decarboxylative aldol reaction of potassium 2,2-difluoro-3-oxopropanoates 2 with carbonyl compounds

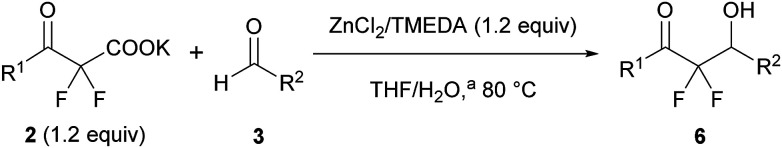		
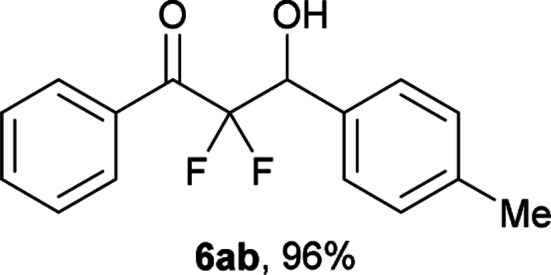	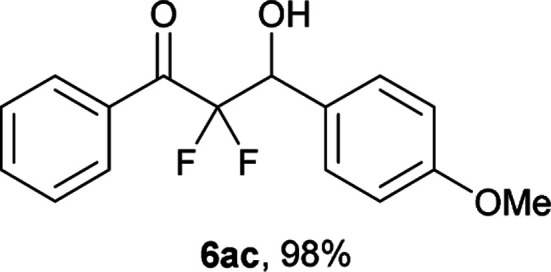	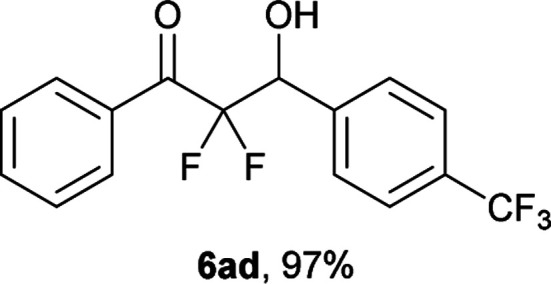
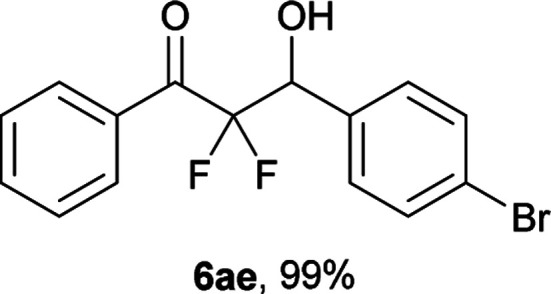	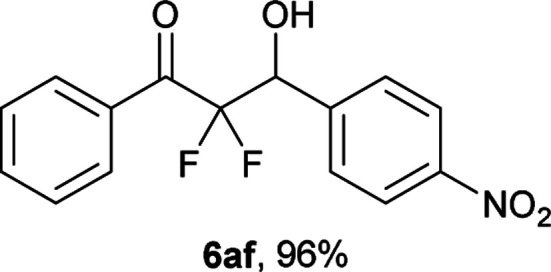	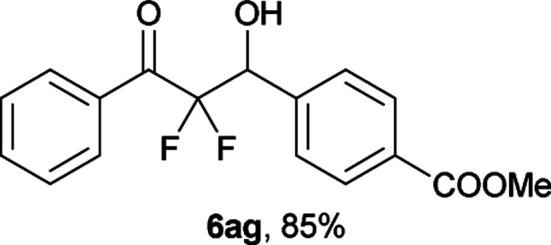
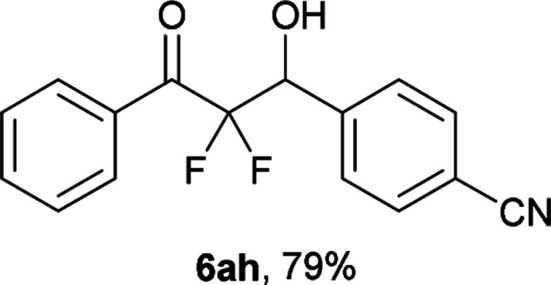	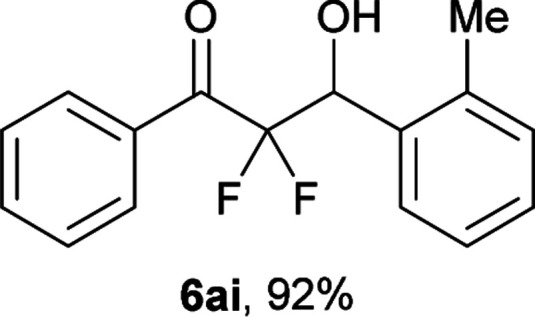	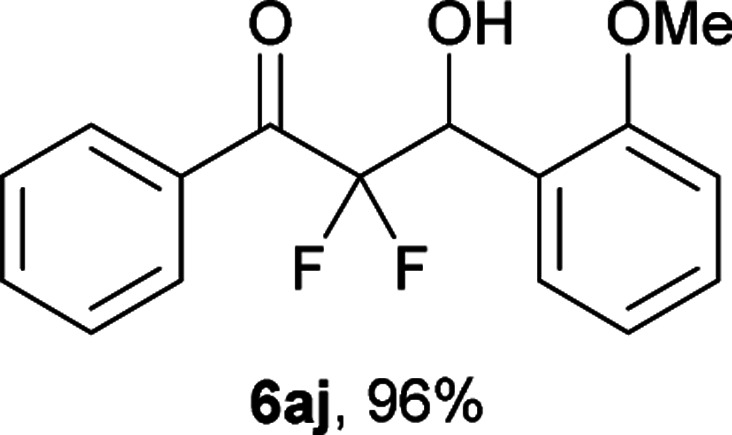
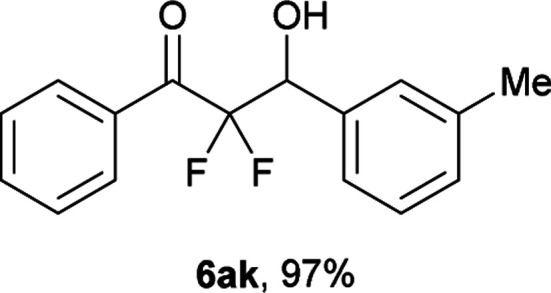	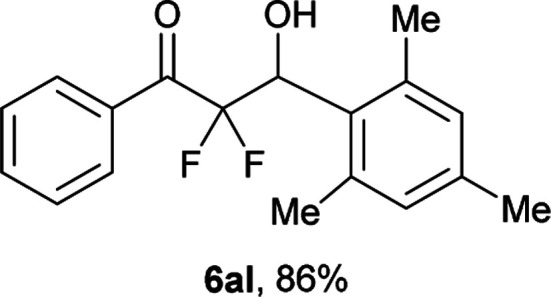	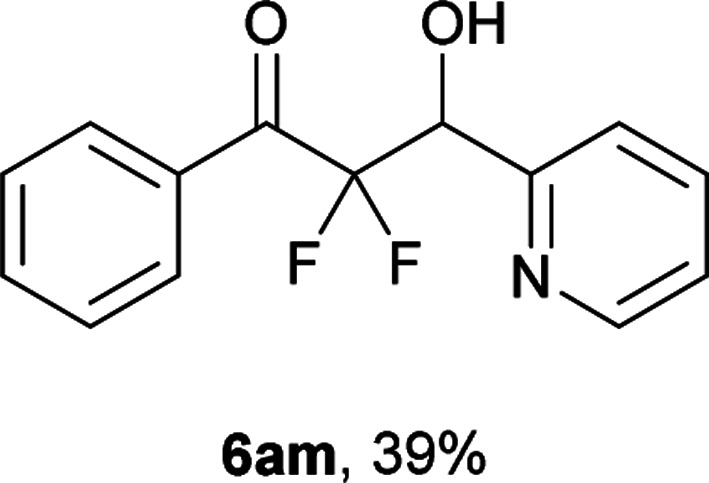
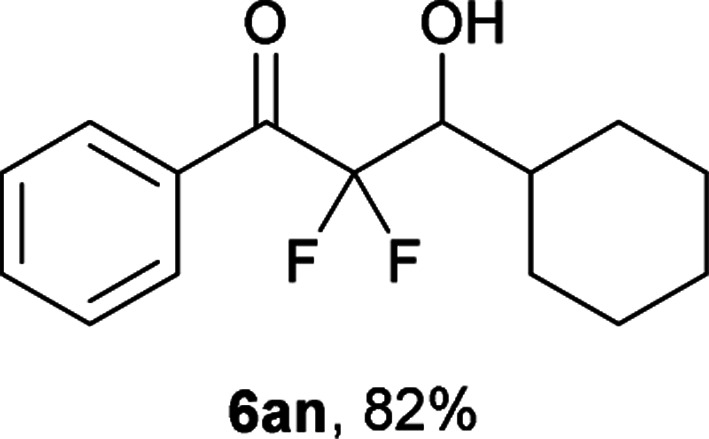	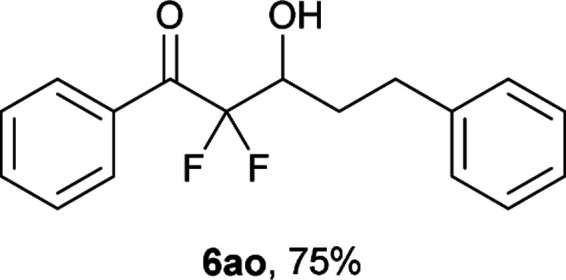	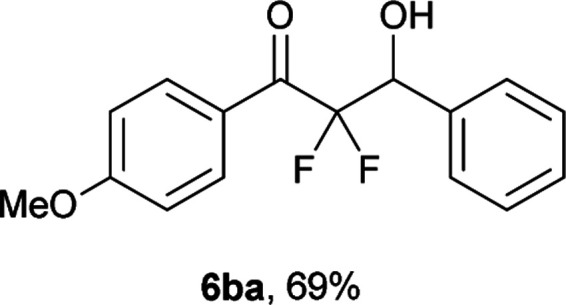
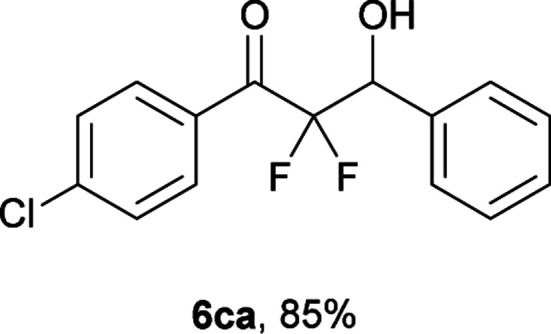	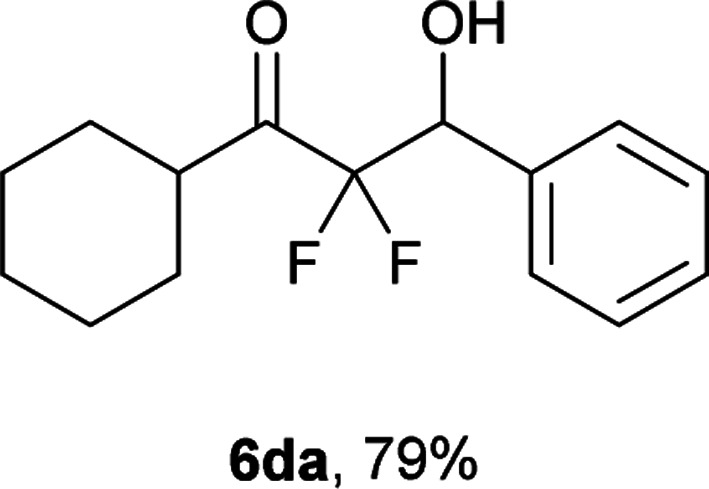	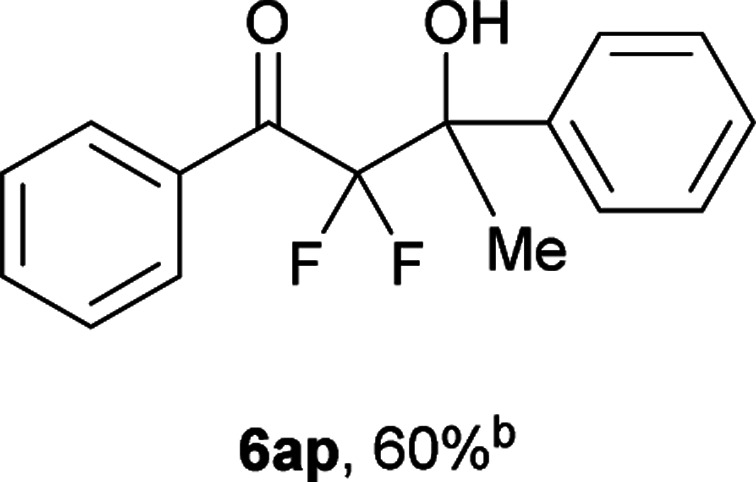
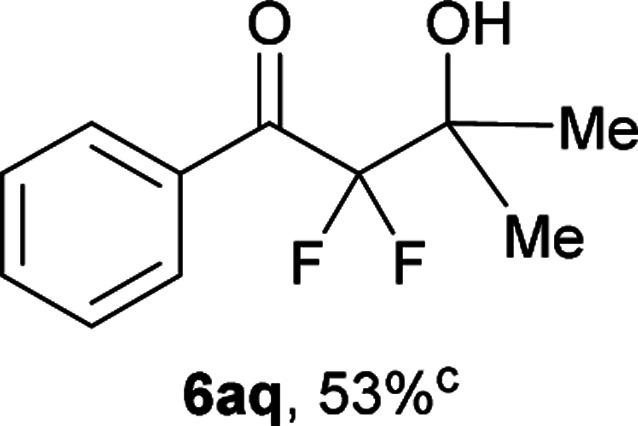		

a 1.0 Equivalents of H_2_O was added.

b 3 Equivalents of acetophenone was used.

c Excess amount of acetone (1 mL) was used.

**Scheme 3:**
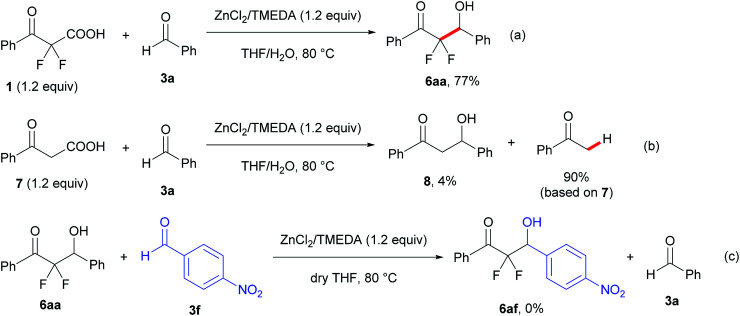
Control experiments and examination of retro-aldol reaction.

To examine the reaction mechanism, a control experiment was conducted with 2,2-difluoro-3-oxo-3-phenylpropanoic acid (1) and a non-fluorinated 3-oxo-3-phenylpropanoic acid (7) under the optimized reaction conditions (Scheme 2, eqn (a) and (b)). The reaction of compound 1 with 3a showed a decrease in the yield of product and a prolonged reaction time, which provided the aldol product 6aa in 77% yield for 19 h under the optimal conditions. However, trace amounts of the aldol product 8 were obtained from a non-fluorinated substrate 7 along with a high yield of acetophenone (90% based on 7) *via* a decarboxylative process. In reports on palladium-catalyzed benzylation reactions of α,α-difluoroketone enolate, Altman suggested that rehybridization of the α,α-difluorinated enolate carbanions from C (sp3) to C (sp2) actually occurs more slowly than for non-fluorinated enolates.^17^ This report and our results suggest that the α,α-difluorinated enolate from 2a prefers C–enolate form. As a result, the reaction of C–enolate together with the high nucleophilicity led to the aldol product more effectively than a non-fluorinated enolate generated from 7. Moreover, when a scrambling experiment of the aldol product 6aa with another aromatic aldehyde 3f was performed under the current reaction conditions (Scheme 3, eqn (c)), no retro-aldol reaction was observed and the scrambling product 6af was not formed.

On the basis of previous works on the synthesis of α,α-difluoro-β-hydroxyketones and our experiments,^11,12^ a tentative reaction mechanism for this decarboxylative aldol reaction of potassium α,α-difluoro-β-ketocarboxylate (2a) with benzaldehyde (3a) is proposed. First, zinc(ii) is accepted by the nucleophilic enolate generated from decarboxylation of 2a. Then, the nucleophilic addition of zinc difluoroenolate to 3a occurred to lead to the formation of aldol alkoxide. Stoichiometric amount of water promotes the protonation of zinc alkoxide for the formation of the product 6aa in the equilibrium of the aldol process.

## Conclusions

In conclusion, we have successfully developed a mild decarboxylative aldol reaction for potassium α,α-difluoro-β-keto carboxylate with aldehydes. The reaction is mild, with a reaction temperature below 100 °C, and a variety of α,α-difluoro-β-hydroxy ketones with biological activity could be obtained in good to excellent yields. Compared with previous methods, the substrates 2a–d used in this reaction are bench stable salts and a broad substrate scope was realized. Now we are investigating an asymmetric version of this decarboxylative aldol reaction.

## Experimental

### General

NMR spectra were obtained from a solution in CDCl_3_ using 400 MHz for ^1^H, 100 MHz for ^13^C, 376 MHz for ^19^F. Chemical shifts of ^1^H and ^13^C NMR are reported in ppm from tetramethylsilane (TMS) as an internal standard. Chemical shifts of ^19^F NMR are reported in ppm from CFCl_3_ as an internal standard. All data are reported as follows: chemical shifts, multiplicity (s = singlet, bs = broad singlet, d = doublet, t = triplet, q = quartet, dd = double doublet, ddd = double double doublet, m = multiplet), coupling constants (Hz), and relative integration value. HRMS experiments were measured on a double-focusing mass spectrometer with an ionization mode of EI. Melting points were measured uncorrected.

All experiments were carried out under argon atmosphere in flame-dried glassware using standard inert techniques for introducing reagents and solvents unless otherwise noted. Tetrahydrofuran (THF) was purchased from Kanto Chemical Co. Inc. as “Dehydrated”. All commercially available materials were used as received without further purification.

### General procedure for the decarboxylative aldol reaction

To a dry and argon-flushed reaction vessel, equipped with a magnetic stirrer, were added zinc chloride *N*,*N*,*N*′,*N*′-tetramethylethylenediamine complex (151 mg, 0.6 mmol) and potassium 2,2-difluoro-3-oxopropanoate (2, 0.6 mmol). The solids were suspended in THF (4 mL), then the corresponding aldehyde (0.5 mmol) and THF solution of H_2_O (0.5 mL, 1 M) were added. The reaction mixture was heated at 80 °C with stirring for 5 h. The reaction was quenched with 10% aqueous HCl, and the resultant mixture was extract with AcOEt. The combined organic phases were washed with brine and dried over MgSO_4_. Then the extract was concentrated *in vacuo*, and the residue was purified by column chromatography on silica gel to give the corresponding aldol adducts 6.

#### 2,2-Difluoro-3-hydroxy-1,3-diphenylpropan-1-one (6aa)^8*c*^

The titled product (6aa) was obtained as a colorless liquid in 98% yield (128.1 mg), after column chromatography on silica gel (AcOEt/hexane = 1 : 4). ^1^H NMR (400 MHz, CDCl_3_) *δ* 8.06–8.04 (m, 2H), 7.65–7.61 (m, 1H), 7.50–7.45 (m, 4H), 7.41–7.38 (m, 3H), 5.38 (ddd, *J* = 18.7, 5.6, 4.6 Hz, 1H), 3.00 (d, *J* = 4.6 Hz, 1H); ^13^C NMR (CDCl_3_, 100 MHz) *δ* 190.9 (dd, *J* = 31.3, 29.2 Hz), 134.7, 134.6, 132.4 (m), 130.2 (m), 129.0, 128.6, 128.3, 128.1, 115.7 (dd, *J* = 261.5, 256.8 Hz), 73.3 (dd, *J* = 29.2, 23.1 Hz); ^19^F NMR (376 MHz, CDCl_3_) *δ* −104.7 (dd, *J*_FF_ = 293, *J*_HF_ = 5.6 Hz, 1F), −116.3 (dd, *J*_FF_ = 293, *J*_HF_ = 18.7 Hz, 1F); HRMS (EI) *m*/*z* calcd for C_15_H_12_F_2_O_2_ [M]^+^ 262.0805, found 262.0799.

#### 2,2-Difluoro-3-hydroxy-1-phenyl-3-(*p*-tolyl)propan-1-one (6ab)^8*c*^

The titled product (6ab) was obtained as a colorless liquid in 96% yield (132.9 mg), after column chromatography on silica gel (AcOEt/hexane = 1 : 9). ^1^H NMR (400 MHz, CDCl_3_) *δ* 8.06–8.04 (m, 2H), 7.65–7.61 (m, 1H), 7.49–7.45 (m, 2H), 7.38 (d, *J* = 8.0 Hz, 2H), 7.20 (d, *J* = 8.0 Hz, 2H), 5.34 (ddd, *J* = 18.6, 5.6, 3.6 Hz, 1H), 2.95 (m, 1H), 2.36 (s, 3H); ^13^C NMR (CDCl_3_, 100 MHz) *δ* 190.9 (dd, *J* = 31.3, 29.5 Hz), 138.9, 134.5, 132.5, 131.7, 130.2 (m), 129.0, 128.6, 128.0, 115.8 (dd, *J* = 261.2, 256.1 Hz), 73.2 (dd, *J* = 28.2, 23.3 Hz), 21.2; ^19^F NMR (376 MHz, CDCl_3_) *δ* −104.8 (dd, *J*_FF_ = 291, *J*_HF_ = 5.6 Hz, 1F), −116.4 (dd, *J*_FF_ = 291, *J*_HF_ = 18.6 Hz, 1F); HRMS (EI) *m*/*z* calcd for C_16_H_14_F_2_O_2_ [M]^+^ 276.0962, found 276.0966.

#### 2,2-Difluoro-3-hydroxy-3-(4-methoxyphenyl)-1-phenylpropan-1-one (6ac)^5^

The titled product (6ac) was obtained as a colorless liquid in 98% yield (143.4 mg), after column chromatography on silica gel (AcOEt/hexane = 1 : 9). ^1^H NMR (400 MHz, CDCl_3_) *δ* 8.06–8.04 (m, 2H), 7.65–7.61 (m, 1H), 7.49–7.45 (m, 2H), 7.42 (d, *J* = 8.4 Hz, 2H), 6.92 (d, *J* = 8.4 Hz, 2H), 5.32 (ddd, *J* = 18.5, 5.9, 3.8 Hz, 1H), 3.82 (s, 3H), 2.98 (m, 1H); ^13^C NMR (CDCl_3_, 100 MHz) *δ* 191.0 (dd, *J* = 30.6, 28.5 Hz), 160.1, 134.5, 132.5, 130.2 (m), 129.4, 128.6, 126.8, 115.8 (dd, *J* = 263.9, 256.3 Hz), 113.8, 72.9 (dd, *J* = 27.8, 23.1 Hz), 55.3; ^19^F NMR (376 MHz, CDCl_3_) *δ* −105.1 (dd, *J*_FF_ = 290, *J*_HF_ = 5.9 Hz, 1F), −116.4 (dd, *J*_FF_ = 290, *J*_HF_ = 18.5 Hz, 1F); HRMS (EI) *m*/*z* calcd for C_16_H_14_F_2_O_3_ [M]^+^ 292.0911, found 292.0912.

#### 2,2-Difluoro-3-hydroxy-1-phenyl-3-(4-(trifluoromethyl)phenyl)-propan-1-one (6ad)^18^

The titled product (6ad) was obtained as a colorless solid in 97% yield (160.2 mg), after column chromatography on silica gel (AcOEt/hexane = 1 : 9). mp 104.0–104.5 °C (from Et_2_O–C_6_, lit. 87–89 °C); ^1^H NMR (400 MHz, CDCl_3_) *δ* 8.08–8.06 (m, 2H), 7.68–7.63 (m, 5H), 7.52–7.48 (m, 2H), 5.46 (ddd, *J* = 19.1, 5.2, 4.5 Hz, 1H), 3.23 (d, *J* = 4.5 Hz, 1H); ^13^C NMR (CDCl_3_, 100 MHz) *δ* 191.5 (dd, *J* = 31.6, 30.0 Hz), 138.5, 134.9, 130.0 (m), 131.1 (q, *J* = 32.5 Hz), 130.3 (m), 128.8, 128.6, 125.1 (m), 123.9 (q, *J* = 271.9 Hz), 115.3 (dd, *J* = 273.9, 257.4 Hz), 72.6 (dd, *J* = 28.4, 23.0 Hz); ^19^F NMR (376 MHz, CDCl_3_) *δ* −62.6 (s, 3F), −103.8 (dd, *J*_FF_ = 299, *J*_HF_ = 5.2 Hz, 1F), −116.5 (dd, *J*_FF_ = 299, *J*_HF_ = 19.1 Hz, 1F); HRMS (EI) *m*/*z* calcd for C_16_H_11_F_5_O_2_ [M]^+^ 330.0679, found 330.0688.

#### 3-(4-Bromophenyl)-2,2-difluoro-3-hydroxy-1-phenylpropan-1-one (6ae)^3*c*,5^

The titled product (6ae) was obtained as a colorless solid in 99% yield (169.6 mg), after column chromatography on silica gel (AcOEt/hexane = 1 : 4). mp 103.0–103.5 °C (from Et_2_O–C_6_, 96–97 °C); ^1^H NMR (400 MHz, CDCl_3_) *δ* 8.07–8.05 (m, 2H), 7.67–7.63 (m, 1H), 7.54–7.47 (m, 4H), 7.39–7.37 (m, 2H), 5.35 (ddd, *J* = 18.8, 5.3, 4.5 Hz, 1H), 3.08 (d, *J* = 4.5 Hz, 1H); ^13^C NMR (CDCl_3_, 100 MHz) *δ* 190.6 (dd, *J* = 30.6, 29.0 Hz), 134.8, 133.6, 132.0 (m), 131.4, 130.3 (m), 129.8, 128.8, 123.2, 115.3 (dd, *J* = 269.1, 257.2 Hz), 72.6 (dd, *J* = 28.0, 22.5 Hz); ^19^F NMR (376 MHz, CDCl_3_) *δ* −104.2 (dd, *J*_FF_ = 298, *J*_HF_ = 5.3 Hz, 1F), −116.6 (dd, *J*_FF_ = 298, *J*_HF_ = 18.8 Hz, 1F); HRMS (EI) *m*/*z* calcd for C_15_H_11_BrF_2_O_2_ [M]^+^ 339.9910, found 339.9912 (16.9), 341.9895(16.8).

#### 2,2-Difluoro-3-hydroxy-3-(4-nitrophenyl)-1-phenylpropan-1-one (6af)^18^

The titled product (6af) was obtained as a colorless solid in 96% yield (147.5 mg), after column chromatography on silica gel (AcOEt/hexane = 3 : 7). mp 114.0–115.0 °C (from Et_2_O–C_6_); ^1^H NMR (400 MHz, CDCl_3_) *δ* 8.26 (d, *J* = 8.6 Hz, 2H), 8.09–8.08 (m, 2H), 7.72–7.65 (m, 3H), 7.53–7.49 (m, 2H), 5.53 (ddd, *J* = 19.3, 4.5, 4.1 Hz, 1H), 3.35 (d, *J* = 4.5 Hz, 1H); ^13^C NMR (CDCl_3_, 100 MHz) *δ* 190.2 (dd, *J* = 31.6, 29.6 Hz), 148.3, 141.6, 135.1, 131.7 (m), 130.3 (m), 129.2, 128.8, 123.3, 115.0 (dd, *J* = 268.2, 257.2 Hz), 72.2 (dd, *J* = 28.8, 22.5 Hz); ^19^F NMR (376 MHz, CDCl_3_) *δ* −103.3 (dd, *J*_FF_ = 301, *J*_HF_ = 4.1 Hz, 1F), −116.5 (dd, *J*_FF_ = 301, *J*_HF_ = 19.3 Hz, 1F); HRMS (EI) *m*/*z* calcd for C_15_H_11_F_2_NO_4_ [M]^+^ 307.0656, found 307.0655.

#### Methyl 4-(2,2-difluoro-1-hydroxy-3-oxo-3-phenylpropyl)benzo-ate (6ag)^8*c*^

The titled product (6ag) was obtained as a colorless solid in 85% yield (136.3 mg), after column chromatography on silica gel (AcOEt/hexane = 1 : 4). mp 100.5–101.5 °C (from Et_2_O–C_6_); ^1^H NMR (400 MHz, CDCl_3_) *δ* 8.07 (m, 4H), 7.67–7.64 (m, 1H), 7.59 (d, *J* = 8.4, 2H), 7.51–7.47 (m, 2H), 5.46 (ddd, *J* = 19.0, 4.6, 4.6 Hz, 1H), 3.93 (s, 3H), 3.17 (d, *J* = 4.6 Hz, 1H); ^13^C NMR (CDCl_3_, 100 MHz) *δ* 190.6 (dd, *J* = 32.0, 29.6 Hz), 166.8, 139.5, 134.8, 132.0 (m), 130.7, 130.3 (m), 129.5, 128.8, 128.2, 115.3 (dd, *J* = 261.8, 257.4 Hz), 72.8 (dd, *J* = 28.3, 23.0 Hz), 52.2; ^19^F NMR (376 MHz, CDCl_3_) *δ* −103.9 (dd, *J*_FF_ = 297, *J*_HF_ = 4.6 Hz, 1F), −116.4 (dd, *J*_FF_ = 297, *J*_HF_ = 19.0 Hz, 1F); HRMS (EI) *m*/*z* calcd for C_17_H_14_F_2_O_4_ [M]^+^ 320.0860, found 320.0864.

#### 4-(2,2-Difluoro-1-hydroxy-3-oxo-3-phenylpropyl)benzonitrile (6ah)^18^

The titled product (6ah) was obtained as a colorless solid in 79% yield (113.0 mg), after column chromatography on silica gel (AcOEt/hexane = 1 : 9). mp 94.0–95.5 °C (from CHCl_3_–C_6_, lit. 79–81 °C); ^1^H NMR (400 MHz, CDCl_3_) *δ* 8.08–8.07 (m, 2H), 7.71–7.63 (m, 5H), 7.52–7.48 (m, 2H), 5.46 (ddd, *J* = 19.1, 4.3, 4.1 Hz, 1H), 3.31 (d, *J* = 4.3 Hz, 1H); ^13^C NMR (CDCl_3_, 100 MHz) *δ* 190.3 (dd, *J* = 31.4, 29.7 Hz), 139.8, 135.0, 131.9, 131.8 (m), 130.3 (m), 128.9, 128.8, 118.5, 115.1 (dd, *J* = 269.7, 258.1 Hz), 112.8, 72.4 (dd, *J* = 28.6, 23.0 Hz); ^19^F NMR (376 MHz, CDCl_3_) *δ* −103.5 (dd, *J*_FF_ = 301, *J*_HF_ = 4.1 Hz, 1F), −116.5 (dd, *J*_FF_ = 301, *J*_HF_ = 19.1 Hz, 1F); HRMS (EI) *m*/*z* calcd for C_16_H_11_F_2_NO_2_ [M]^+^ 287.0758, found 287.0760.

#### 2,2-Difluoro-3-hydroxy-1-phenyl-3-(*o*-tolyl)propan-1-one (6ai)^8*c*^

The titled product (6ai) was obtained as a colorless liquid in 92% yield (126.9 mg), after column chromatography on silica gel (AcOEt/hexane = 1 : 9). ^1^H NMR (400 MHz, CDCl_3_) *δ* 8.09–8.07 (m, 2H), 7.67–7.62 (m, 2H), 7.50–7.46 (m, 2H), 7.29–7.19 (m, 3H), 5.70 (ddd, *J* = 20.3, 4.4, 3.8 Hz, 1H), 2.91 (d, *J* = 4.4 Hz, 1H), 2.39 (s, 3H); ^13^C NMR (CDCl_3_, 100 MHz) *δ* 191.0 (dd, *J* = 31.9, 29.4 Hz), 136.8, 134.6, 133.3, 132.4 (m), 130.4, 130.3 (m), 128.8, 128.7, 128.1, 126.1, 116.4 (dd, *J* = 257.9, 255.5 Hz), 69.0 (dd, *J* = 29.2, 22.6 Hz), 19.6; ^19^F NMR (376 MHz, CDCl_3_) *δ* −104.1 (dd, *J*_FF_ = 294, *J*_HF_ = 3.8 Hz, 1F), −116.9 (dd, *J*_FF_ = 294, *J*_HF_ = 20.3 Hz, 1F); HRMS (EI) *m*/*z* calcd for C_16_H_14_F_2_O_2_ [M]^+^ 276.0962, found 276.0967.

#### 2,2-Difluoro-3-hydroxy-3-(2-methoxyphenyl)-1-phenylpropan-1-one (6aj)^4^

The titled product (6aj) was obtained as a colorless liquid in 96% yield (139.0 mg), after column chromatography on silica gel (AcOEt/hexane = 1 : 4). ^1^H NMR (400 MHz, CDCl_3_) *δ* 8.05–8.03 (m, 2H), 7.63–7.59 (m, 1H), 7.49–7.44 (m, 3H), 7.36–7.32 (m, 1H), 7.05–7.01 (m, 1H), 6.87–6.85 (m, 1H), 5.66 (ddd, *J* = 17.3, 7.3, 7.1 Hz, 1H), 3.69 (s, 3H), 3.58 (d, *J* = 7.3 Hz, 1H); ^13^C NMR (CDCl_3_, 100 MHz) *δ* 190.1 (dd, *J* = 30.0, 29.1 Hz), 157.4, 134.2, 132.9, 130.2, 130.1 (m), 129.6, 128.5, 122.8, 120.9, 116.8 (dd, *J* = 264.0, 258.2 Hz), 110.9, 70.2 (dd, *J* = 28.0, 24.4 Hz), 55.3; ^19^F NMR (376 MHz, CDCl_3_) *δ* −106.8 (dd, *J*_FF_ = 275, *J*_HF_ = 7.1 Hz, 1F), −114.7 (dd, *J*_FF_ = 275, *J*_HF_ = 17.3 Hz, 1F); HRMS (EI) *m*/*z* calcd for C_16_H_14_F_2_O_3_ [M]^+^ 292.0911, found 292.0912.

#### 2,2-Difluoro-3-hydroxy-1-phenyl-3-(*m*-tolyl)propan-1-one (6ak)^18^

The titled product (6ak) was obtained as a colorless liquid in 97% yield (133.7 mg), after column chromatography on silica gel (AcOEt/hexane = 1 : 9). ^1^H NMR (400 MHz, CDCl_3_) *δ* 8.06–8.04 (m, 2H), 7.65–7.61 (m, 1H), 7.49–7.45 (m, 2H), 7.31–7.17 (m, 4H), 5.33 (ddd, *J* = 18.7, 5.4, 4.3 Hz, 1H), 2.96 (d, *J* = 4.3 Hz, 1H), 2.37 (s, 3H); ^13^C NMR (CDCl_3_, 100 MHz) *δ* 191.0 (dd, *J* = 31.0, 29.3 Hz), 138.0, 134.6, 134.5, 132.5 (m), 130.2 (m), 129.8, 128.7, 128.6, 128.2, 125.2, 115.8 (dd, *J* = 265.9, 256.8 Hz), 73.3 (dd, *J* = 28.7, 23.1 Hz), 21.4; ^19^F NMR (376 MHz, CDCl_3_) *δ* −104.6 (dd, *J*_FF_ = 291, *J*_HF_ = 5.4 Hz, 1F), −116.4 (dd, *J*_FF_ = 291, *J*_HF_ = 18.7 Hz, 1F); HRMS (EI) *m*/*z* calcd for C_16_H_14_F_2_O_2_ [M]^+^ 276.0962, found 276.0965.

#### 2,2-Difluoro-3-hydroxy-3-(2,4,6-trimethylphenyl)-1-phenylpro-pan-1-one (6al)^5^

The titled product (6al) was obtained as a colorless liquid in 86% yield (130.9 mg), after column chromatography on silica gel (AcOEt/hexane = 1 : 9). ^1^H NMR (400 MHz, CDCl_3_) *δ* 8.11–8.09 (m, 2H), 7.66–7.62 (m, 1H), 7.51–7.47 (m, 2H), 6.89 (s, 2H), 5.91 (ddd, *J* = 26.4, 4.8, 2.7 Hz, 1H), 2.81 (d, *J* = 4.8 Hz, 1H), 2.45 (bs, 6H), 2.23 (s, 3H); ^13^C NMR (CDCl_3_, 100 MHz) *δ* 191.5 (dd, *J* = 30.9, 28.9 Hz), 138.2, 134.5, 132.5, 130.2 (m), 128.6, 127.6, 117.6 (dd, *J* = 265.6, 252.4 Hz), 70.5 (dd, *J* = 30.5, 22.2 Hz), 21.2, 20.8; ^19^F NMR (376 MHz, CDCl_3_) *δ* −102.0 (dd, *J*_FF_ = 292, *J*_HF_ = 2.7 Hz, 1F), −114.6 (dd, *J*_FF_ = 292, *J*_HF_ = 26.4 Hz, 1F); HRMS (EI) *m*/*z* calcd for C_18_H_18_F_2_O_2_ [M]^+^ 304.1275, found 304.1279.

#### 2,2-Difluoro-3-hydroxy-1-phenyl-3-(pyridin-2-yl)propan-1-one (6am)

The titled product (6am) was obtained as a colorless solid in 39% yield (51.8 mg), after column chromatography on silica gel (AcOEt/hexane = 3 : 7). mp 82.5–83.5 °C (from Et_2_O–C_6_); ^1^H NMR (400 MHz, CDCl_3_) *δ* 8.61 (m, 1H), 8.10–8.08 (m, 2H), 7.81–7.76 (m, 1H), 7.64–7.60 (m, 1H), 7.53–7.46 (m, 3H), 7.37–7.34 (m, 1H), 5.37 (dd, *J* = 17.9, 4.9, 1H), 1.60 (bs, 1H); ^13^C NMR (CDCl_3_, 100 MHz) *δ* 190.4 (dd, *J* = 29.6, 26.6 Hz), 152.3, 148.1, 136.9, 134.1, 133.3, 130.2 (m), 128.5, 124.0, 123.1 (m), 116.7 (dd, *J* = 263.6, 257.7 Hz), 71.8 (dd, *J* = 29.2, 25.3 Hz); ^19^F NMR (376 MHz, CDCl_3_) *δ* −104.7 (dd, *J*_FF_ = 272, *J*_HF_ = 4.9 Hz, 1F), −116.7 (dd, *J*_FF_ = 272, *J*_HF_ = 17.9 Hz, 1F); HRMS (EI) *m*/*z* calcd for C_14_H_11_F_2_NO_2_ [M]^+^ 263.0758, found 263.0759.

#### 3-Cyclohexyl-2,2-difluoro-3-hydroxy-1-phenylpropan-1-one (6an)^4^

The titled product (6an) was obtained as a colorless liquid in 82% yield (109.7 mg), after column chromatography on silica gel (AcOEt/hexane = 1 : 9). ^1^H NMR (400 MHz, CDCl_3_) *δ* 8.10–8.08 (m, 2H), 7.66–7.62 (m, 1H), 7.52–7.48 (m, 2H), 4.06 (m, 1H), 2.33 (d, *J* = 6.9 Hz, 1H), 1.99–1.66 (m, 6H), 1.39–1.16 (m, 5H); ^13^C NMR (CDCl_3_, 100 MHz) *δ* 190.7 (m), 134.4, 132.5, 130.1 (m), 128.7, 117.7 (dd, *J* = 262.8, 257.8 Hz), 74.7 (dd, *J* = 26.2, 22.7 Hz), 38.1, 30.1, 27.3, 26.2, 26.1, 25.9; ^19^F NMR (376 MHz, CDCl_3_) *δ* −104.9 (dd, *J*_FF_ = 290, *J*_HF_ = 6.3 Hz, 1F), −114.4 (dd, *J*_FF_ = 290, *J*_HF_ = 19.8 Hz, 1F); HRMS (EI) *m*/*z* calcd for C_15_H_18_F_2_O_2_ [M]^+^ 268.1275, found 268.1280.

#### 2,2-Difluoro-3-hydroxy-1,5-diphenylpentan-1-one (6ao)^18^

The titled product (6ao) was obtained as a colorless liquid in 75% yield (108.4 mg), after column chromatography on silica gel (AcOEt/hexane = 1 : 9). ^1^H NMR (400 MHz, CDCl_3_) *δ* 8.10–8.08 (m, 2H), 7.66–7.62 (m, 1H), 7.51–7.47 (m, 2H), 7.32–7.18 (m, 5H), 4.23–4.19 (m, 1H), 2.99 (ddd, *J* = 13.8, 8.8, 5.2 Hz, 1H), 2.77 (ddd, *J* = 13.8, 8.8, 8.5 Hz, 1H), 2.56 (d, *J* = 5.6 Hz, 1H), 2.13–1.96 (m, 2H); ^13^C NMR (CDCl_3_, 100 MHz) *δ* 190.5 (dd, *J* = 31.7, 30.7 Hz), 141.0, 134.7, 132.1 (m), 130.2 (m) 128.7, 128.5, 126.1, 116.4 (dd, *J* = 266.2, 257.2 Hz), 70.4 (dd, *J* = 27.3, 24.3 Hz), 31.3, 30.3; ^19^F NMR (376 MHz, CDCl_3_) *δ* −107.3 (dd, *J*_FF_ = 298, *J*_HF_ = 5.3 Hz, 1F), −116.9 (dd, *J*_FF_ = 298, *J*_HF_ = 17.6 Hz, 1F); HRMS (EI) *m*/*z* calcd for C_17_H_16_F_2_O_2_ [M]^+^ 290.1118, found 290.1121.

#### 2,2-Difluoro-3-hydroxy-1-(4-methoxyphenyl)-3-phenylpropan-1-one (6ba)^4,8*c*^

The titled product (6ba) was obtained as a colorless solid in 69% yield (100.9 mg), after column chromatography on silica gel (AcOEt/hexane = 1 : 4). mp 84.5–85.0 °C (from Et_2_O–C_6_, lit. 68–69 °C); ^1^H NMR (400 MHz, CDCl_3_) *δ* 8.06 (d, *J* = 8.9 Hz, 2H), 7.51–7.49 (m, 2H), 7.41–7.37 (m, 3H), 6.94 (d, *J* = 8.9 Hz, 2H), 5.36 (ddd, *J* = 19.1, 5.2, 4.3 Hz, 1H), 3.89 (s, 3H), 3.16 (d, *J* = 4.3 Hz, 1H); ^13^C NMR (CDCl_3_, 100 MHz) *δ* 189.1 (dd, *J* = 31.3, 30.1 Hz), 164.8, 134.7, 132.9 (m), 128.9, 128.2, 128.1, 125.0, 115.7 (dd, *J* = 262.8, 256.8 Hz), 114.0, 73.3 (dd, *J* = 29.3, 23.1 Hz), 55.6; ^19^F NMR (376 MHz, CDCl_3_) *δ* −103.8 (dd, *J*_FF_ = 295, *J*_HF_ = 5.2 Hz, 1F), −115.8 (dd, *J*_FF_ = 295, *J*_HF_ = 19.1 Hz, 1F); HRMS (EI) *m*/*z* calcd for C_16_H_14_F_2_O_3_ [M]^+^ 292.0911, found 292.0912.

#### 1-(4-Chlorophenyl)-2,2-difluoro-3-hydroxy-3-phenylpropan-1-one (6ca)^4,8*c*^

The titled product (6ca) was obtained as a colorless solid in 85% yield (125.8 mg), after column chromatography on silica gel (AcOEt/hexane = 1 : 9). mp 115.5–116.0 °C (from CHCl_3_–C_6_, lit. 112–113 °C); ^1^H NMR (400 MHz, CDCl_3_) *δ* 7.98 (d, *J* = 8.7 Hz, 2H), 7.49–7.38 (m, 7H), 5.36 (ddd, *J* = 18.5, 5.9, 4.5 Hz, 1H), 2.92 (d, *J* = 4.5 Hz, 1H); ^13^C NMR (CDCl_3_, 100 MHz) *δ* 189.8 (dd, *J* = 31.0, 29.4 Hz), 141.3, 134.5, 131.6 (m), 130.7 (m), 129.2, 129.1, 128.4, 128.1, 115.7 (dd, *J* = 260.1, 256.0 Hz), 73.3 (dd, *J* = 27.6, 23.1 Hz); ^19^F NMR (376 MHz, CDCl_3_) *δ* −104.8 (dd, *J*_FF_ = 291, *J*_HF_ = 5.9 Hz, 1F), −116.3 (dd, *J*_FF_ = 291, *J*_HF_ = 18.5 Hz, 1F); HRMS (EI) *m*/*z* calcd for C_15_H_11_ClF_2_O_2_ [M]^+^ 296.0416, found 296.0411 (55.1), 298.0392 (18.4).

#### 1-Cyclohexyl-2,2-difluoro-3-hydroxy-3-phenylpropan-1-one (6da)^4^

The titled product (6da) was obtained as a colorless solid in 79% yield (106.6 mg), after column chromatography on silica gel (AcOEt/hexane = 1 : 9). mp 58.0–59.0 °C (from petroleum ether, lit. 58–60 °C); NMR (400 MHz, CDCl_3_) *δ* 7.42–7.37 (m, 5H), 5.17 (ddd, *J* = 16.5, 7.7, 4.8 Hz, 1H), 2.77–2.72 (m, 1H), 2.69 (d, *J* = 4.8 Hz, 1H), 1.84–1.64 (m, 5H), 1.37–1.16 (m, 5H); ^13^C NMR (CDCl_3_, 100 MHz) *δ* 205.5 (dd, *J* = 30.3, 26.7 Hz), 134.8, 129.0, 128.4, 127.8, 115.0 (dd, *J* = 262.7, 256.9 Hz), 73.1 (dd, *J* = 27.2, 23.9 Hz), 27.8, 27.7, 25.5, 25.3, 25.2; ^19^F NMR (376 MHz, CDCl_3_) *δ* −112.4 (dd, *J*_FF_ = 273, *J*_HF_ = 7.7 Hz, 1F), −122.2 (dd, *J*_FF_ = 273, *J*_HF_ = 16.5 Hz, 1F); HRMS (EI) *m*/*z* calcd for C_15_H_18_F_2_O_2_ [M]^+^ 268.1275, found 268.1274.

#### 2,2-Difluoro-3-hydroxy-1,3-diphenylbutan-1-one (6ap)^4^

The titled product (6ap) was obtained as a colorless liquid in 60% yield (82.5 mg), after column chromatography on silica gel (AcOEt/hexane = 1 : 9). ^1^H NMR (400 MHz, CDCl_3_) *δ* 7.92–7.90 (m, 2H), 7.58–7.54 (m, 3H), 7.41–7.28 (m, 5H), 3.54 (s, 1H), 1.82 (t, *J* = 1.4 Hz, 3H); ^13^C NMR (CDCl_3_, 100 MHz) *δ* 191.6 (t, *J* = 31.0 Hz), 140.2, 134.3, 133.1, 130.2 (m), 128.4, 128.1, 128.0, 126.3, 116.5 (t, *J* = 263.0 Hz), 76.4 (t, *J* = 24.4 Hz), 24.0 (t, *J* = 2.9 Hz); ^19^F NMR (376 MHz, CDCl_3_) *δ* −107.4 (m, 2F); HRMS (EI) *m*/*z* calcd for C_16_H_14_F_2_O_2_ [M]^+^ 276.0962, found 276.0968.

#### 2,2-Difluoro-3-hydroxy-3-methyl-1-phenylbutan-1-one (6aq)^4^

The titled product (6aq) was obtained as a colorless liquid in 53% yield (57.0 mg), after column chromatography on ODS-silica gel (H_2_O/MeOH = 2 : 3). ^1^H NMR (400 MHz, CDCl_3_) *δ* 8.13–8.11 (m, 2H), 7.66–7.62 (m, 1H), 7.52–7.48 (m, 2H), 2.79 (bs, 1H), 1.45 (t, *J* = 1.5 Hz, 6H); ^13^C NMR (CDCl_3_, 100 MHz) *δ* 191.2 (t, *J* = 31.0 Hz), 134.5, 130.0 (m), 130.4 (m), 128.6, 116.9 (t, *J* = 261.6 Hz), 73.3 (t, *J* = 24.7 Hz), 23.6 (t, *J* = 2.6 Hz); ^19^F NMR (376 MHz, CDCl_3_) *δ* −110.5 (m, 2F); HRMS (EI) *m*/*z* calcd for C_11_H_12_F_2_O_2_ [M]^+^ 214.0805, found 214.0799.

## Conflicts of interest

There are no conflicts to declare.

## Supplementary Material

RA-008-C8RA02440E-s001
