# Supplementary material for: Decarboxylative aldol reaction of α,α-difluoro-β-ketocarboxylate salt: a facile method for generation of difluoroenolate
Source: RSC Adv. 2018 Jun 5;8(37):20568–75. doi: 10.1039/c8ra02440e (PMC9080823; doi:10.1039/c8ra02440e)
Supplement: RA-008-C8RA02440E-s001 [file RA-008-C8RA02440E-s001.pdf]

Electronic Supplementary Material (ESI) for RSC Advances.
This journal is © The Royal Society of Chemistry 2018

Supporting Information

Decarboxylative aldol reaction of α,α-difluoro-β-ketocarboxylate salt: a facile method for generation of difluoroenolate

Atsushi Tarui, Mayuna Oduti, Sususmu Shinya, Kazuyuki Sato and Masaaki Omote*
Faculty of Pharmaceutical Sciences, Setsunan University, 45-1 Nagaotogecho, Hirakata, Osaka 573-0101, Japan

Table of Contents

General Information 1
Preparation of potassium α,α-difluoro-β-ketopropanoate (**2**) 1−6
Screening of Solvents 7
Screening of counter cation of **2a** and the loading of $ZnCl_2$/TMEDA 7
References 8
NMR Spectra of Compounds 9−56

General information

NMR spectra were obtained from a solution in $CDCl_3$ using 400 MHz for ^1H, 100 MHz for ^{13}C, 376 MHz for ^{19}F. Chemical shifts of ^1H and ^{13}C NMR are reported in ppm from tetramethylsilane (TMS) as an internal standard. Chemical shifts of ^{19}F NMR are reported in ppm from $CFCl_3$ as an internal standard. All data are reported as follows: chemical shifts, multiplicity (s = singlet, bs = broad singlet, d = doublet, t = triplet, q = quartet, dd = double doublet doublet, ddd = double double doublet, m = multiplet), coupling constants (Hz), and relative integration value. HRMS experiments were measured on a double-focusing mass spectrometer with an ionization mode of EI or negative FAB using nitrobenzyl alcohol (NBA) as a matrix. Melting points were measured uncorrected.

All experiments were carried out under argon atmosphere in flame-dried glassware using standard inert techniques for introducing reagents and solvents unless otherwise noted. Tetrahydrofuran (THF) was purchased from Kanto Chemical Co. Inc. as "Dehydrated". All commercially available materials were used as received without further purification. Toluene, 1,4-dioxane and *tert*-butyl methyl ether was distilled over sodium benzophenone ketyl just before use. CH_3CN and DMF was distilled over CaH_2 just before use.

Preparation of potassium α,α-difluoro-β-ketopropanoate (2)

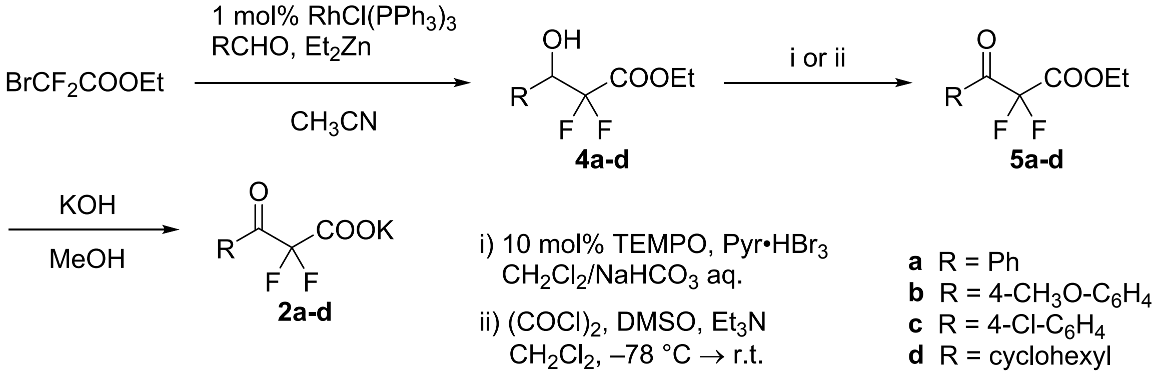

Reformatsky adduct 4

Ethyl bromodifluoroacetate (15 mmol) and the corresponding aldehydes (10 mmol) were added to a solution of $RhCl(PPh_3)_3$ (1 mol%) in CH_3CN (80 mL) at 0 °C, and stirred for 0.5 hr. 1.0 M Et_2Zn in hexane (15 mmol) was slowly added to the solution via dropfunnel, and then the whole mixture was stirred at room temperature. The reaction was quenched by aqueous 10% HCl and the mixture was extracted with AcOEt, and then the extract was washed with brine and dried with $MgSO_4$. The solvent was removed *in vacuo* and the residue was purified by column chromatography on silica gel to give the corresponding Reformatsky adduct **4**.

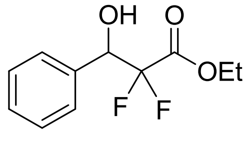

Ethyl 2,2-difluoro-3-hydroxy-3-phenylpropanoate (4a)[1]

The titled product (**4a**) was obtained as a colorless liquid in 95% yield (2.2 g), after column chromatography on silica gel (AcOEt/hexane = 1:4).

^1H NMR (400 MHz, $CDCl_3$) δ 7.55-7.36 (m, 5H), 5.14 (ddd, J = 15.4, 7.8, 5.4 Hz, 1H), 4.28 (q, J = 7.0 Hz, 2H), 2.96 (d, J = 5.4 Hz, 1H), 1.27 (t, J = 7.0 Hz, 3H); ^{13}C NMR ($CDCl_3$, 100 MHz) δ 163.6 (t, J = 31.8 Hz), 134.4, 129.3,

128.4, 127.7, 113.7 (dd, J = 259.4, 254.1 Hz), 73.7 (dd, J = 27.7, 24.3 Hz), 63.2, 13.8; ^{19}F NMR (376 MHz, $CDCl_3$) δ −113.8 (dd, J_{FF} = 262, J_{HF} = 7.8 Hz, 1F), −120.2 (dd, J_{FF} = 262, J_{HF} = 15.4 Hz, 1F).

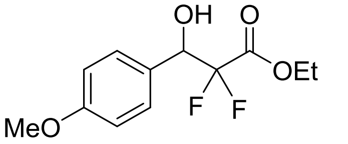

Ethyl 2,2-difluoro-3-hydroxy-3-(4-methoxyphenyl)propanoate (4b)[1]

The reaction was carried out using 3 mmol scale of 4-methoxybenzaldehyde. The titled product (**4b**) was obtained as a colorless liquid in 96% yield (749.8 mg), after column chromatography on silica gel (AcOEt/hexane = 1:4).

^{1}H NMR (400 MHz, $CDCl_3$) δ 7.36 (d, J = 8.6 Hz, 2H), 6.91 (d, J = 8.6 Hz, 2H), 5.11 (ddd, J = 15.3, 8.2, 5.2 Hz, 1H), 4.31 (q, J = 7.0 Hz, 2H), 3.82 (s, 1H), 2.60 (d, J = 5.2 Hz, 1H), 1.30 (t, J = 7.0 Hz, 3H); ^{13}C NMR ($CDCl_3$, 100 MHz) δ 163.6 (t, J = 31.3 Hz), 160.3, 129.0, 126.4, 113.9 113.7 (dd, J = 262.9, 254.0 Hz), 73.5 (dd, J = 27.1, 24.3 Hz), 63.1, 55.3, 13.9; ^{19}F NMR (376 MHz, $CDCl_3$) δ −114.2 (dd, J_{FF} = 261, J_{HF} = 8.2 Hz, 1F), −120.3 (dd, J_{FF} = 261, J_{HF} = 15.3 Hz, 1F).

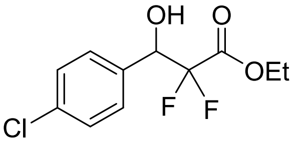

Ethyl 3-(4-chlorophenyl)-2,2-difluoro-3-hydroxypropanoate (4c)[2]

The reaction was carried out using 4 mmol scale of 4-chlorobenzaldehyde. The titled product (**4c**) was obtained as a colorless solid in 93% yield (882.9 mg), after column chromatography on silica gel (AcOEt/hexane = 1:4).

Mp: 34.0-35.5°C (from Et_2O-C_6); ^{1}H NMR (400 MHz, $CDCl_3$) δ 7.40-7.35 (m, 4H), 5.15 (dd, J = 15.3, 7.5 Hz, 1H), 4.32 (q, J = 7.2 Hz, 2H), 2.42 (bs, 1H), 1.31 (t, J = 7.2 Hz, 3H); ^{13}C NMR ($CDCl_3$, 100 MHz) δ 163.4 (t, J = 31.7 Hz), 135.2, 132.8, 129.0, 128.6, 113.5 (dd, J = 259.9, 254.6 Hz), 73.2 (dd, J = 27.9, 24.6 Hz), 63.2, 13.8; ^{19}F NMR (376 MHz, $CDCl_3$) δ −113.1 (dd, J_{FF} = 264, J_{HF} = 7.5 Hz, 1F), −120.5 (dd, J_{FF} = 264, J_{HF} = 15.3 Hz, 1F).

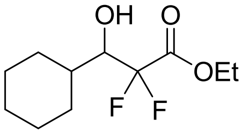

Ethyl 3-cyclohexyl-2,2-difluoro-3-hydroxypropanoate (4d)[2]

The reaction was carried out using 8 mmol scale of cyclohexanecarboxyaldehyde. The titled product (**4d**) was obtained as a colorless liquid in 81% yield (1.53 g), after column chromatography on silica gel (AcOEt/hexane = 1:4).

^{1}H NMR (400 MHz, $CDCl_3$) δ 4.35 (q, J = 7.2 Hz, 2H), 3.84-3.80 (m, 1H), 2.07-2.06 (m, 1H), 1.93-1.91 (m, 1H), 1.79-1.66 (m, 5H), 1.36 (t, J = 7.2 Hz, 3H), 1.30-1.10 (m, 5H); ^{13}C NMR ($CDCl_3$, 100 MHz) δ 163.9 (t, J = 32.0 Hz), 115.4 (t, J = 256.5 Hz), 75.2 (t, J = 25.0 Hz), 63.0, 38.2, 29.6, 27.3, 26.1, 26.0, 25.8, 13.9; ^{19}F NMR (376 MHz, $CDCl_3$) δ −111.5 (dd, J_{FF} = 263, J_{HF} = 7.9 Hz, 1F), −120.0 (dd, J_{FF} = 263, J_{HF} = 17.8 Hz, 1F). HRMS (EI) *m/z* calcd for $C_{11}H_{18}F_2O_3$ $[M]^+$ 236.1224, found 236.1229.

Oxidation of Reformatsky adduct 4

2,2,6,6-Tetramethylpiperidine 1-oxyl (TEMPO, 10 mol%) was added to a solution of Reformatsky adduct **4** (8.8 mmol) in CH_2Cl_2 (53 mL) at room temperature, then aqueous $NaHCO_3$ (5%, 123 mL) was added at same temperature. Finally, pyridine hydrobromide perbromide (13.2 mmol) was added to the mixture and the whole mixture was stirred vigorously for 2 hr. The reaction was quenched by aqueous 5% $Na_2S_2O_3$ and the mixture was extracted with AcOEt, and then the extract was washed with brine and dried with $MgSO_4$. The solvent was removed *in vacuo* and the residue was purified by column chromatography on silica gel to give the corresponding Ethyl 2,2-difluoro-3-oxo-3-arylpropionate **5**.

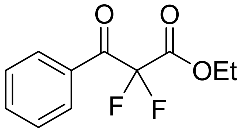

Ethyl 2,2-difluoro-3-oxo-3-phenylpropanoate (5a)[1]

The titled product (**5a**) was obtained as a colorless liquid in 84% yield (1.7 g), after column chromatography on silica gel (AcOEt/hexane = 1:19).

^{1}H NMR (400 MHz, $CDCl_3$) δ 8.09-8.07 (m, 2H), 7.70-7.66 (m, 1H), 7.55-7.51 (m, 2H), 4.39 (q, *J* = 7.2 Hz, 2H), 1.32 (t, *J* = 7.2 Hz, 3H); ^{13}C NMR ($CDCl_3$, 100 MHz) δ 185.5 (t, *J* = 27.5 Hz), 161.8 (t, *J* = 30.3 Hz), 135.1, 131.0, 129.9 (m), 128.9, 109.8 (t, *J* = 264.0 Hz), 63.8, 13.8; ^{19}F NMR (376 MHz, $CDCl_3$) δ −44.9 (s, 2F).

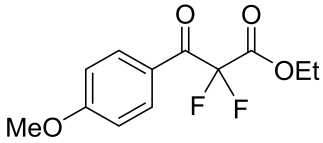

Ethyl 2,2-difluoro-3-(4-methoxyphenyl)-3-oxopropanoate (5b)[1]

The reaction was carried out using 2.9 mmol scale of **4b**. The titled product (**5b**) was obtained as a colorless liquid in 94% yield (706.7 mg), after column chromatography on silica gel (AcOEt/hexane = 1:19).

^{1}H NMR (400 MHz, $CDCl_3$) δ 8.09-8.07 (d, *J* = 8.5 Hz, 2H), 6.99-6.97 (d, *J* = 8.5 Hz, 2H), 4.38 (q, *J* = 7.1 Hz, 2H), 3.91 (s, 3H), 1.32 (t, *J* = 7.1 Hz, 3H); ^{13}C NMR ($CDCl_3$, 100 MHz) δ 183.8 (t, *J* = 27.5 Hz), 165.1, 162.1 (t, *J* = 30.5 Hz), 132.6 (m), 123.9, 114.3, 110.1 (t, *J* = 264 Hz), 63.7, 55.7, 13.9; ^{19}F NMR (376 MHz, $CDCl_3$) δ −107.1 (s, 2F). HRMS (EI) *m/z* calcd for $C_{12}H_{12}F_2O_4$ $[M]^+$ 258.0704, found 258.0706

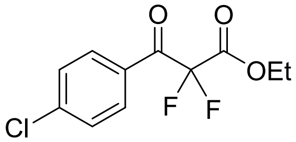

Ethyl 3-(4-chlorophenyl)-2,2-difluoro-3-oxopropanoate (5c)

The reaction was carried out using 3.0 mmol scale of **4c**. The titled product (**5c**) was obtained as a colorless liquid in 81% yield (642.0 mg), after column chromatography on silica gel (AcOEt/hexane = 1:19).

^{1}H NMR (400 MHz, $CDCl_3$) δ 8.03 (d, *J* = 8.7 Hz, 2H), 7.51 (d, *J* = 8.7 Hz, 2H), 4.40 (q, *J* = 7.2 Hz, 2H), 1.33 (t, *J* = 7.2 Hz, 3H); ^{13}C NMR ($CDCl_3$, 100 MHz) δ 184.5 (t, *J* = 27.9 Hz), 161.6 (t, *J* = 30.3 Hz), 142.0, 131.3 (m), 129.4, 129.3, 109.7 (t, *J* = 265 Hz), 63.9, 13.8; ^{19}F NMR (376 MHz, $CDCl_3$) δ −107.2 (s, 2F). HRMS (EI) *m/z* calcd for $C_{11}H_9ClF_2O_3$ $[M]^+$ 262.0208, found 262.0217 (100), 264.0169(35.8).

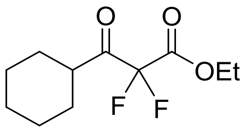

Ethyl 3-cyclohexyl-2,2-difluoro-3-oxopropanoate (5d)[1]

To a dry and argon-flushed reaction vessel, equipped with a magnetic stirrer, were added CH_2Cl_2 (28 mL) and oxalyl chloride (13 mmol), then the solution was cooled to -78 °C. Dimethyl sulfoxide (26 mmol) was added slowly to the cold solution. After the whole mixture was stirred for 0.5 hr, the solution of **4d** (6.5 mmol) in CH_2Cl_2 (18 mL) was added slowly to the reaction mixture. Finally, triethylamine (32.5 mmol) was added slowly to the above mixture. The whole mixture was warmed up to room temperature and was stirred at same temperature for 0.5 hr. The reaction was quenched by aqueous saturated NH_4Cl and the mixture was extracted with AcOEt, and then the extract was washed with brine and dried with $MgSO_4$. The solvent was removed *in vacuo* and the residue was purified by column chromatography (AcOEt/C_6 = 1:19) on silica gel to give Ethyl 2,2-difluoro-3-oxo-3-cyclohexylpropionate **5d** as a colorless solid in 98% yield (1.49 g).

^{1}H NMR (400 MHz, $CDCl_3$) δ 4.36 (q, J = 7.2 Hz, 2H), 2.93-2.87 (m, 1H), 1.69-1.92 (m, 5H), 1.42-1.22 (m, 8H); ^{13}C NMR ($CDCl_3$, 100 MHz) δ 200.2 (t, J = 27.0 Hz), 161.6 (t, J = 30.8 Hz), 108.6 (t, J = 265 Hz), 63.6, 45.3, 28.1, 25.5, 25.3, 13.9; ^{19}F NMR (376 MHz, $CDCl_3$) δ −113.0 (s, 2F). HRMS (EI) *m/z* calcd for $C_{11}H_{16}F_2O_3$ $[M]^+$ 234.1068, found 234.1072.

Saponification of α,α-difluoro-β-keto ester (5)

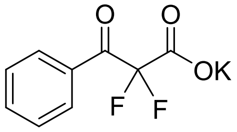

Potassium 2,2-difluoro-3-oxo-3-phenylpropanoate (2a)[1]

The solution of 3-substituted Ethyl 2,2-difluoro-3-oxopropionate **5a** (14.6 mmol) in MeOH (10 mL) was cooled to 0 °C, then pre-cooled solution of KOH in MeOH (14.6 mmol, 4.6 mL) was added slowly via syringe. The whole mixture was stirred at room temperature for 6 hr. The reaction mixture was concentrated *in vacuo*, then the residue was suspended by AcOEt/Et_2O (= 1:1). The suspension was sonicated for 10 min then the precipitate was corrected by suction filtration. The filtrate was dried under reduce pressure to give potassium 2,2-difluoro-3-oxo-3-arylpropionate **2a** in 80% yield (2.76 g) without purification.

^{1}H NMR (400 MHz, DMSO-d6) δ 8.02-8.00 (m, 2H), 7.67-7.64 (m, 1H), 7.54-7.50 (m, 2H); ^{13}C NMR (DMSO-d6, 100 MHz) δ 189.6 (t, J = 27.5 Hz), 162.4 (m), 133.7, 132.6, 129.1, 128.5, 111.3 (t, J = 264 Hz); ^{19}F NMR (376 MHz, $CDCl_3$) δ −105.2 (s, 2F). HRMS (FAB, NBA) *m/z* calcd for $C_9H_5F_2O_3$ $[M{-}K]^-$ 199.0212, found 199.0205.

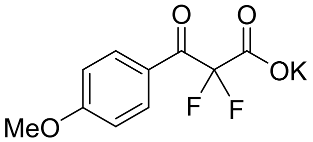

Potassium 2,2-difluoro-3-(4-methoxyphenyl)-3-oxopropanoate (2b)[1]

The reaction was carried out using 3.3 mmol scale of **5b**. The titled product (**2b**) was obtained as a colorless solid in 89% yield (786.0 mg) without purification.

^{1}H NMR (400 MHz, DMSO-d6) δ 8.00 (d, *J* = 8.8 Hz, 2H), 7.04 (d, *J* = 8.8 Hz, 2H), 3.84 (s, 3H); ^{13}C NMR (DMSO-d6, 100 MHz) δ 187.9 (t, *J* = 27.5 Hz), 163.4, 162.5 (t, *J* = 24.1 Hz), 131.5, 125.3, 113.7, 111.3 (t, *J* = 262 Hz), 55.5; ^{19}F NMR (376 MHz, $CDCl_3$) δ −104.8 (s, 2F). HRMS (FAB, NBA) *m/z* calcd for $C_{10}H_7F_2O_4$ $[M-K]^-$ 229.0318, found 229.0317.

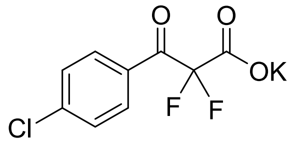

Potassium 3-(4-chlorophenyl)-2,2-difluoro-3-oxopropanoate (2c)

The reaction was carried out using 2.2 mmol scale of **5c**. The titled product (**2c**) was obtained as a colorless solid in 78% yield (482.5 mg) without purification.

^{1}H NMR (400 MHz, DMSO-d6) δ 8.00 (d, *J* = 8.5 Hz, 2H), 7.62 (d, *J* = 8.5 Hz, 2H); ^{13}C NMR (DMSO-d6, 100 MHz) δ 188.7 (t, *J* = 28.4 Hz), 162.0 (t, *J* = 23.6 Hz), 138.7, 131.2, 130.9, 128.7, 111.1 (t, *J* = 263 Hz); ^{19}F NMR (376 MHz, DMSO-d6) δ −105.4 (s, 2F). HRMS (FAB, NBA) *m/z* calcd for $C_9H_4ClF_2O_3$ $[M-K]^-$ 232.9823, found 232.9821.

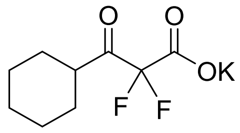

Potassium 3-cyclohexyl-2,2-difluoro-3-oxopropanoate (2d)[1]

The reaction was carried out using 6.3 mmol scale of **5d**. The titled product (**2d**) was obtained as a colorless solid in 79% yield (1.24 g) without purification.

^{1}H NMR (400 MHz, DMSO-d6) δ 2.72-2.67 (m, 1H), 1.77-1.60 (m, 5H), 1.28-1.08 (m, 5H); ^{13}C NMR (DMSO-d6, 100 MHz) δ 203.8 (t, *J* = 26.0 Hz), 162.0 (t, *J* = 24.5 Hz), 111.1 (t, *J* = 265 Hz), 44.7, 28.1, 25.2, 24.9; ^{19}F NMR (376 MHz, DMSO-d6) δ −111.0 (s, 2F). HRMS (FAB, NBA) *m/z* calcd for $C_9H_{11}F_2O_3$ $[M-K]^-$ 205.0682, found 205.0666.

Table S-1. Screening of counter cation of 2a and the loading of $ZnCl_2$/TMEDA.

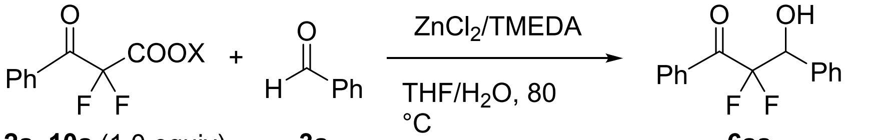

2a–10a (1.0 equiv) **3a** **6aa**

Entry	Substrates	X	$ZnCl_2$• TMEDA (equiv)	Time (h)	Yield of **6aa**[a]
1	**2a**	K	1.0	5	88
2	**9a**	Na	1.0	16	83
3	**10a**	Li	1.0	16	75
4	**2a**	K	0.5	24	82
5	**2a**	K	0.1	24	39

a: Isolated yields.

Table S-2. Screening of solvent.

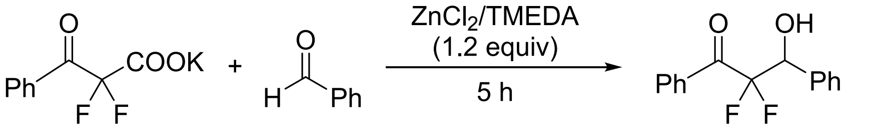

2a (1.2 equiv) **3a** **6aa**

Entry	Solvents	Temp. (°C)	Yield of **6aa** (%)[a]
1	THF (optimized condition)	80	(98)[b]
2	THF	80	42
3	*tert*-butyl methyl ether	65	0
4	*tert*-butyl methyl ether	65	0[b]
5	1,4-dioxane	115	(80)
6	1,4-dioxane	115	(90)[b]
7	CH_3CN	95	57
8	CH_3CN	95	53[b]
9	CH_3CN/THF (1:1)	95	72
10	CH_3CN/THF (1:5)	95	80
11	DMF	165	0
12	DMF	165	0[b]
13	toluene	110	52
14	toluene	110	48[b]

a: ^{19}F NMR yields. Isolated yield was show in parenthesis.

b: H_2O (1.0 equiv) was added to the corresponding solvent.

References

1. M.-H. Yang, D. L. Orsi and R. A. Altman, *Angew. Chem. Int. Ed.* **2015**, *54*, 2361-2365.
2. Y. Wang, F. Xing, C.-Z. Gu, W.-J. Li, L. He, B. Dai and G.-F. Du, *Tetrahedron*, **2017**, *73*, 4501-4507.

NMR Spectra of Compounds (^{1}H, ^{13}C and ^{19}F NMR)

Ethyl 2,2-difluoro-3-hydroxy-3-phenylpropanoate (4a).

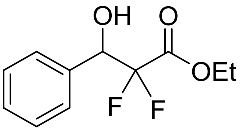

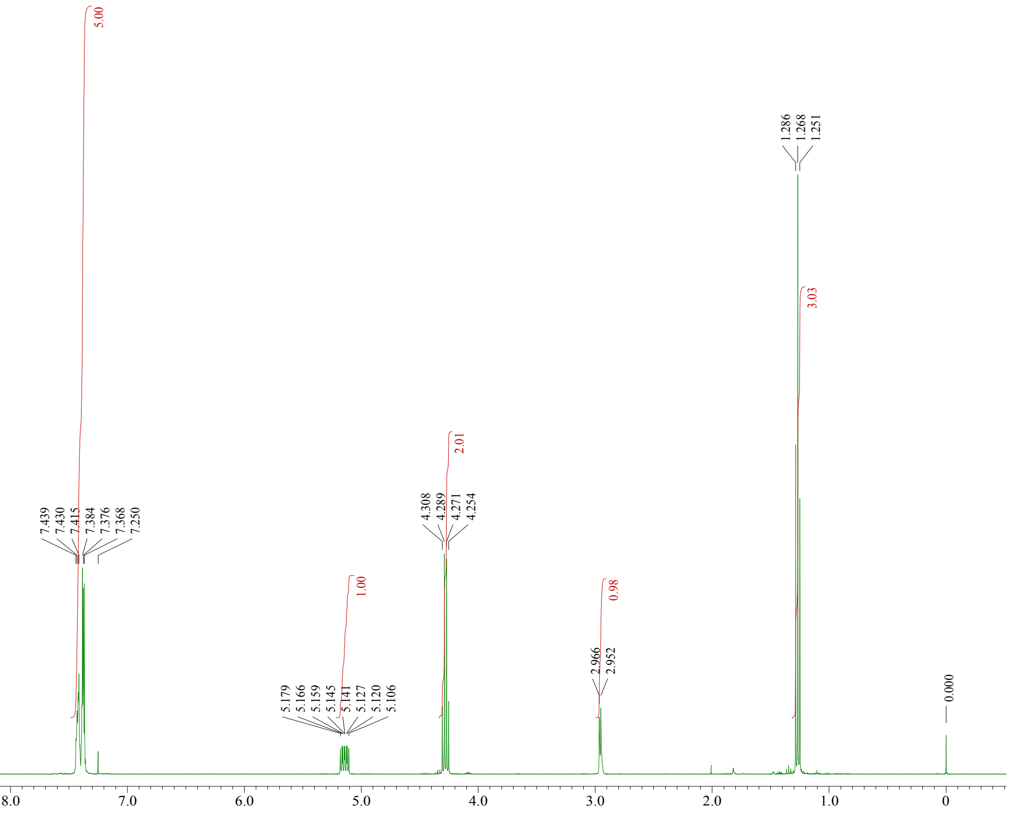

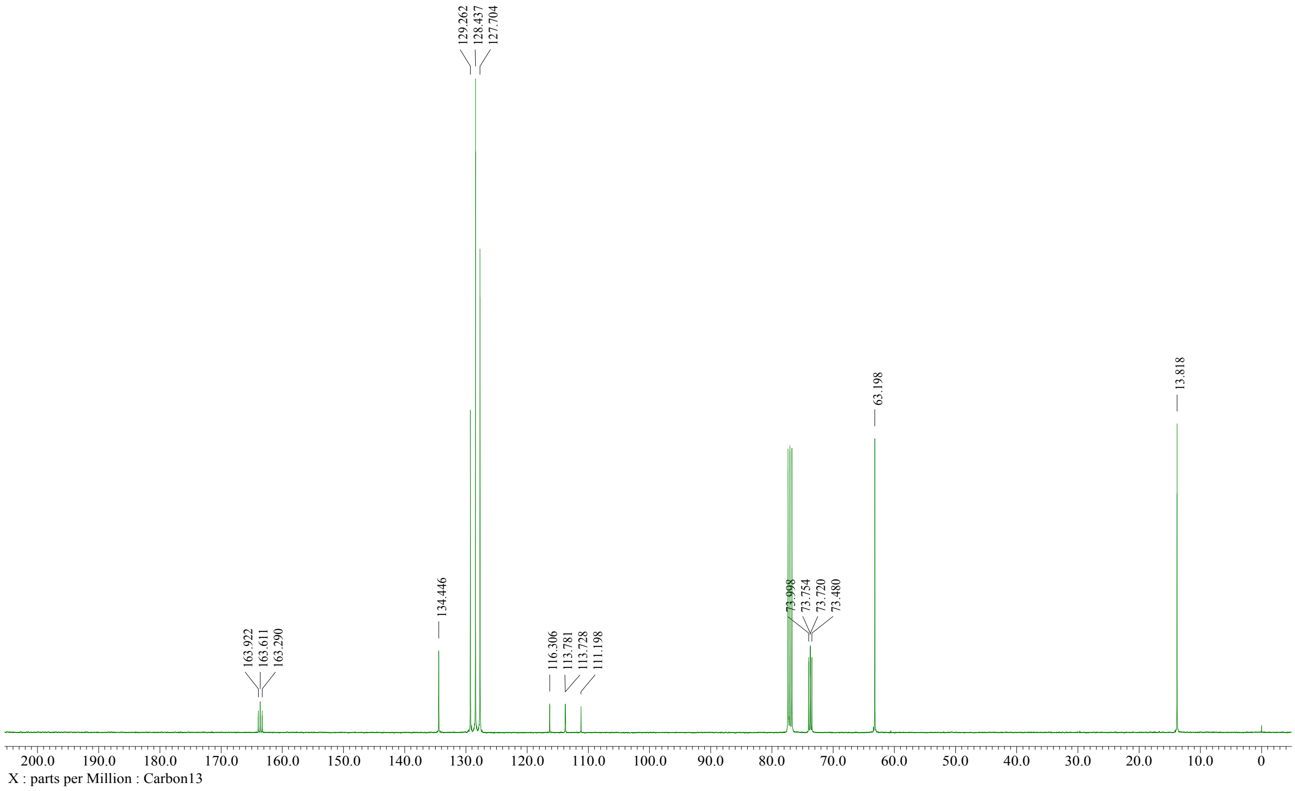

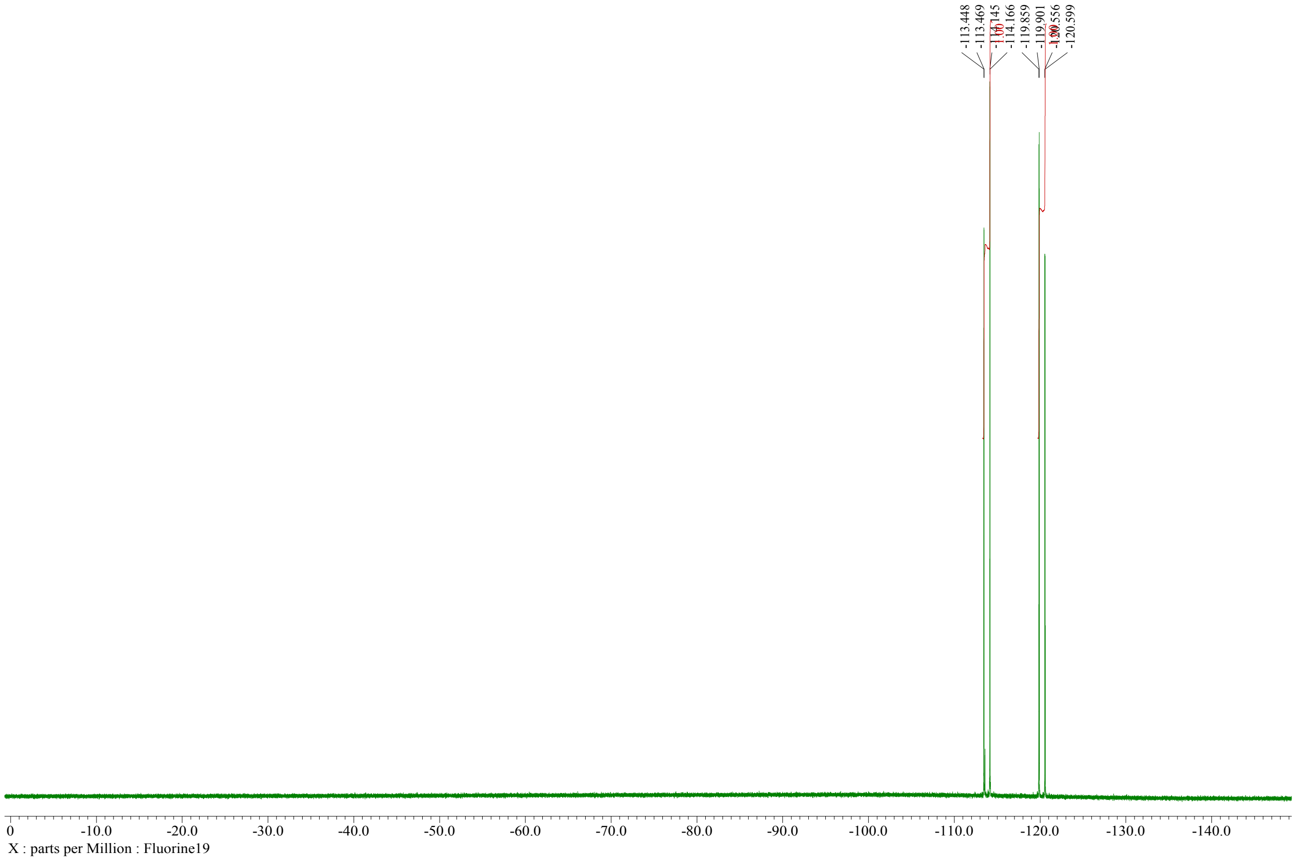

2,2-Difluoro-3-hydroxy-1-(4-methoxyphenyl)-3-phenylpropan-1-one (4b)

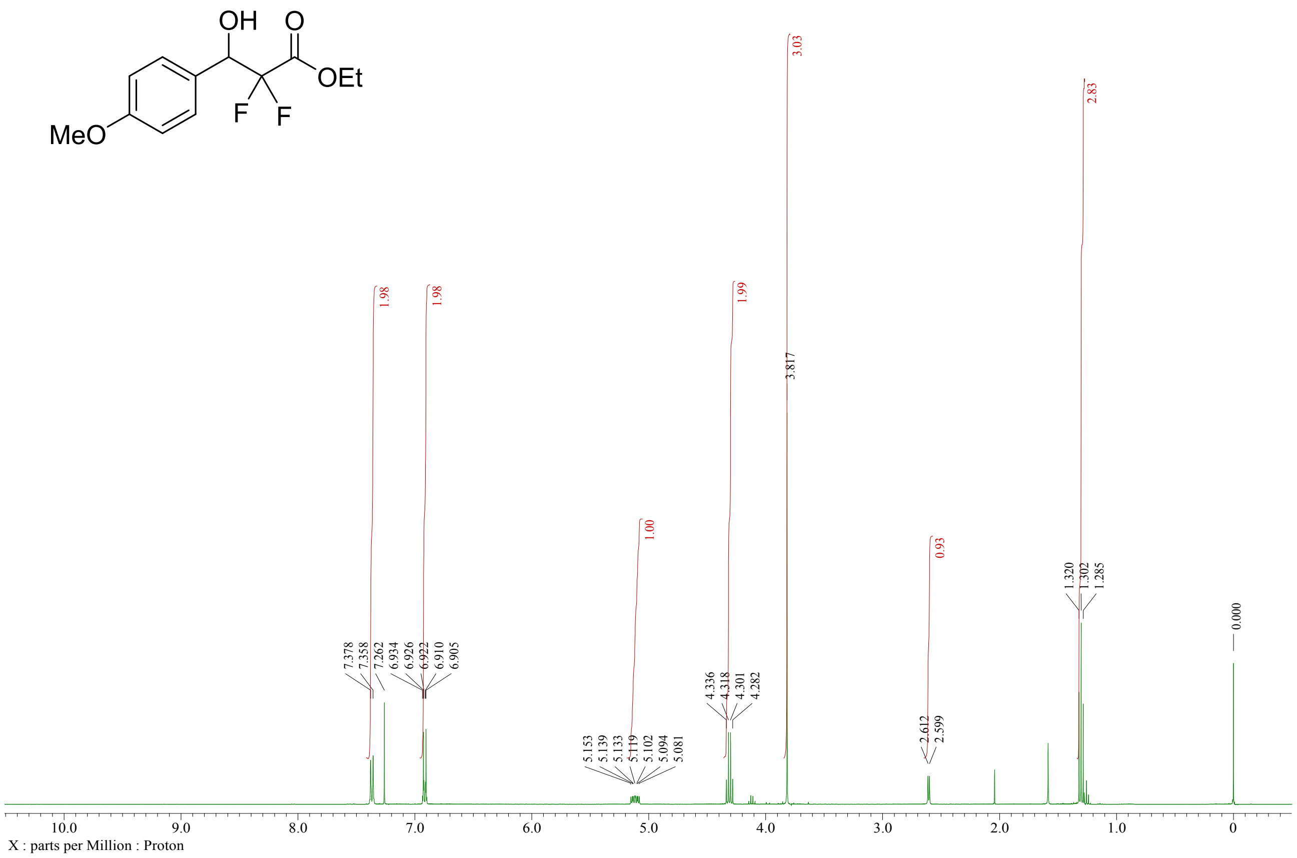

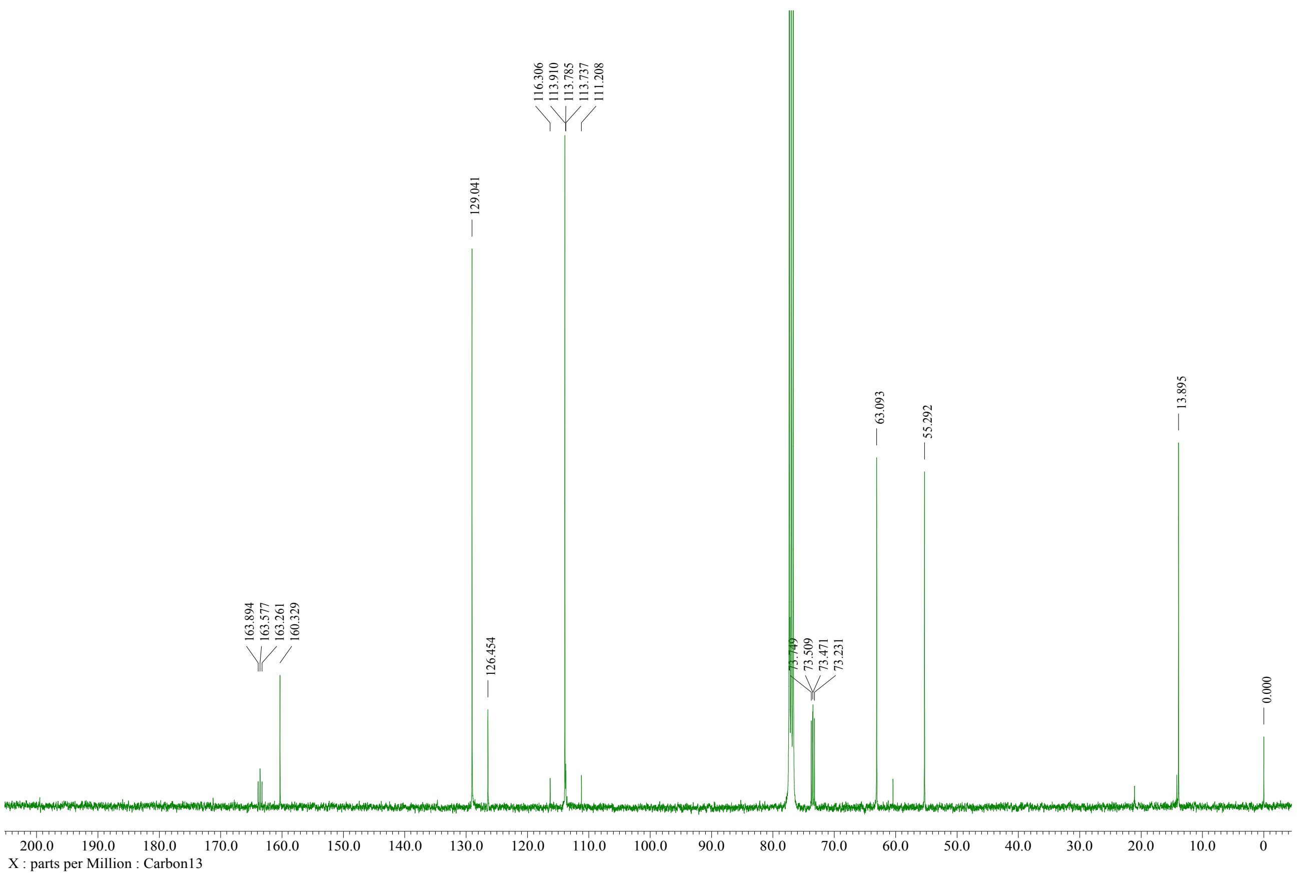
163.894
163.577
163.261
160.329
129.041
126.454
116.306
113.910
113.785
113.737
111.208
73.749
73.509
73.471
73.231
63.093
55.292
13.895
0.000
200.0 190.0 180.0 170.0 160.0 150.0 140.0 130.0 120.0 110.0 100.0 90.0 80.0 70.0 60.0 50.0 40.0 30.0 20.0 10.0 0
X : parts per Million : Carbon13

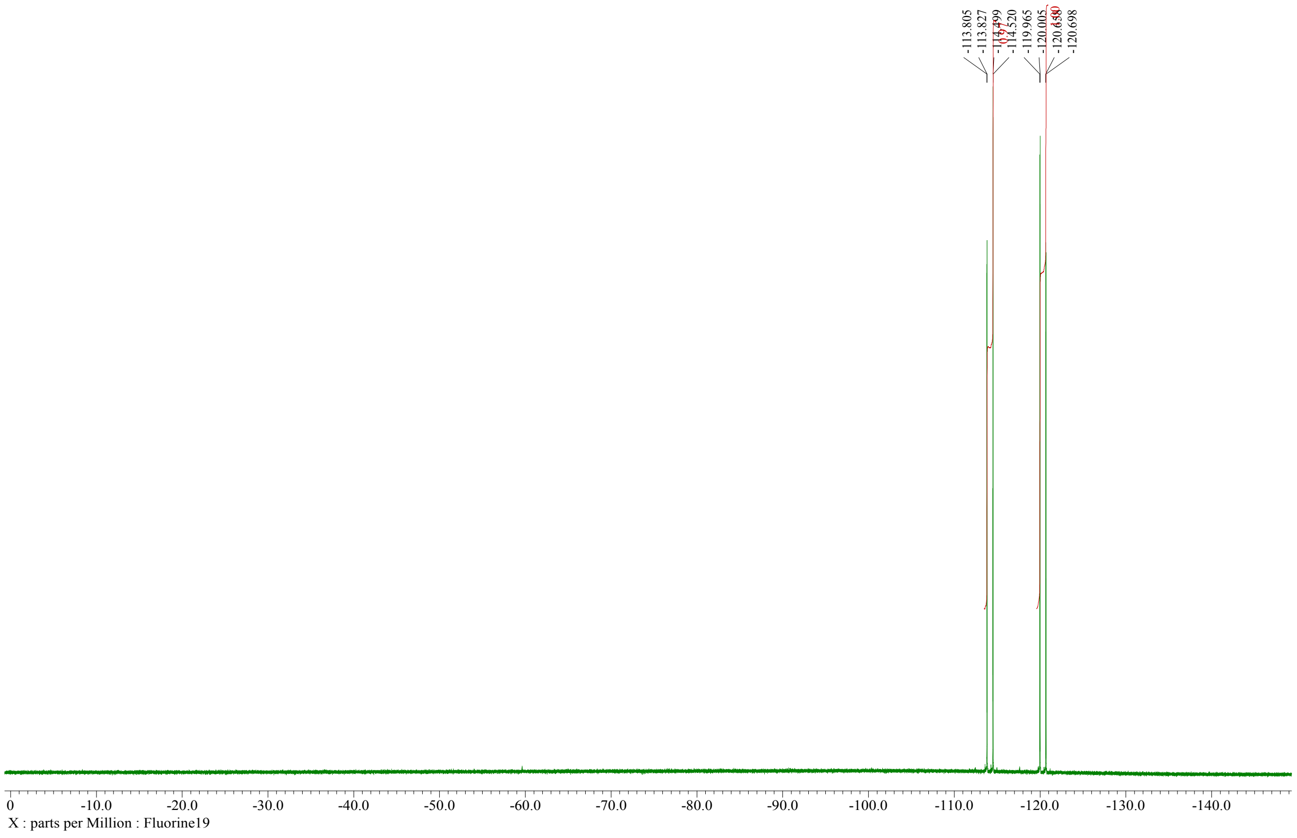
-113.805
-113.827
-114.520
-119.965
-120.005
-120.698
0 -10.0 -20.0 -30.0 -40.0 -50.0 -60.0 -70.0 -80.0 -90.0 -100.0 -110.0 -120.0 -130.0 -140.0
X : parts per Million : Fluorine19

Ethyl 3-(4-chlorophenyl)-2,2-difluoro-3-hydroxypropanoate (4c)

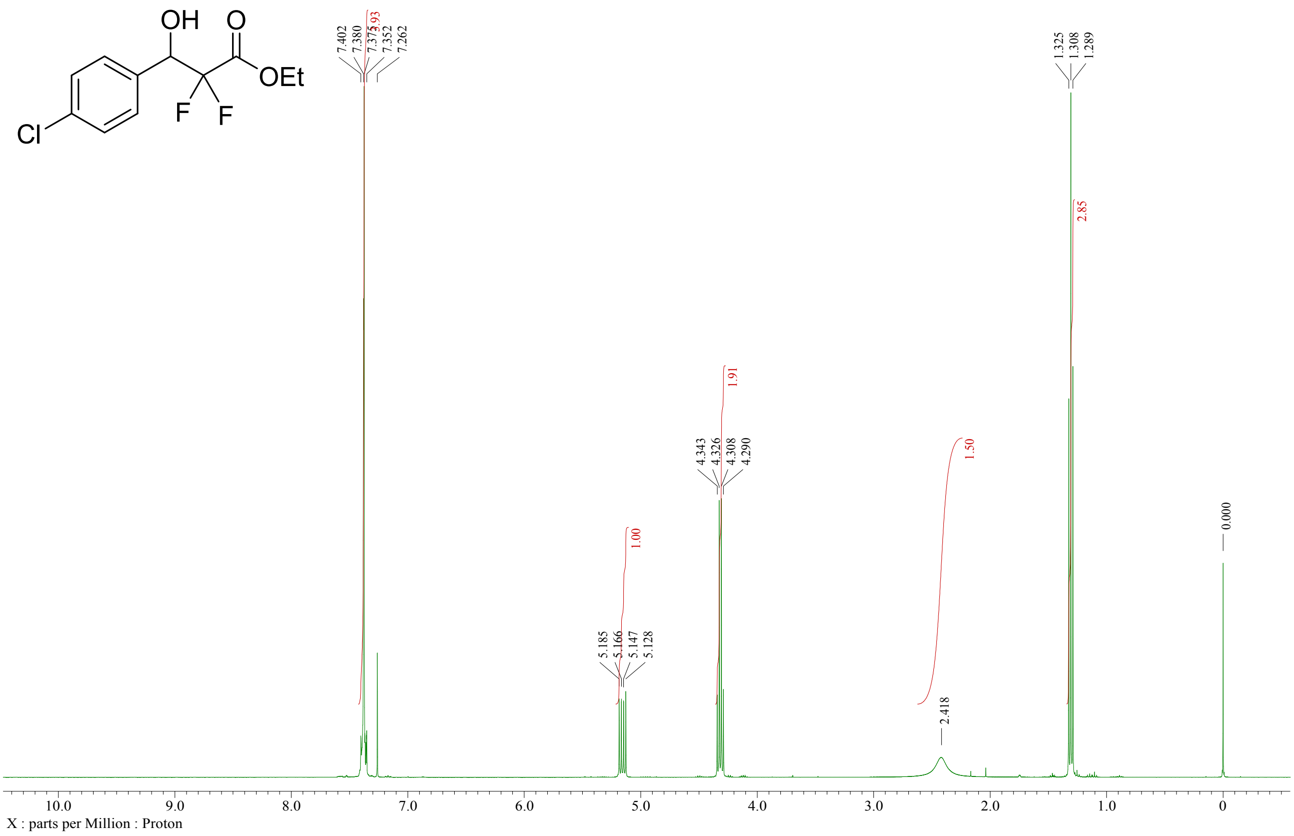

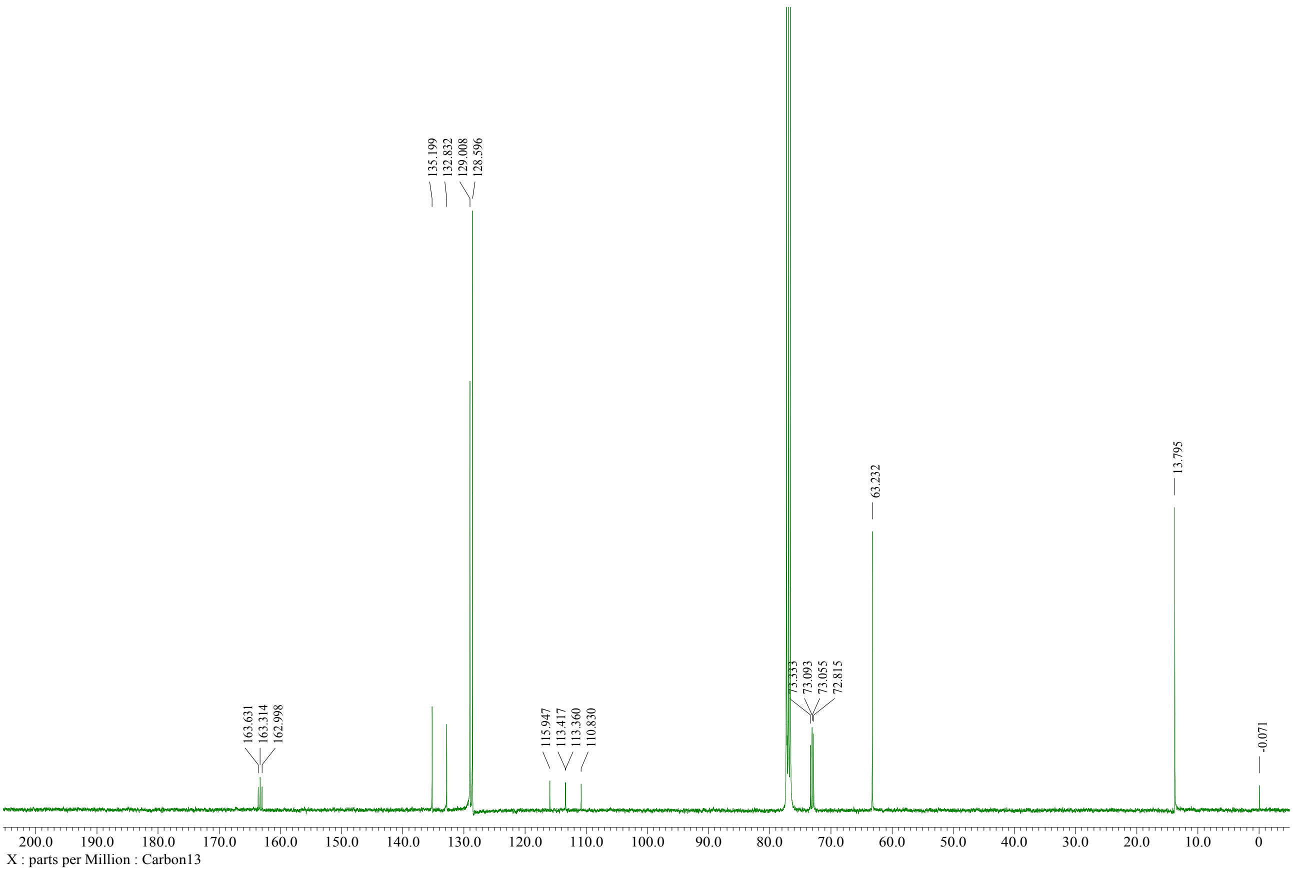

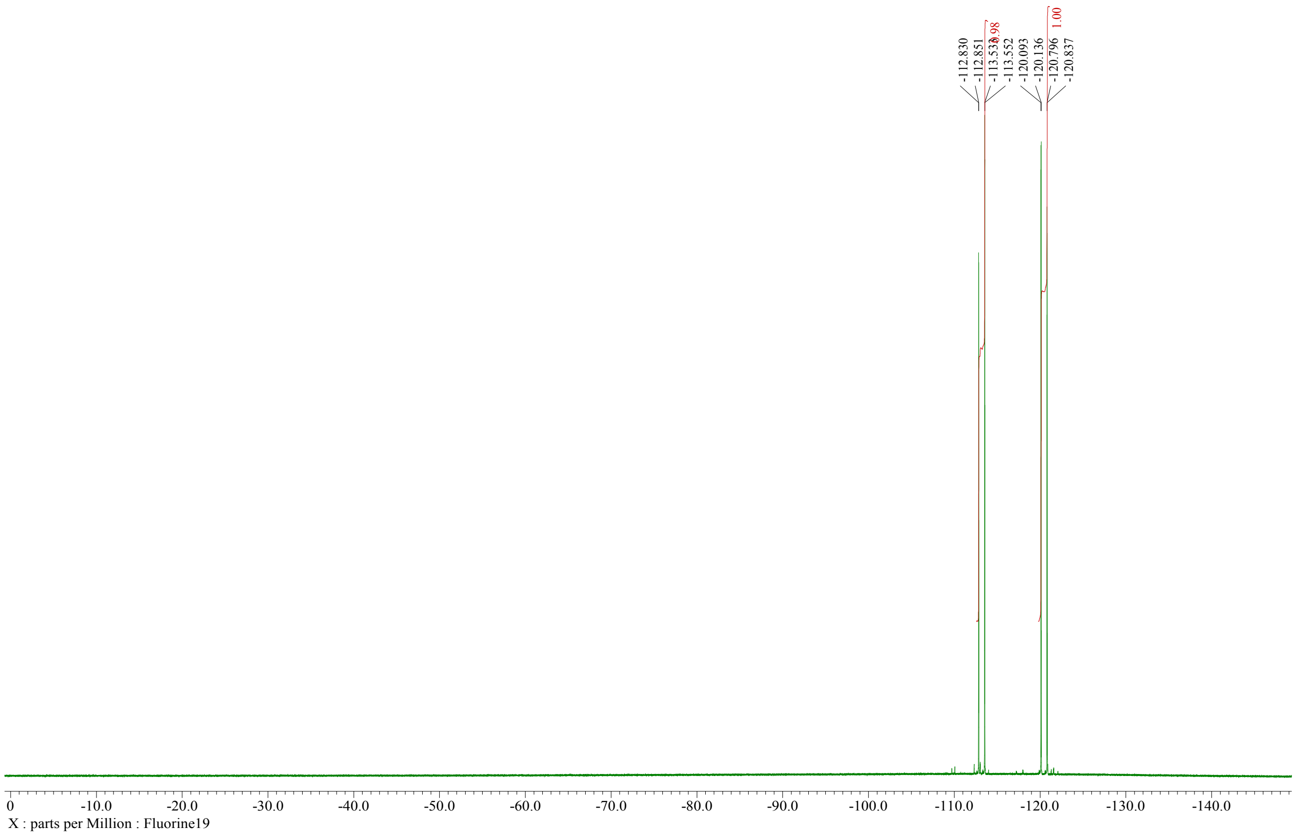

Ethyl 3-cyclohexyl-2,2-difluoro-3-hydroxypropanoate (4d)

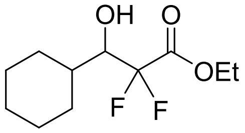

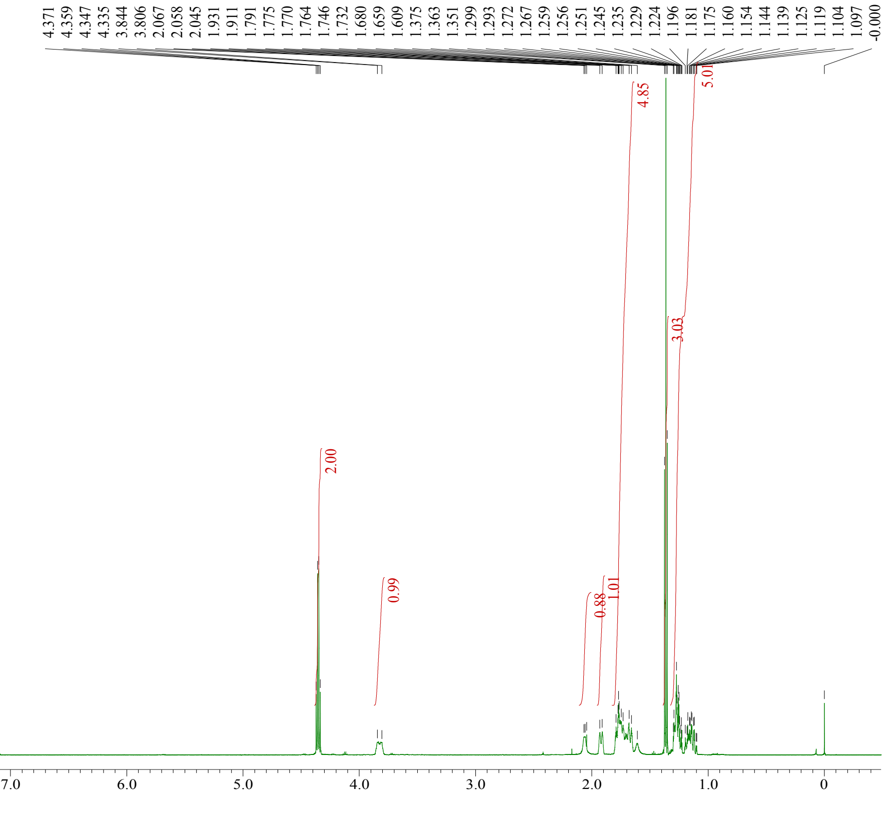

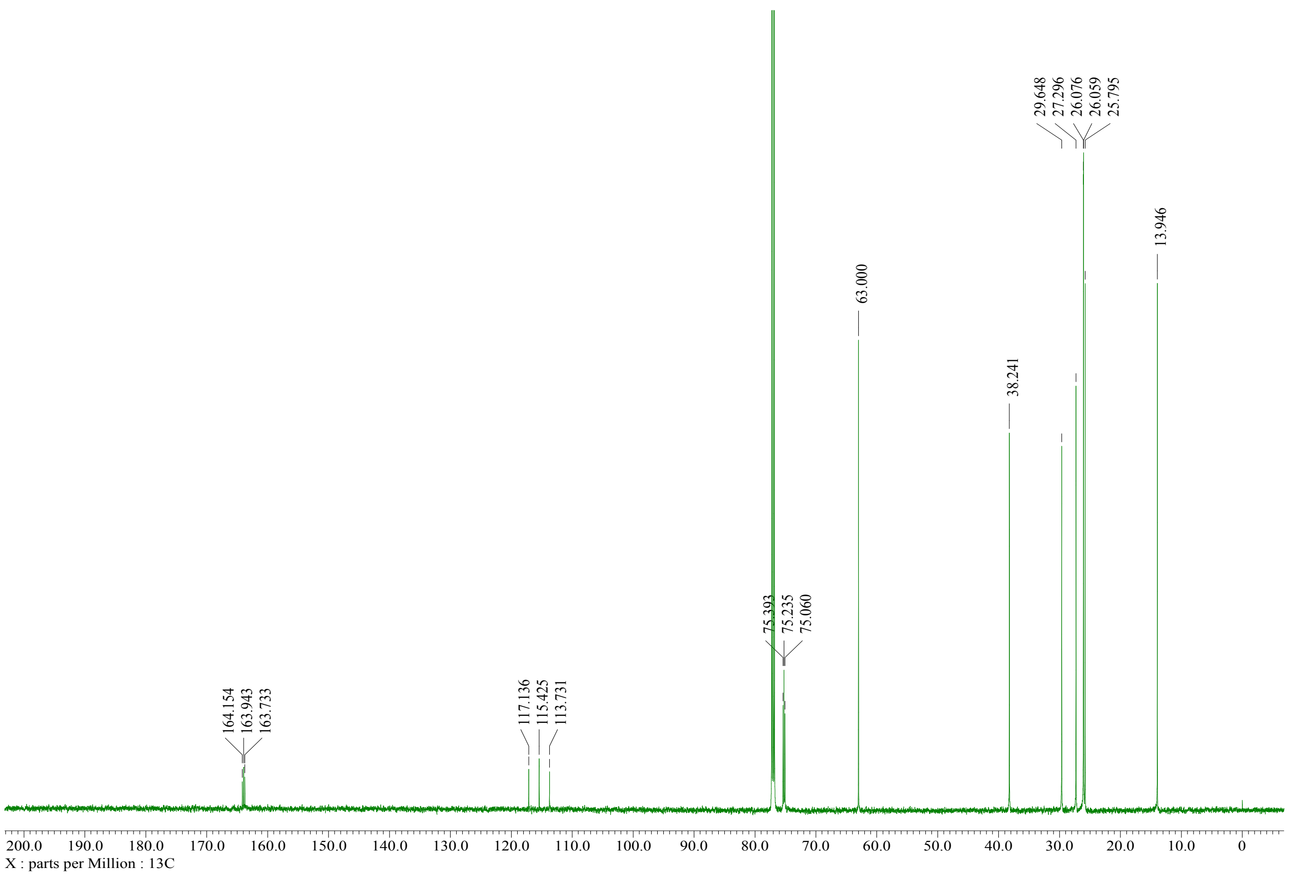

164.154
163.943
163.733
117.136
115.425
113.731
75.393
75.235
75.060
63.000
38.241
29.648
27.296
26.076
26.059
25.795
13.946
X : parts per Million : 13C

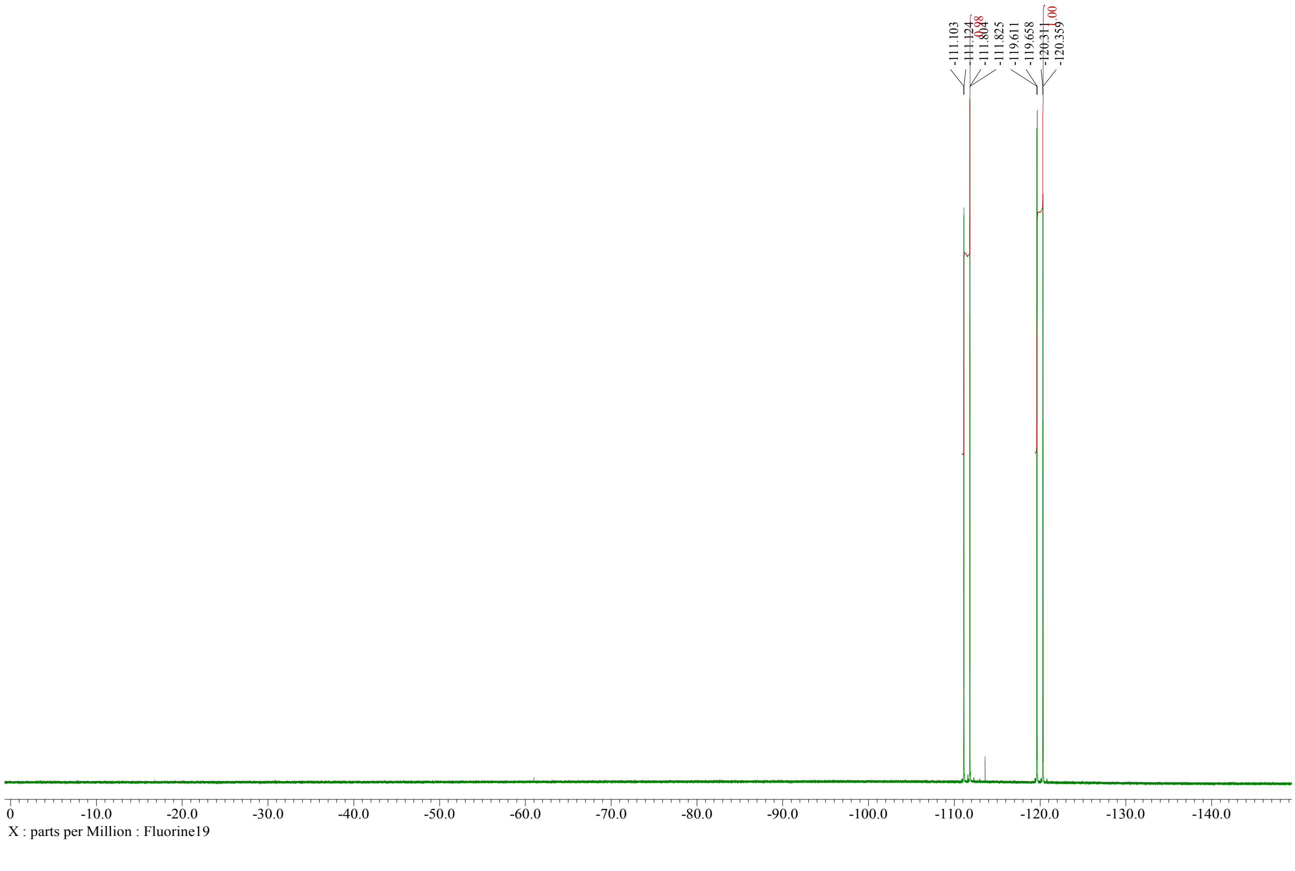

-111.103
-111.124
-111.804
-111.825
-119.611
-119.658
-120.311
-120.359
X : parts per Million : Fluorine19

Ethyl 2,2-difluoro-3-oxo-3-phenylpropanoate (5a)

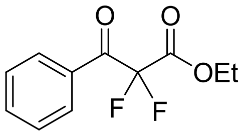

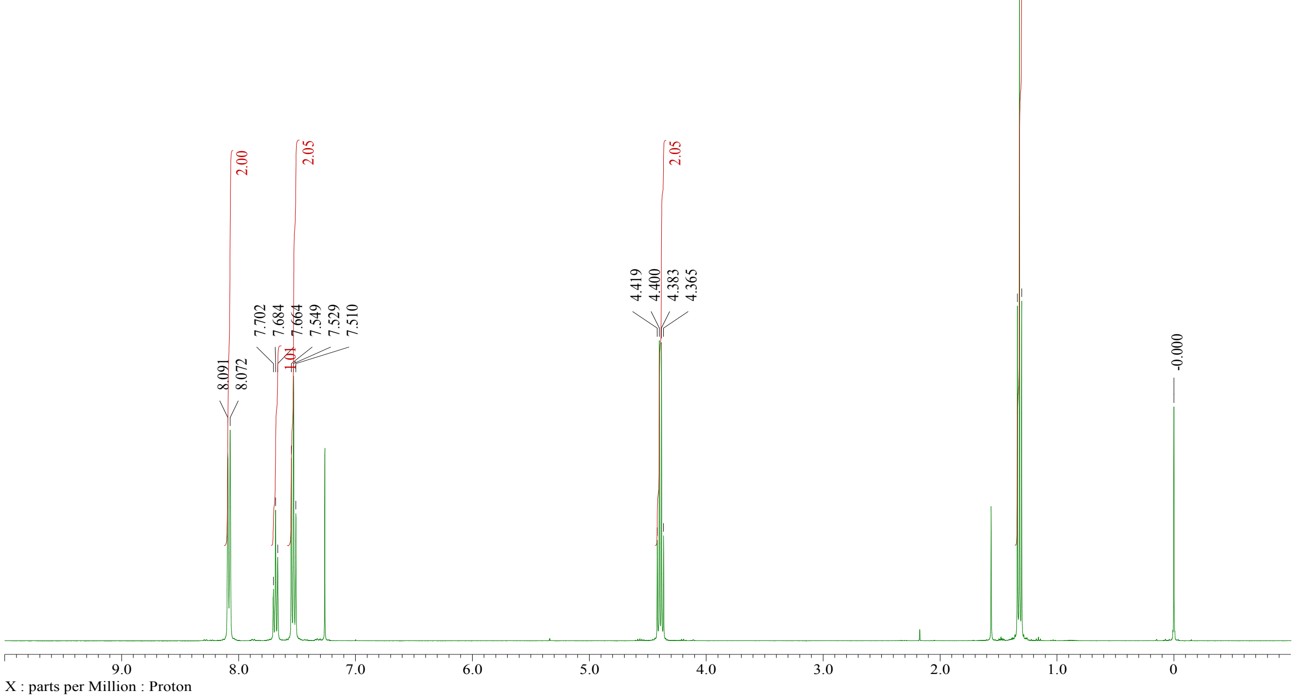

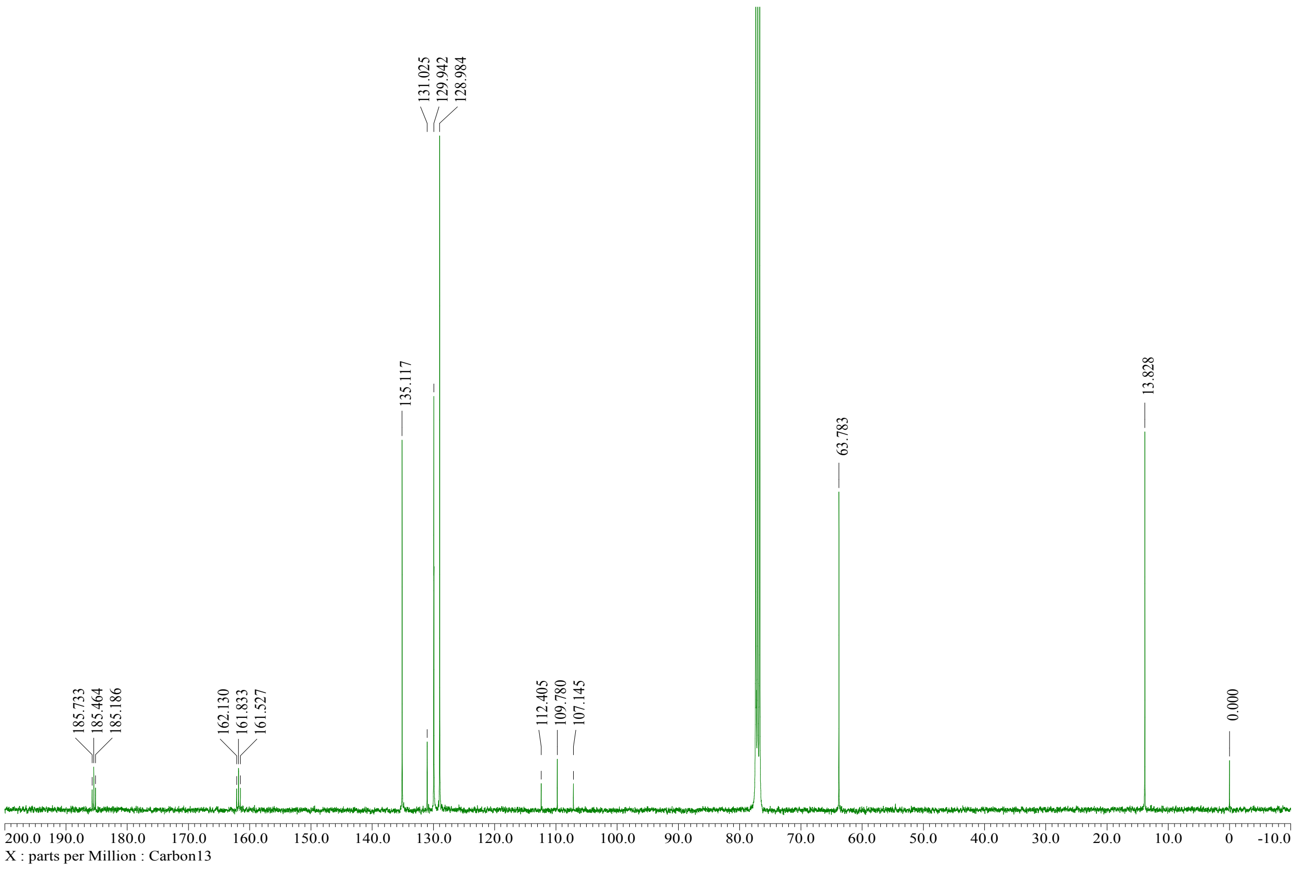

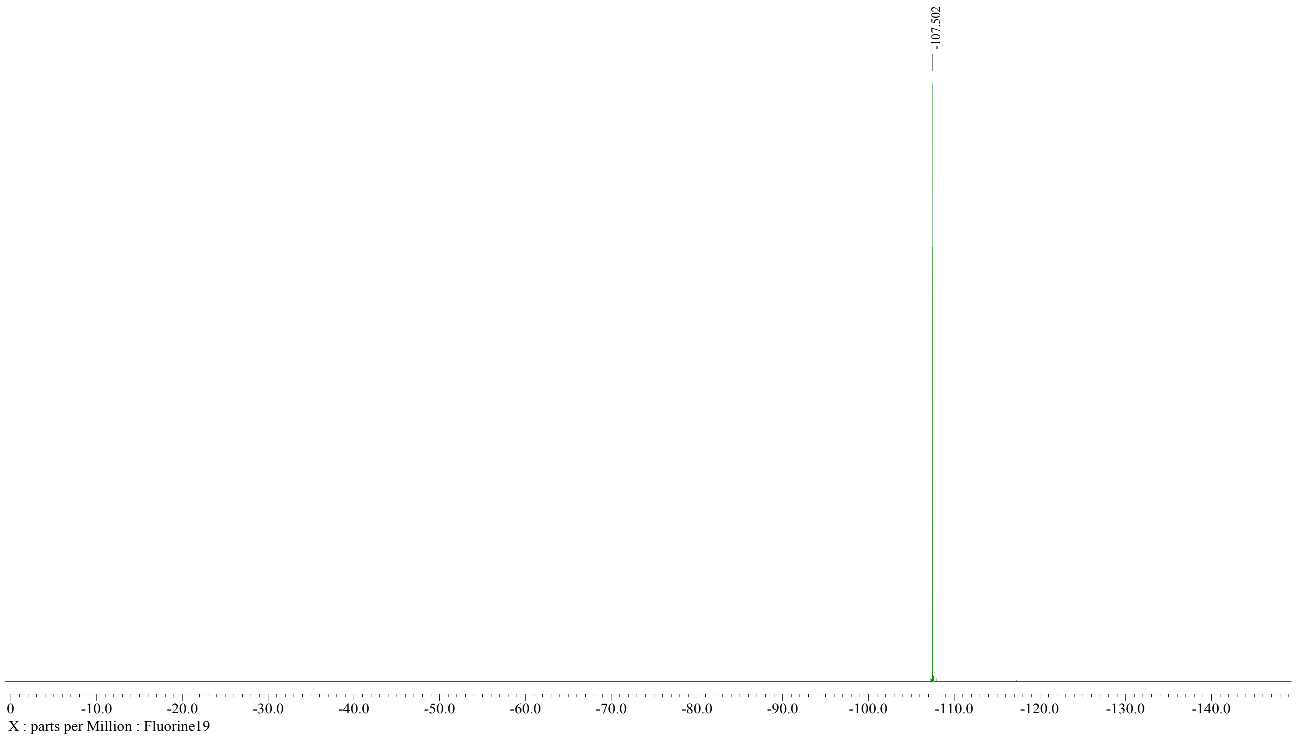

Ethyl 2,2-difluoro-3-(4-methoxyphenyl)-3-oxopropanoate (5b)

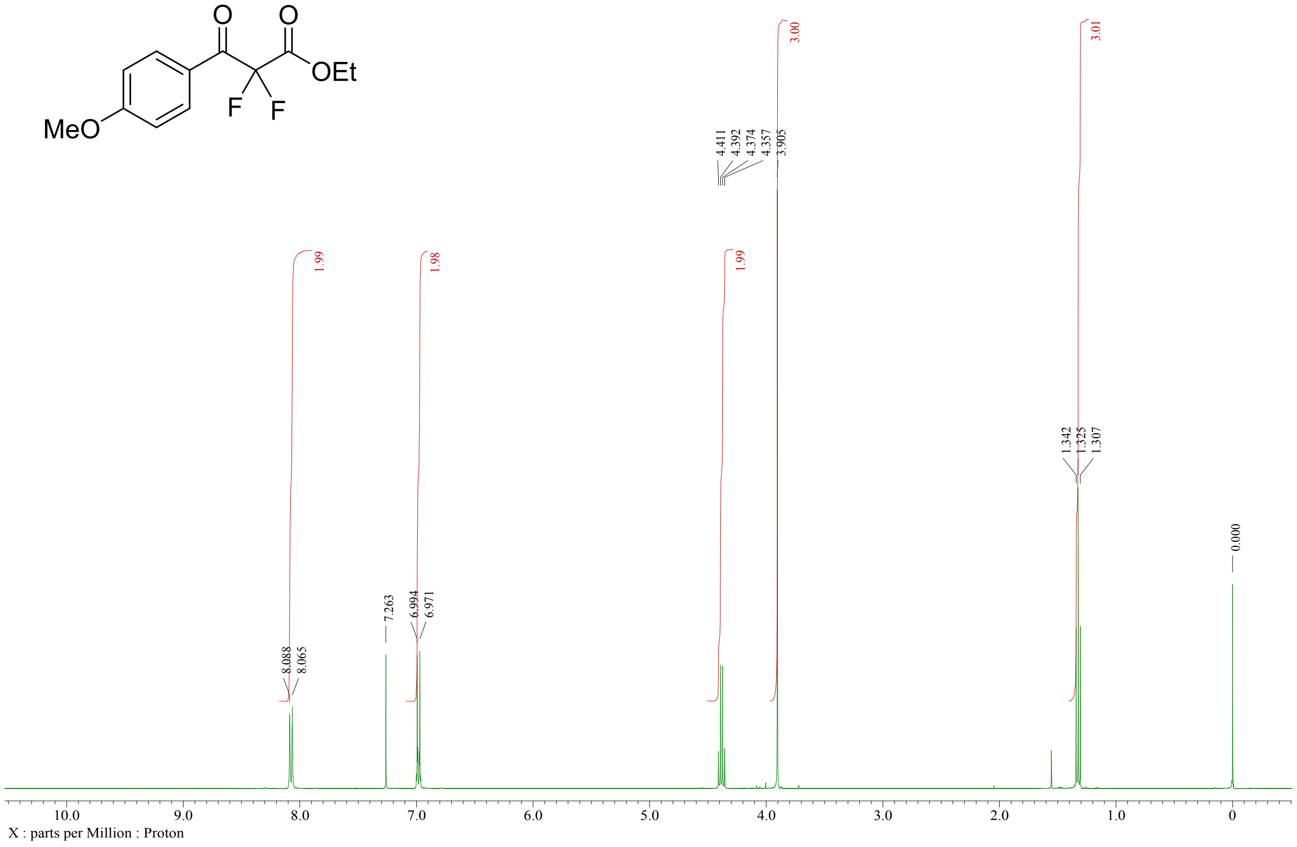

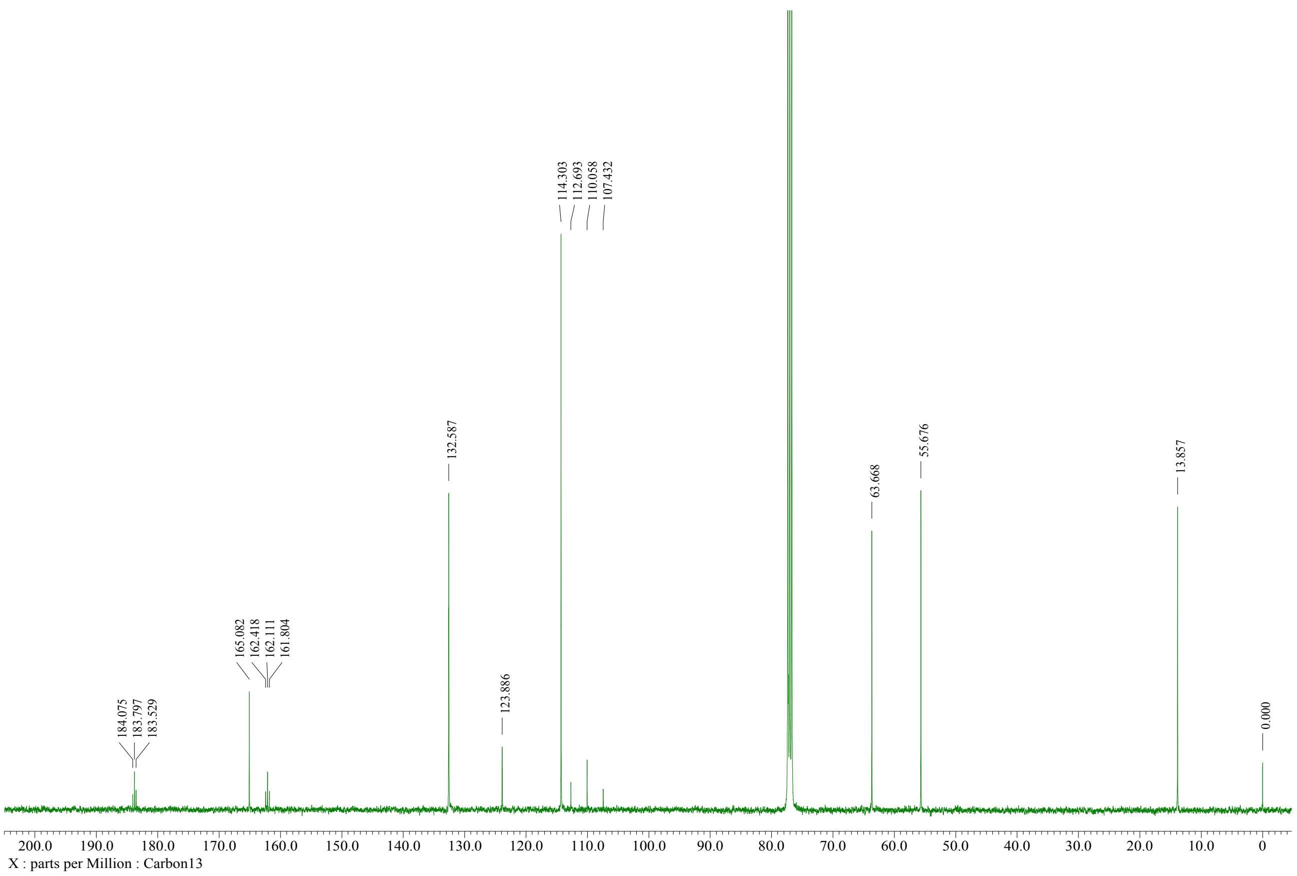
184.075
183.797
183.529
165.082
162.418
162.111
161.804
132.587
123.886
114.303
112.693
110.058
107.432
63.668
55.676
13.857
0.000
200.0 190.0 180.0 170.0 160.0 150.0 140.0 130.0 120.0 110.0 100.0 90.0 80.0 70.0 60.0 50.0 40.0 30.0 20.0 10.0 0
X : parts per Million : Carbon13

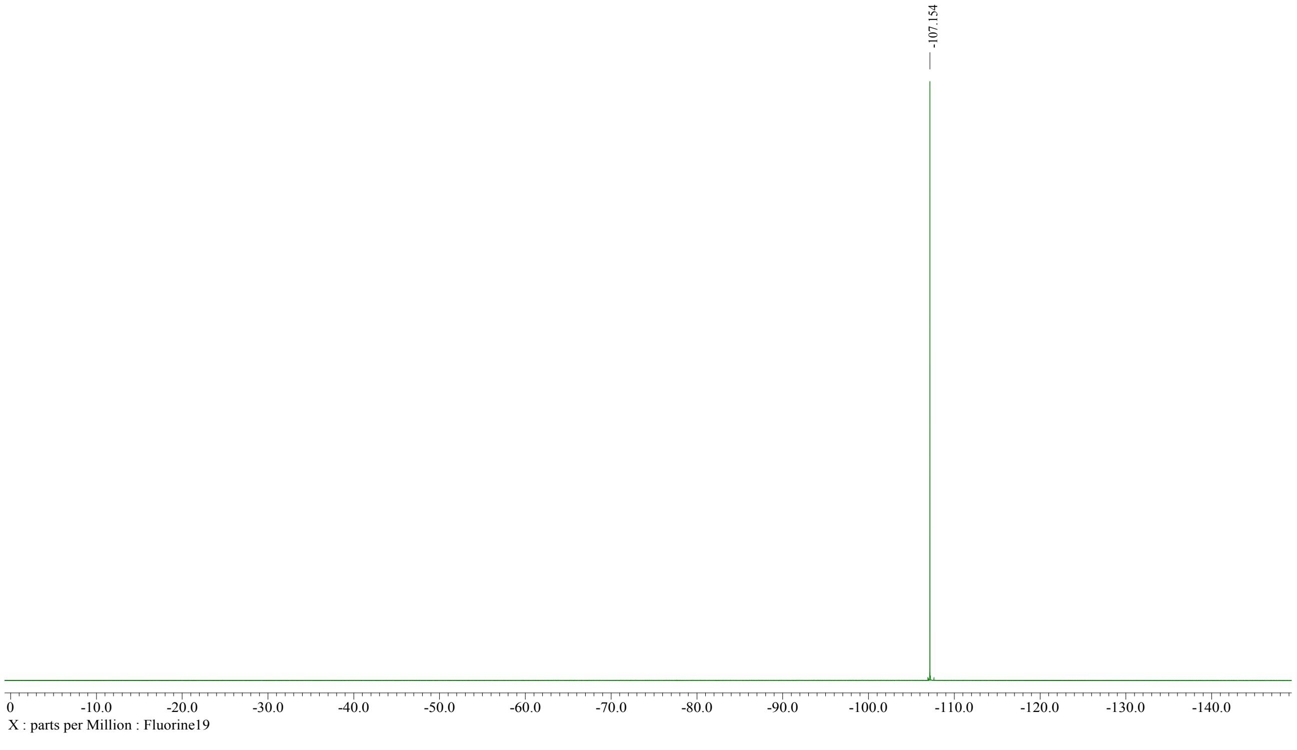
-107.154
0 -10.0 -20.0 -30.0 -40.0 -50.0 -60.0 -70.0 -80.0 -90.0 -100.0 -110.0 -120.0 -130.0 -140.0
X : parts per Million : Fluorine19

Ethyl 3-(4-chlorophenyl)-2,2-difluoro-3-oxopropanoate (5c).

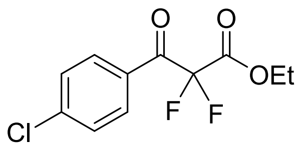

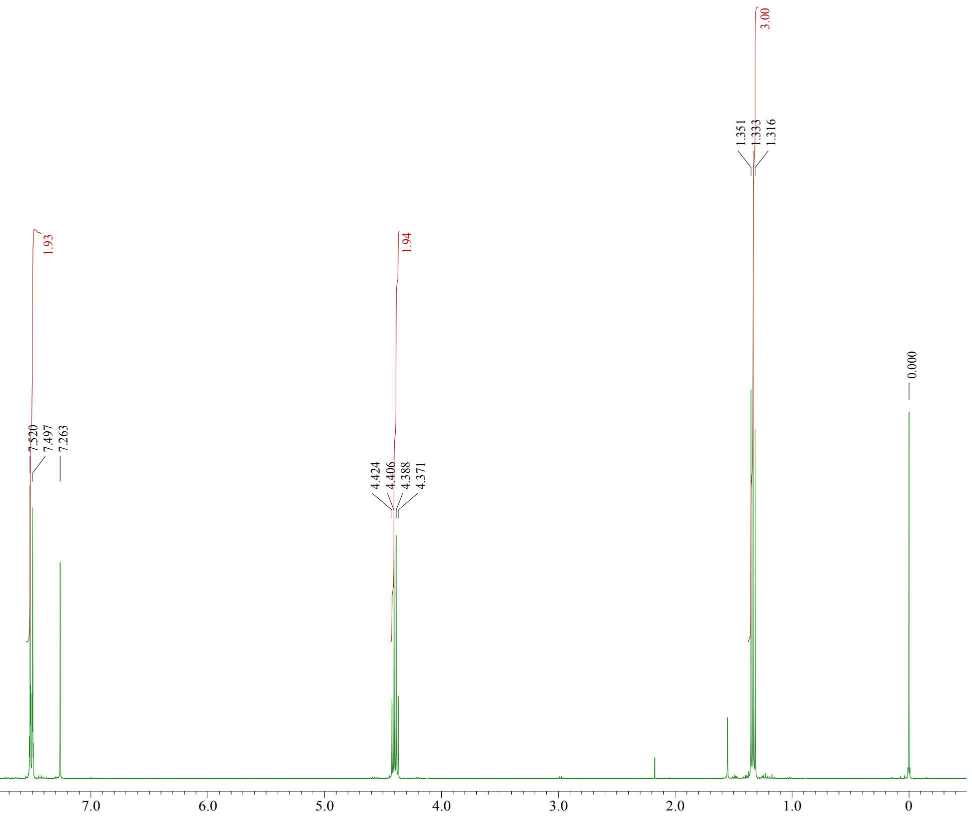

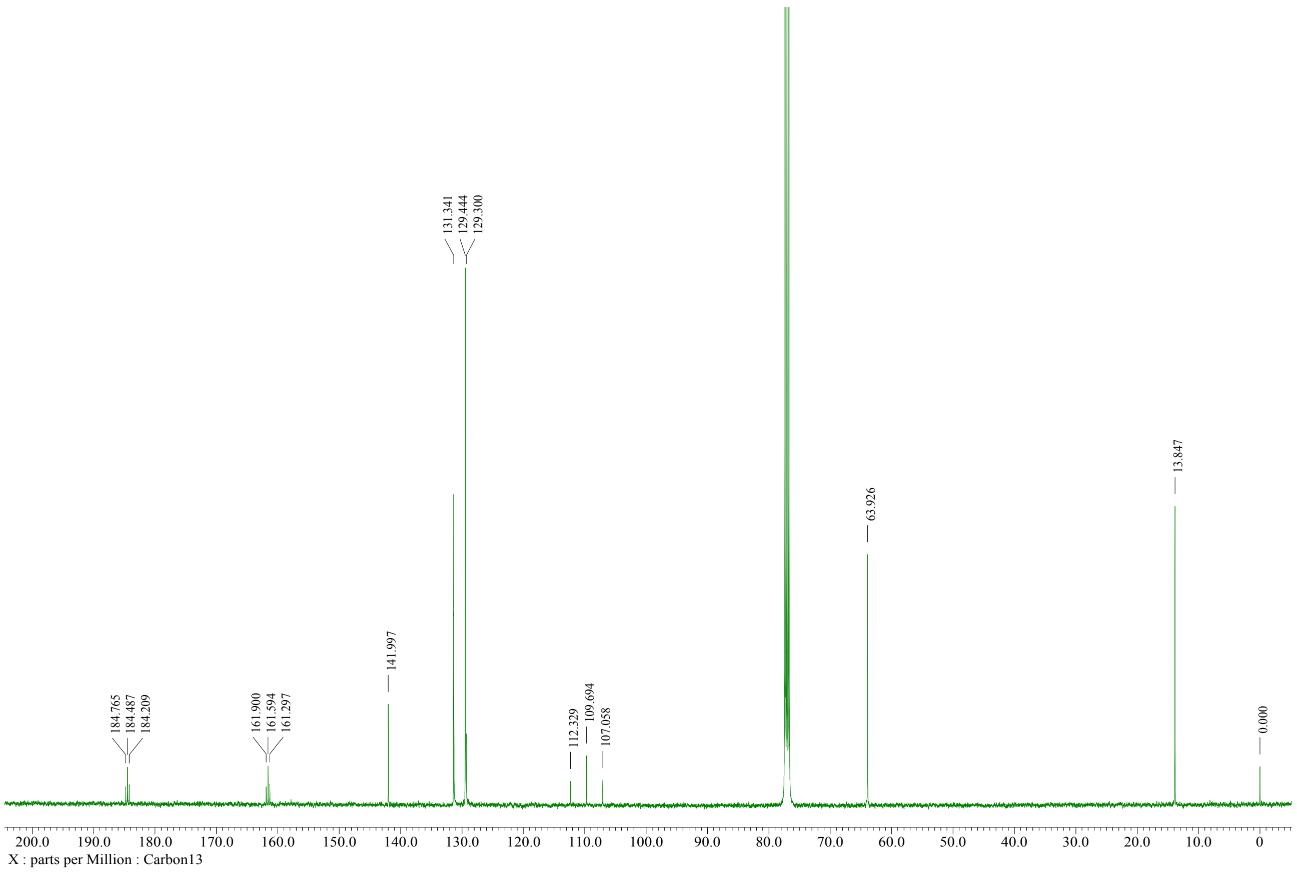

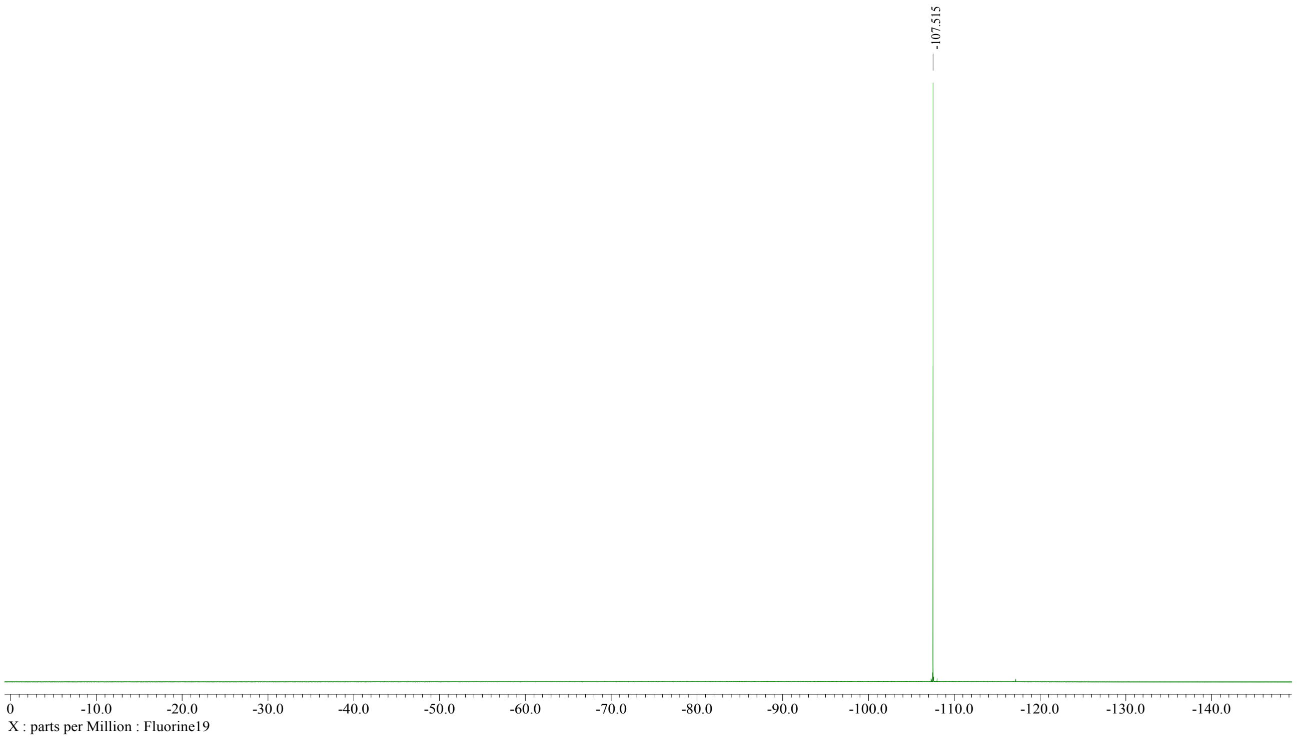

Ethyl 3-cyclohexyl-2,2-difluoro-3-oxopropanoate (5d)

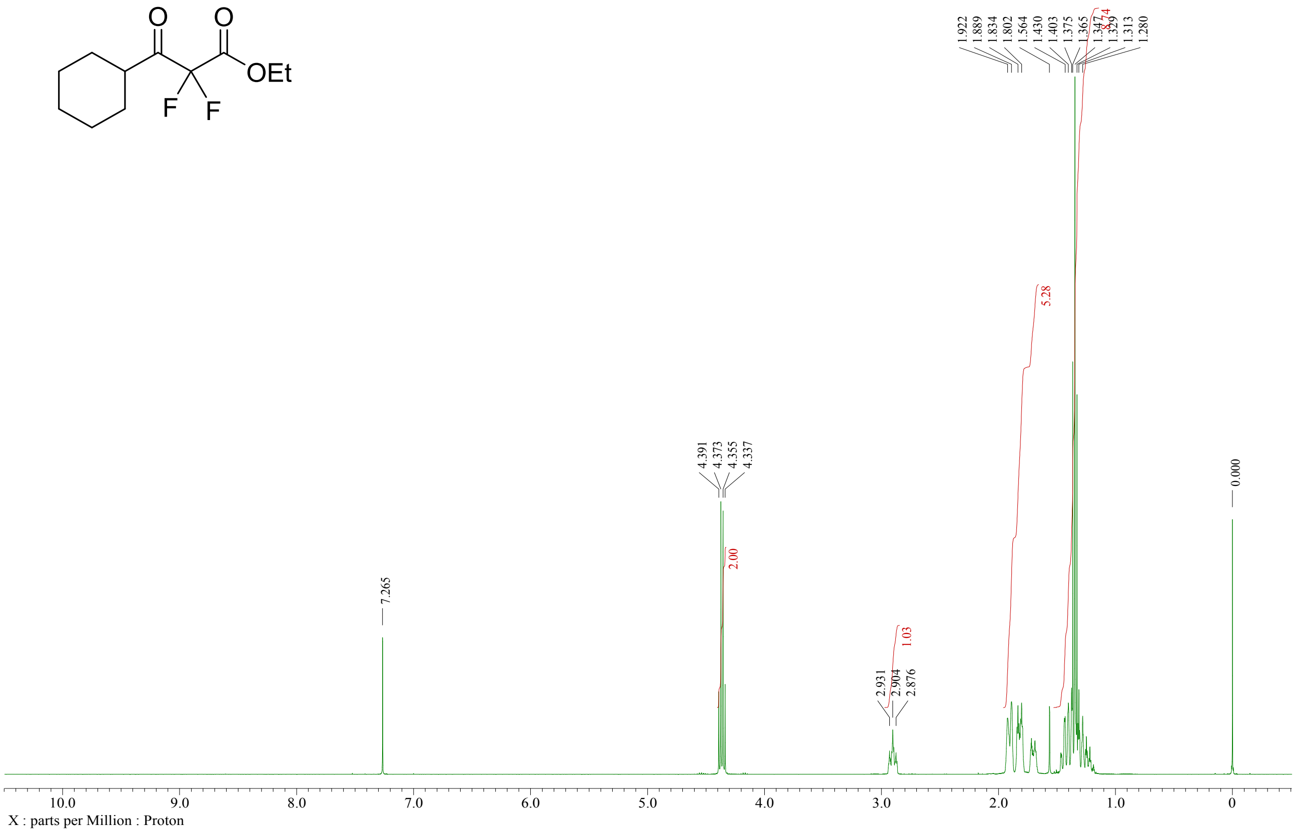

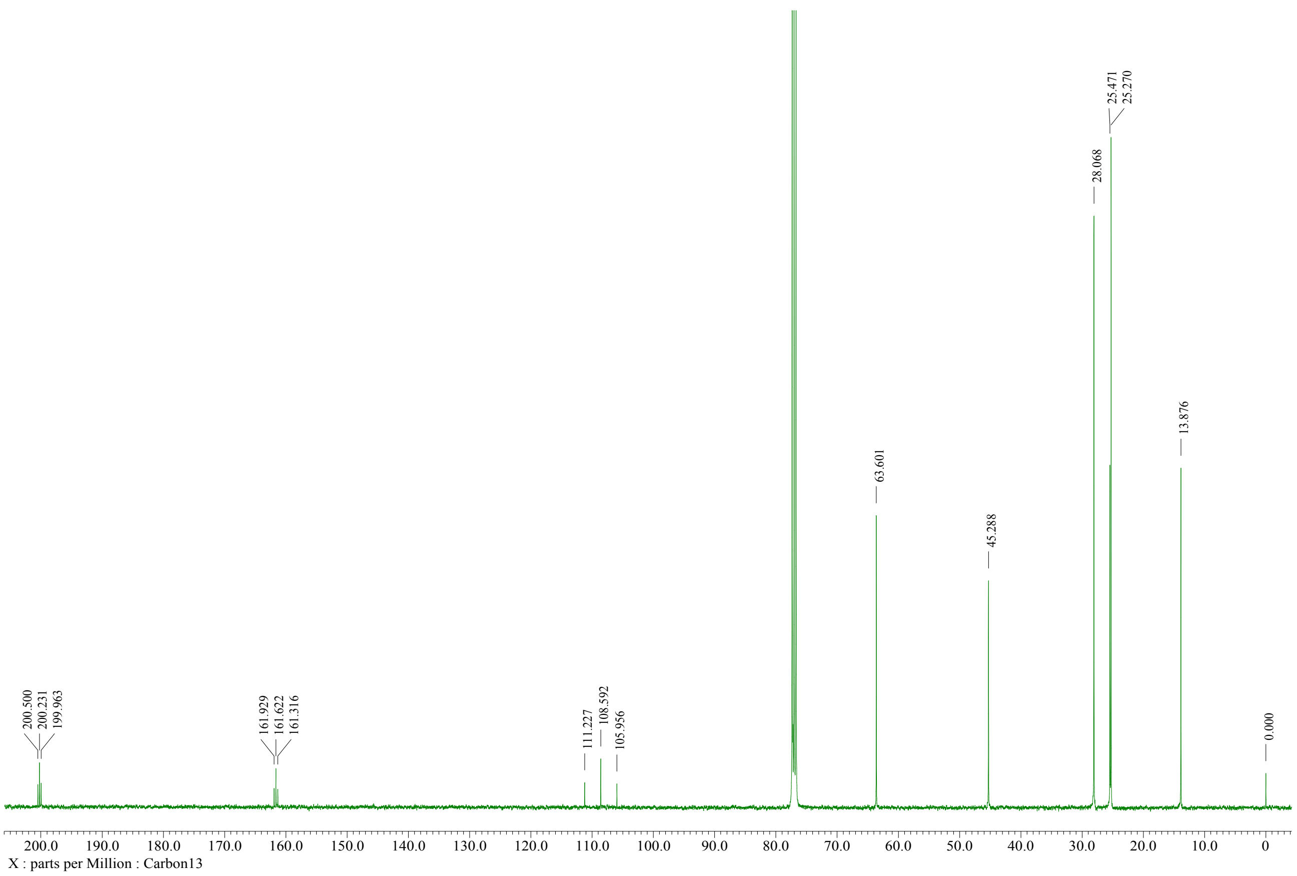
200.500
200.231
199.963
161.929
161.622
161.316
111.227
108.592
105.956
63.601
45.288
28.068
25.471
25.270
13.876
0.000
X : parts per Million : Carbon13

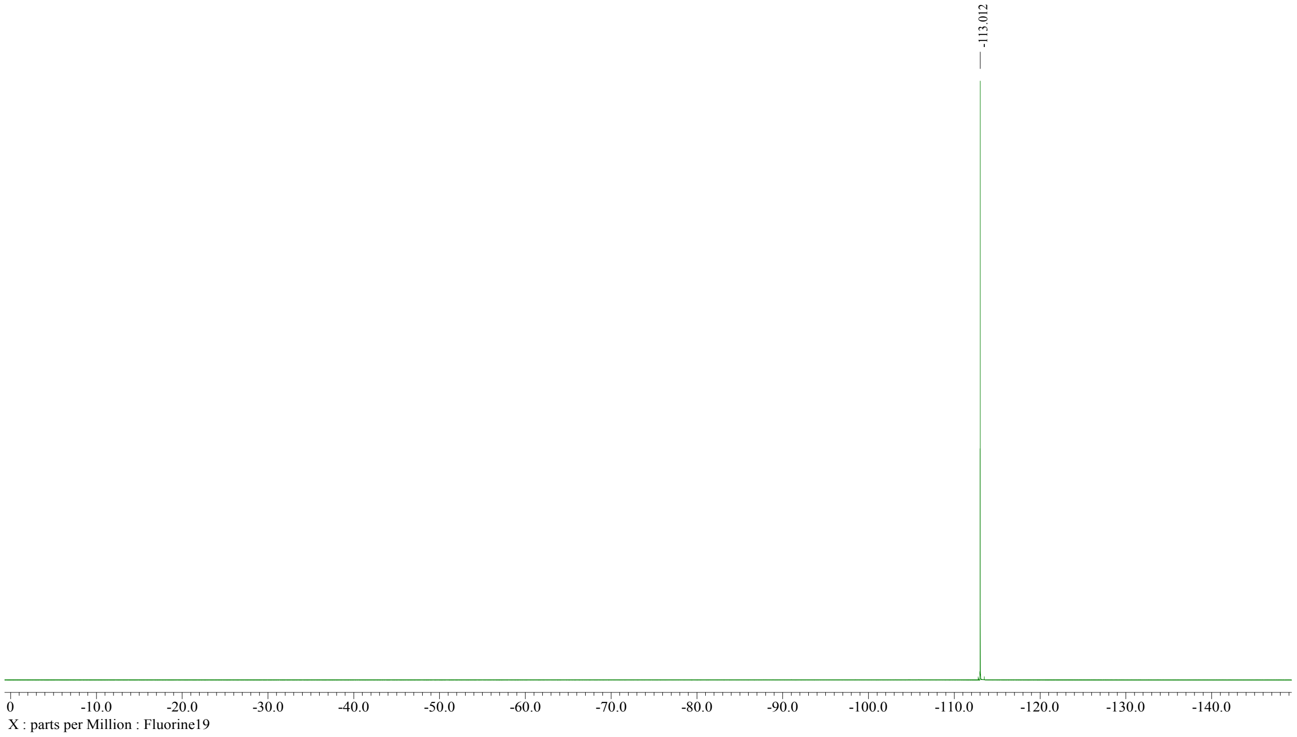
-113.012
X : parts per Million : Fluorine19

Potassium 2,2-difluoro-3-oxo-3-phenylpropanoate (2a)

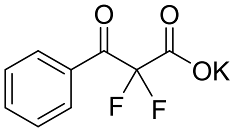

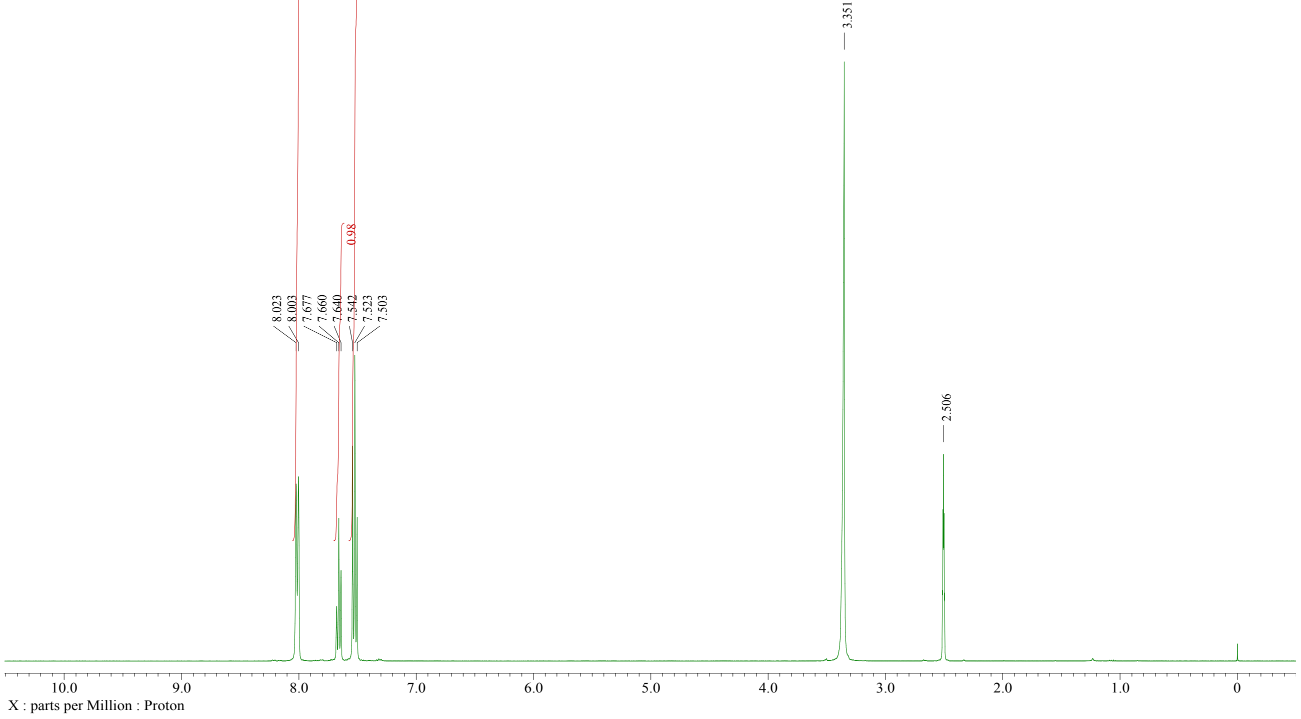

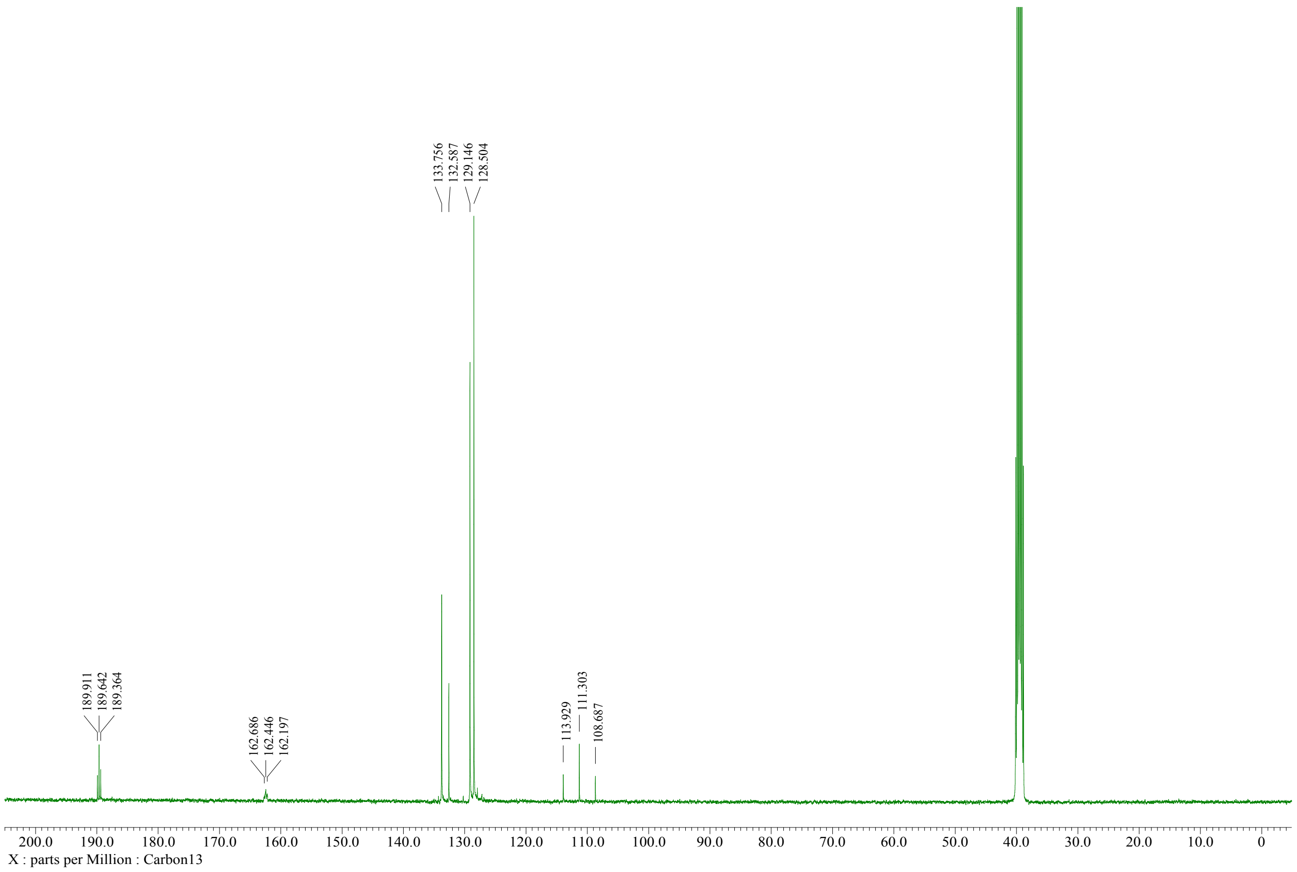

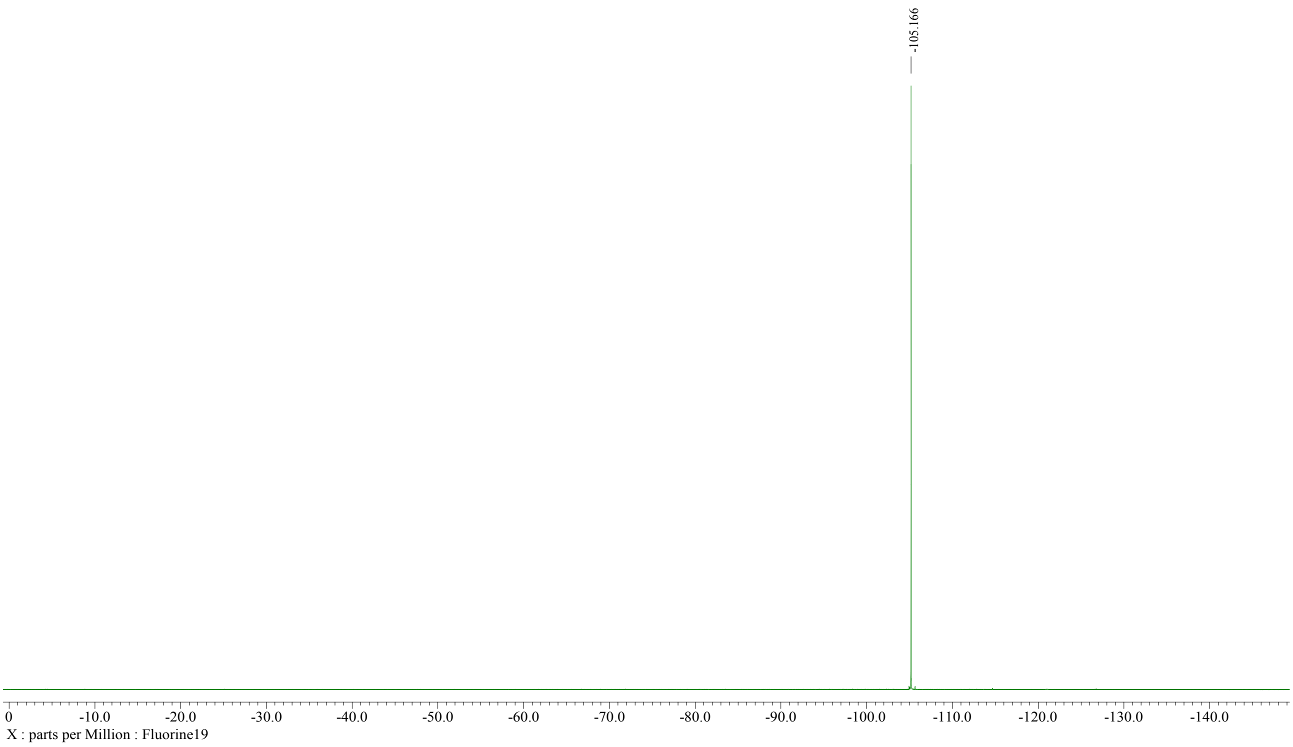

Potassium 2,2-difluoro-3-(4-methoxyphenyl)-3-oxopropanoate (2b)

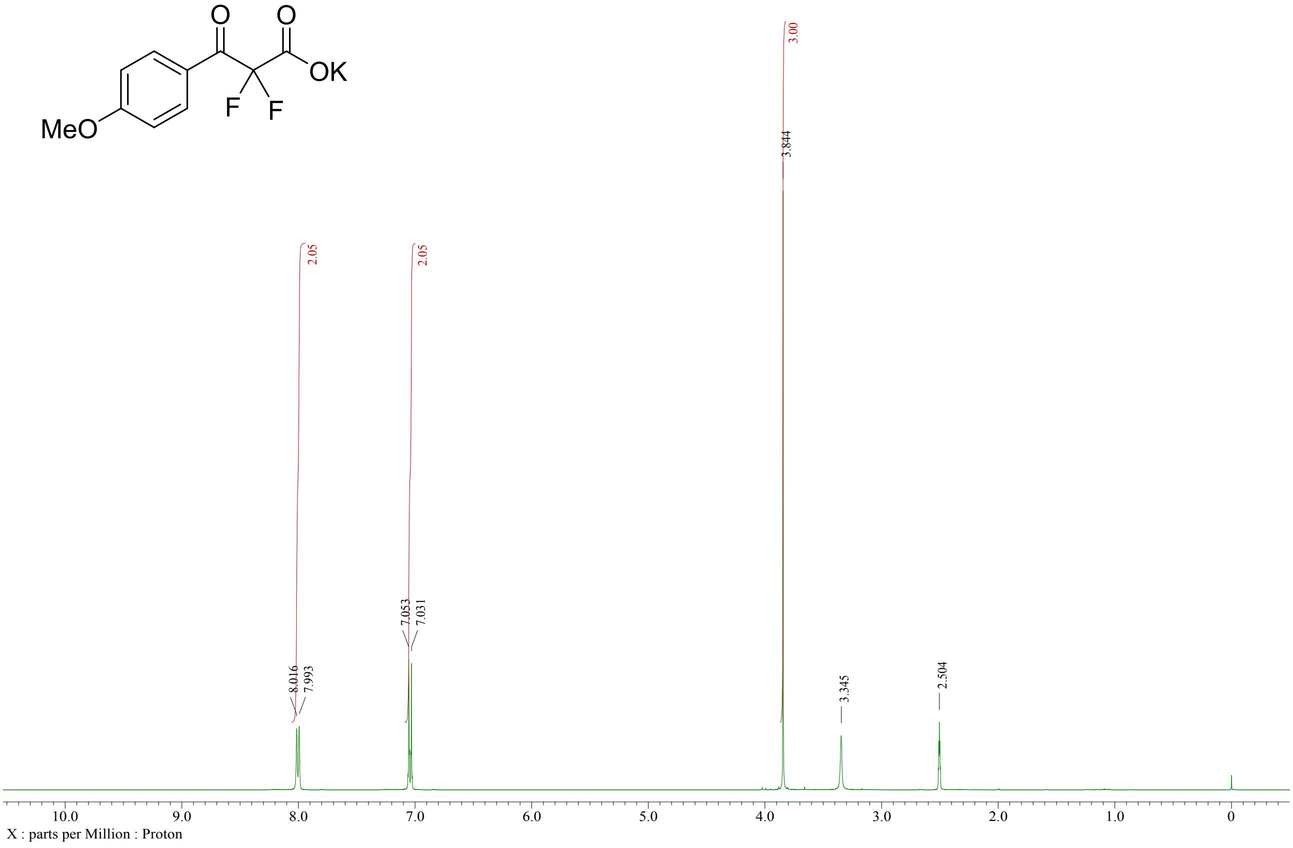

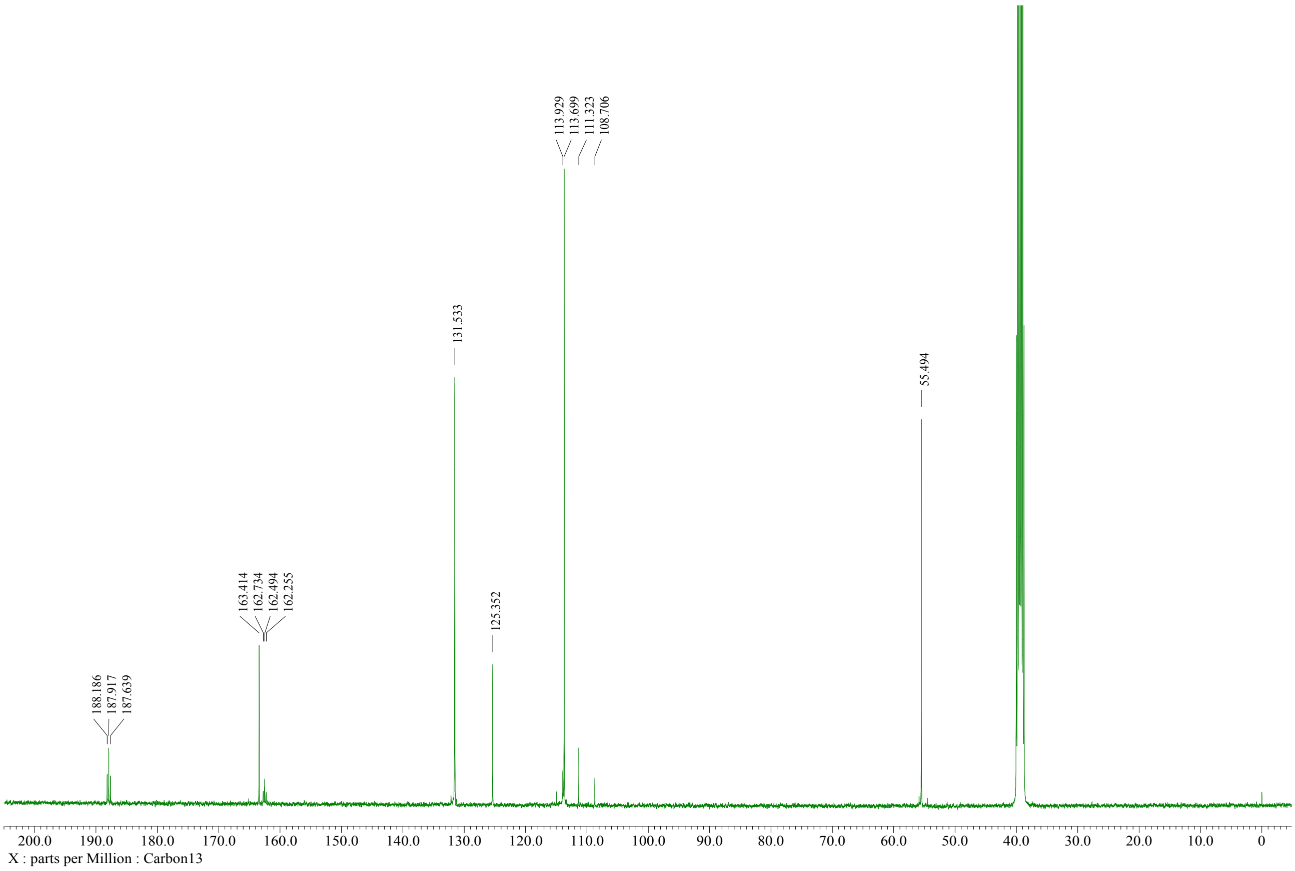
188.186
187.917
187.639
163.414
162.734
162.494
162.255
131.533
125.352
113.929
113.699
111.323
108.706
55.494
200.0 190.0 180.0 170.0 160.0 150.0 140.0 130.0 120.0 110.0 100.0 90.0 80.0 70.0 60.0 50.0 40.0 30.0 20.0 10.0 0
X : parts per Million : Carbon13

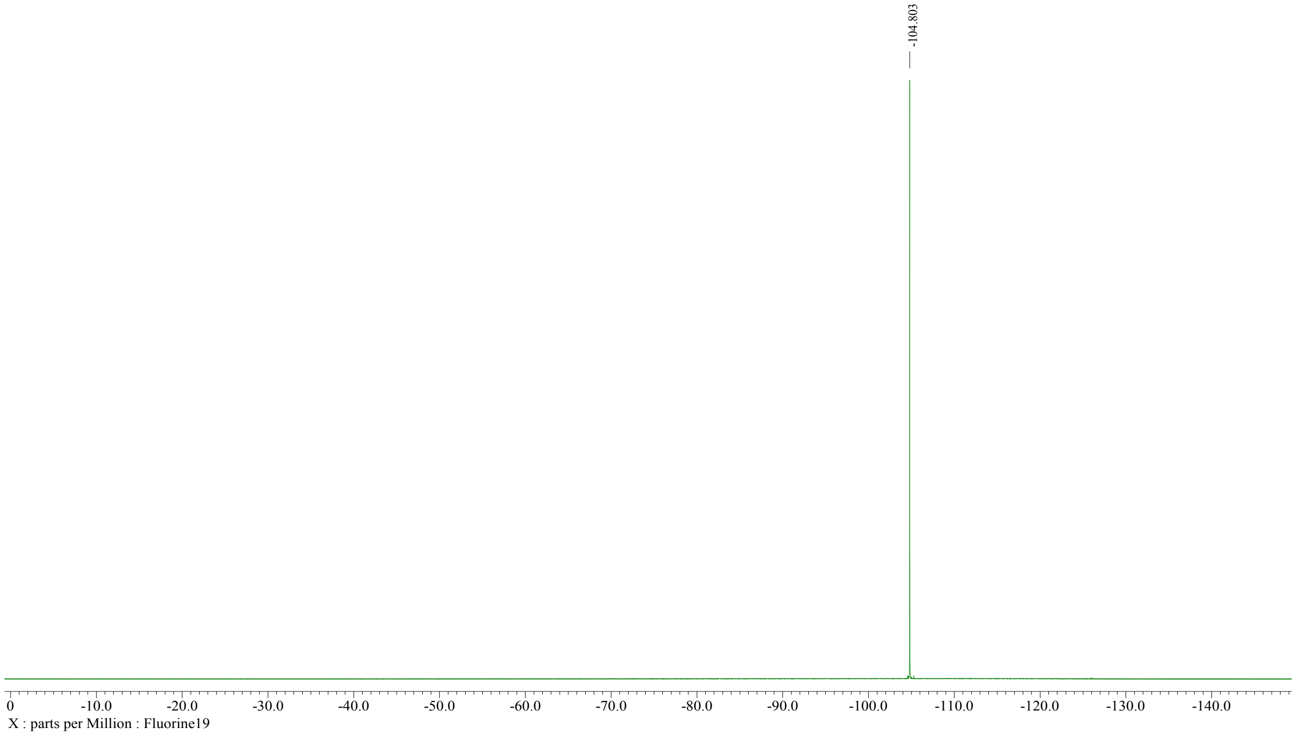
-104.803
0 -10.0 -20.0 -30.0 -40.0 -50.0 -60.0 -70.0 -80.0 -90.0 -100.0 -110.0 -120.0 -130.0 -140.0
X : parts per Million : Fluorine19

Potassium 3-(4-chlorophenyl)-2,2-difluoro-3-oxopropanoate (2c)

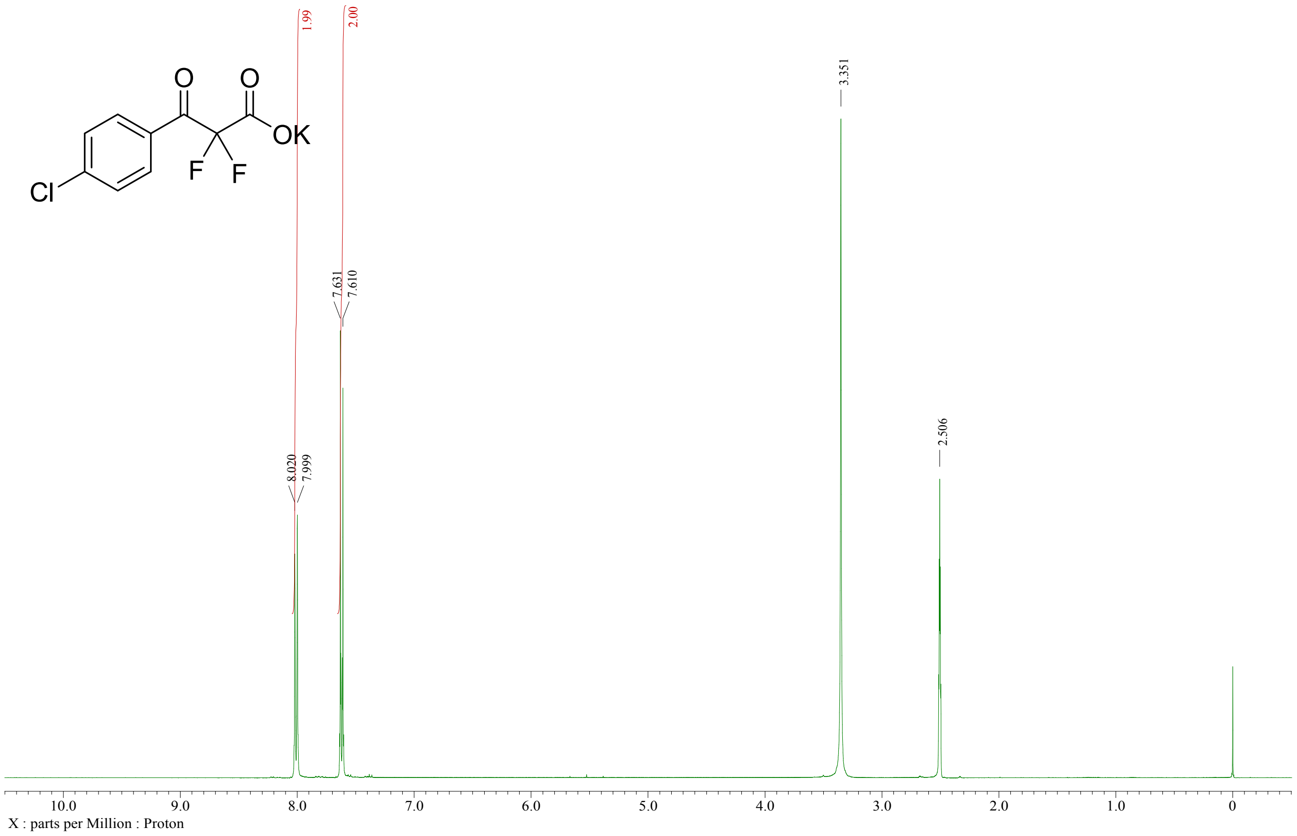

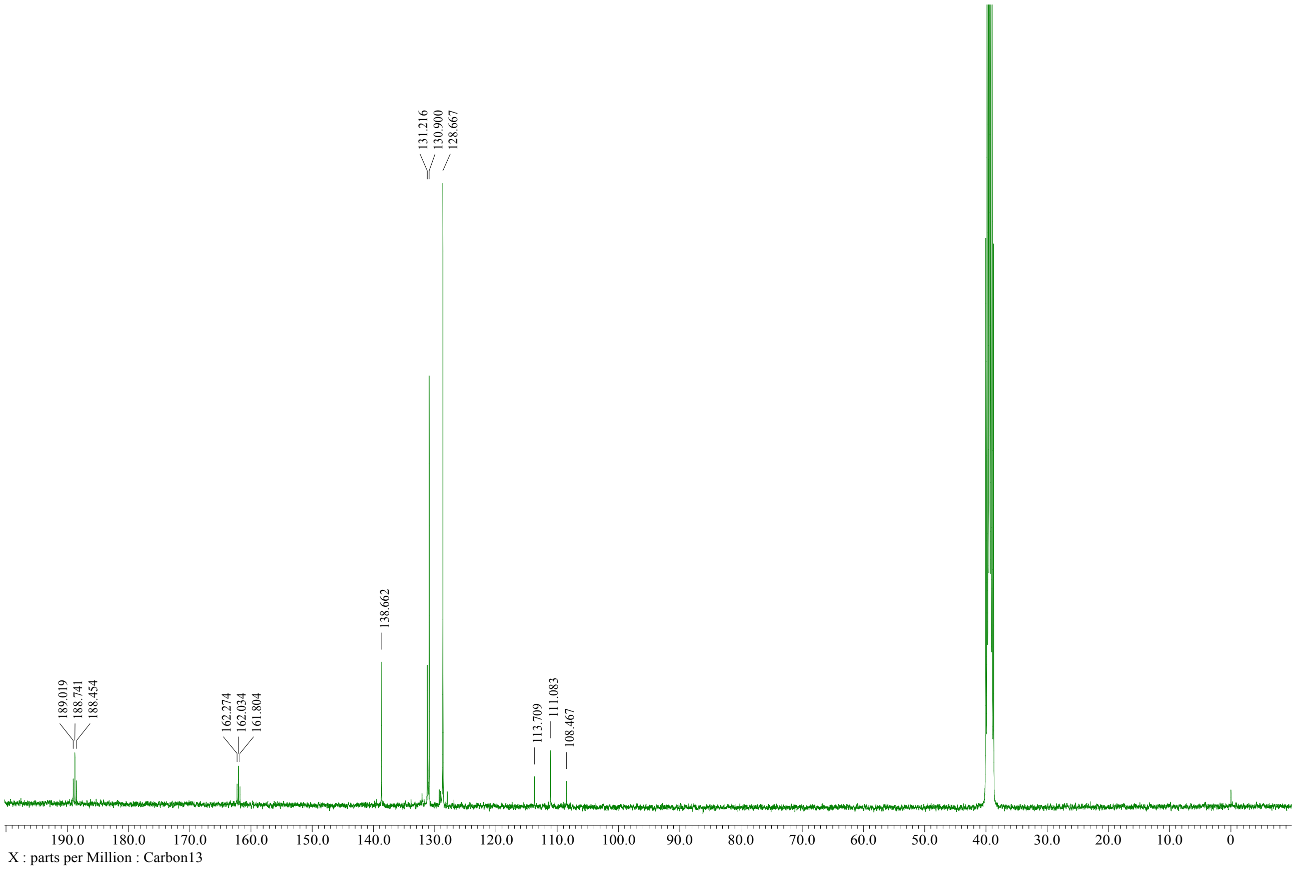

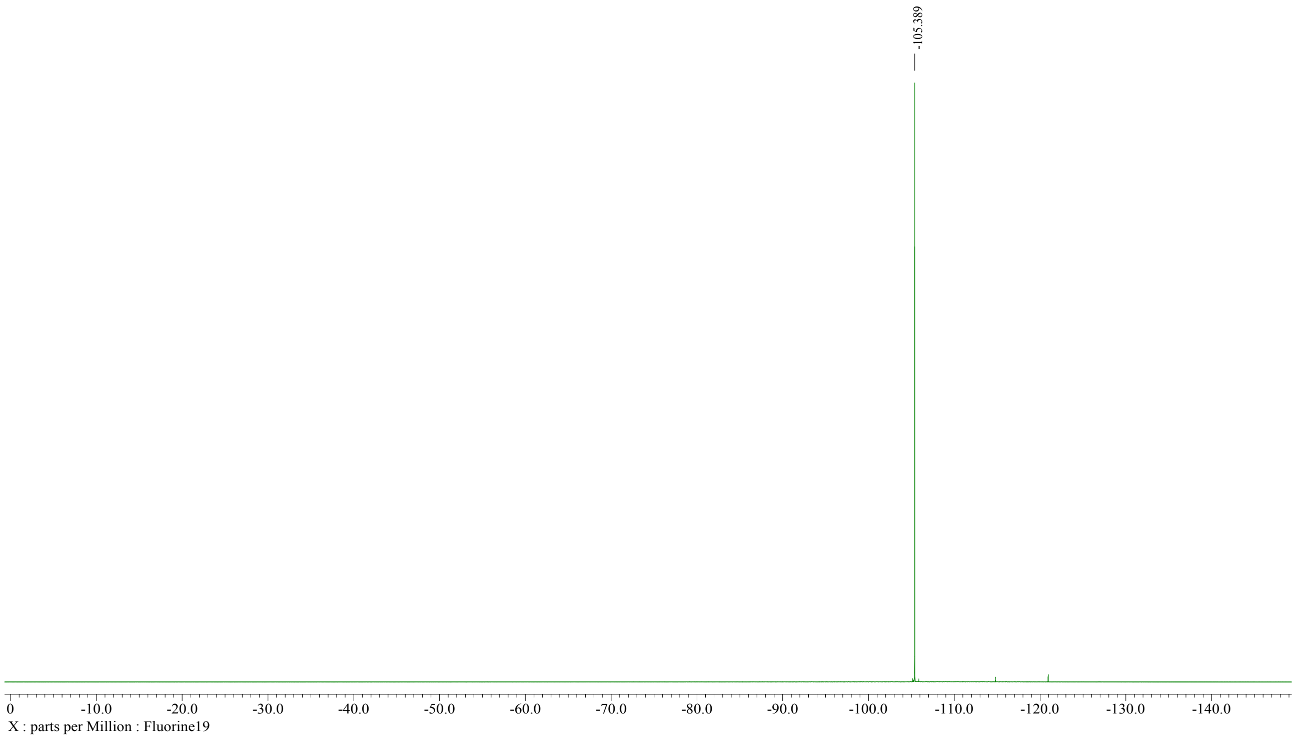

Potassium 3-cyclohexyl-2,2-difluoro-3-oxopropanoate (2d)

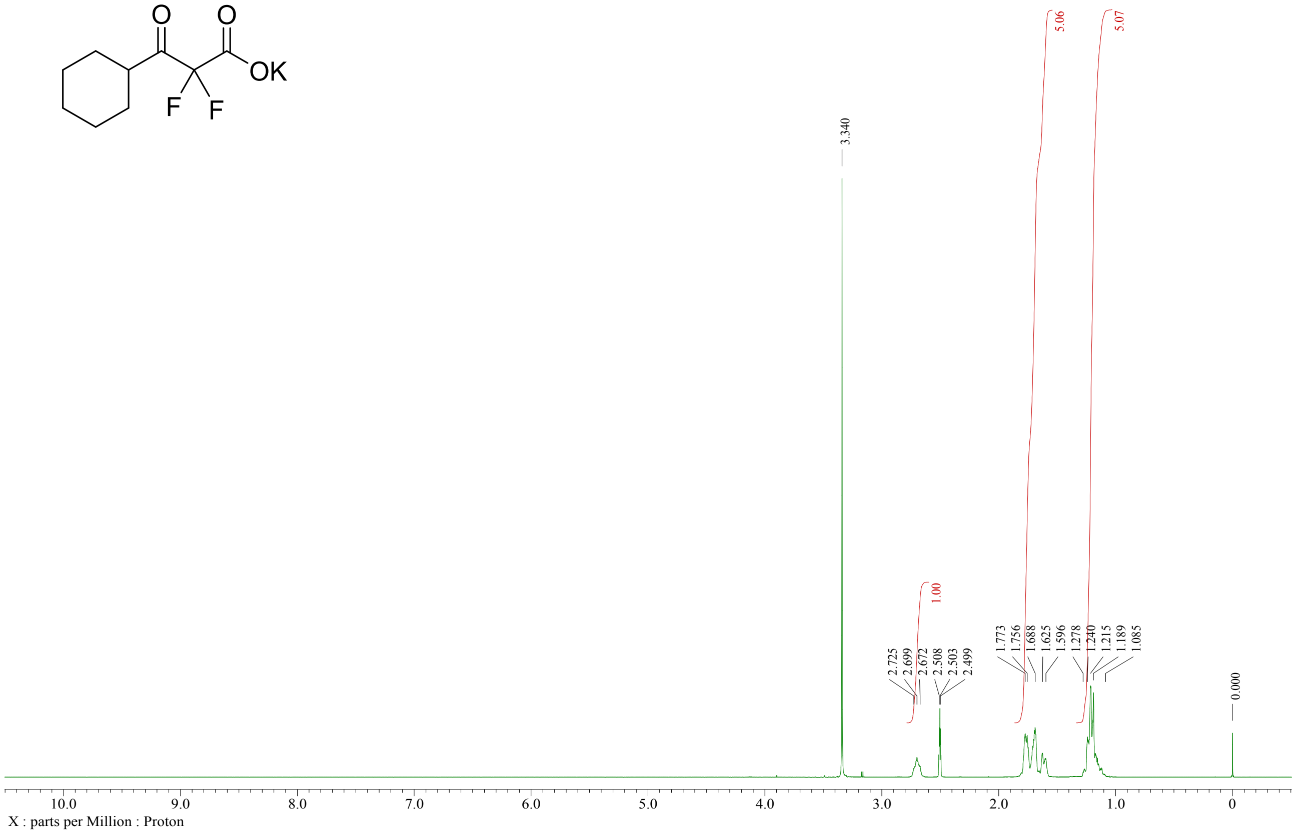

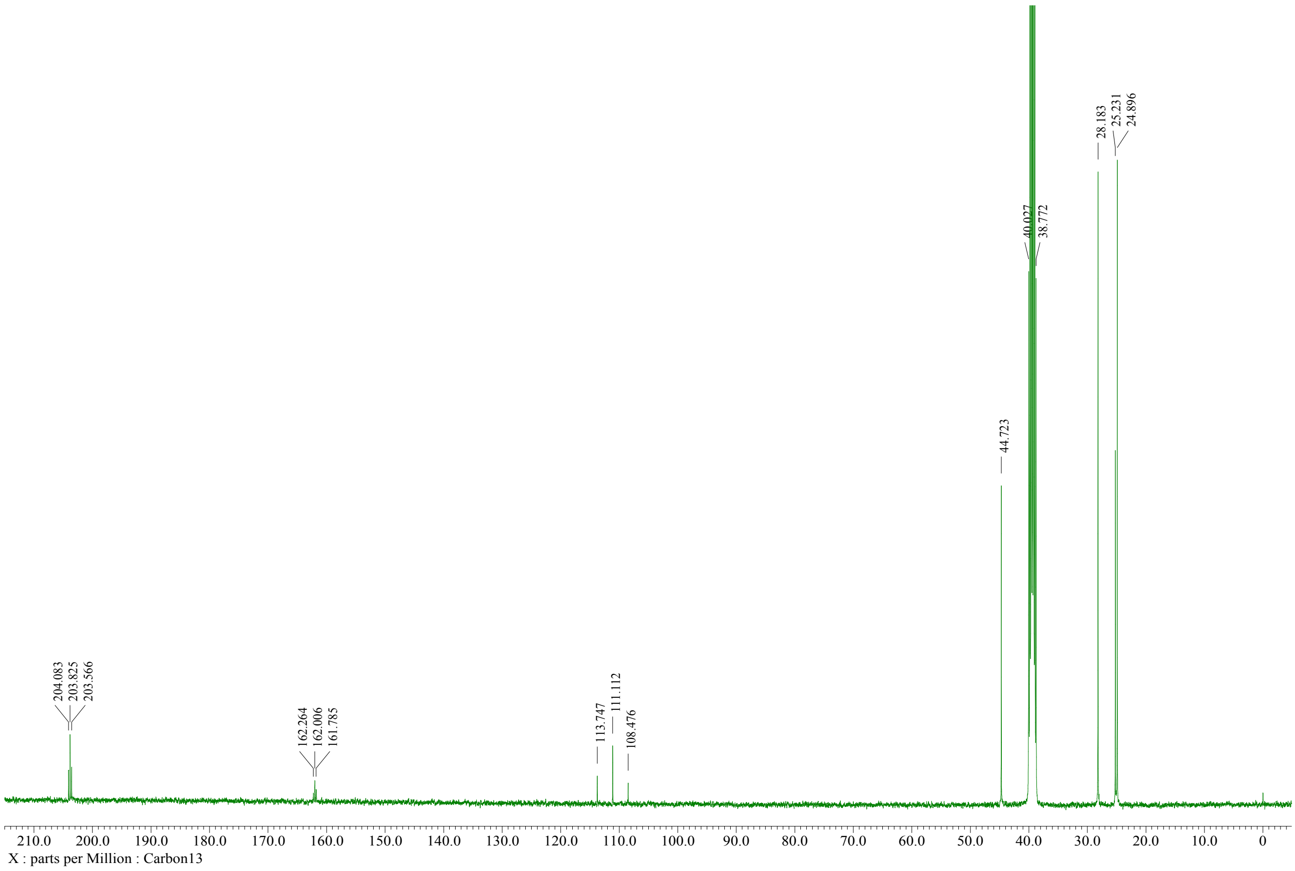
204.083
203.825
203.566
162.264
162.006
161.785
113.747
111.112
108.476
44.723
40.027
38.772
28.183
25.231
24.896
210.0 200.0 190.0 180.0 170.0 160.0 150.0 140.0 130.0 120.0 110.0 100.0 90.0 80.0 70.0 60.0 50.0 40.0 30.0 20.0 10.0 0
X : parts per Million : Carbon13

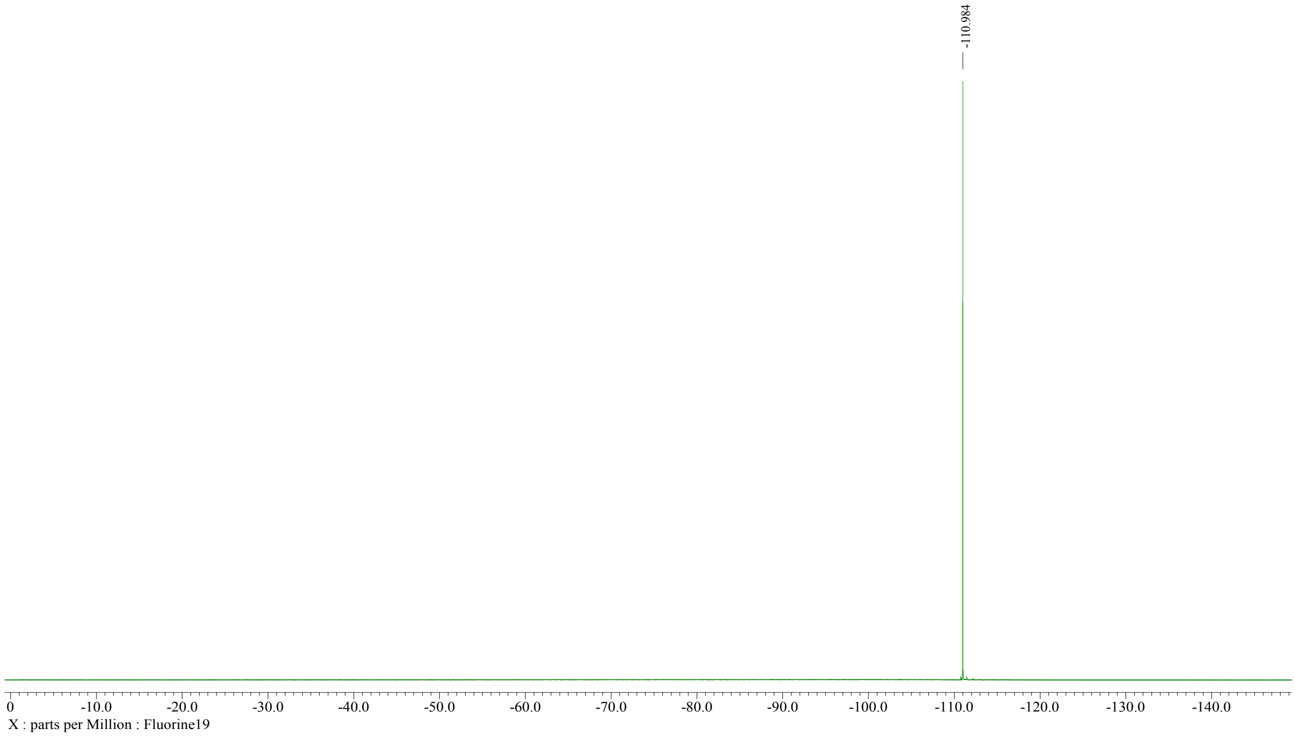
-110.984
0 -10.0 -20.0 -30.0 -40.0 -50.0 -60.0 -70.0 -80.0 -90.0 -100.0 -110.0 -120.0 -130.0 -140.0
X : parts per Million : Fluorine19

2,2-Difluoro-3-hydroxy-1,3-diphenylpropan-1-one (6aa)

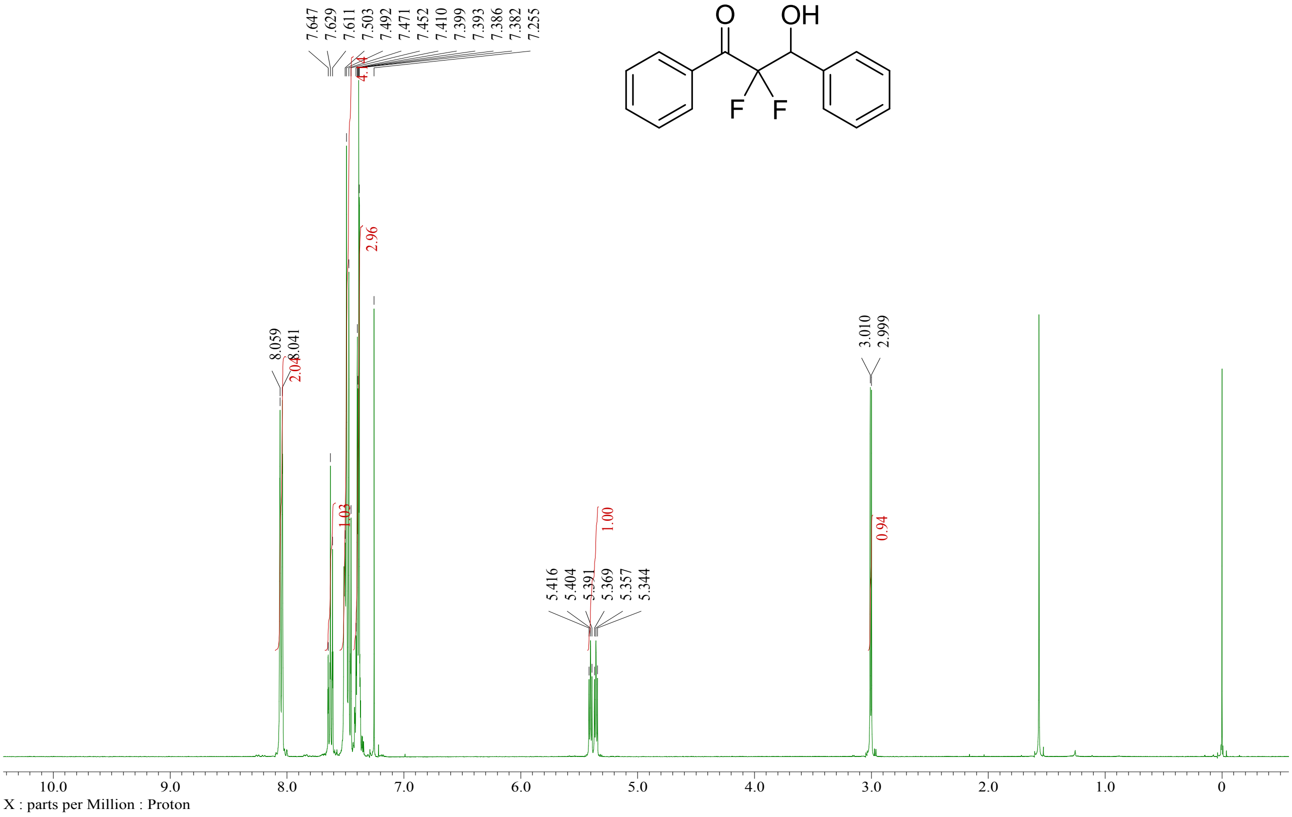

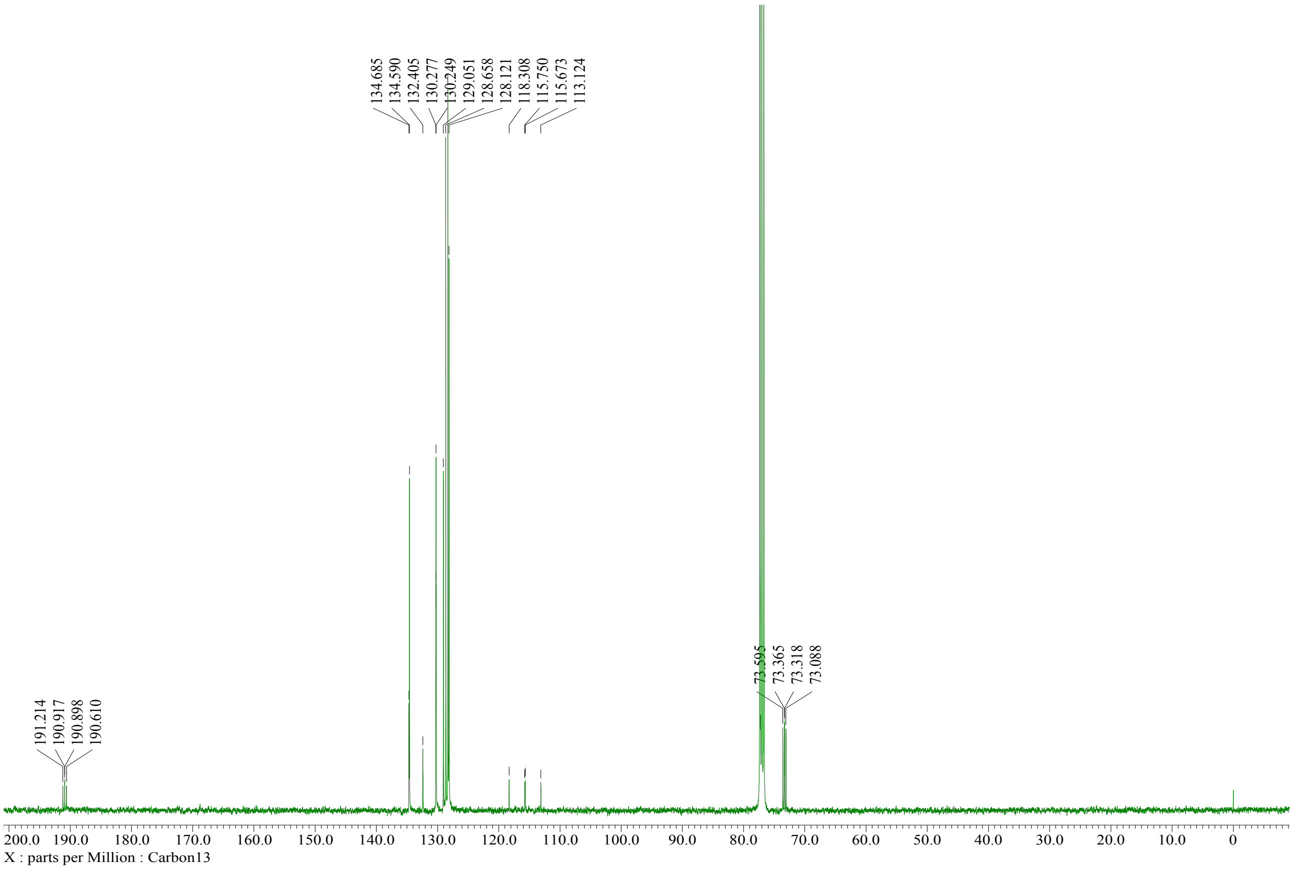

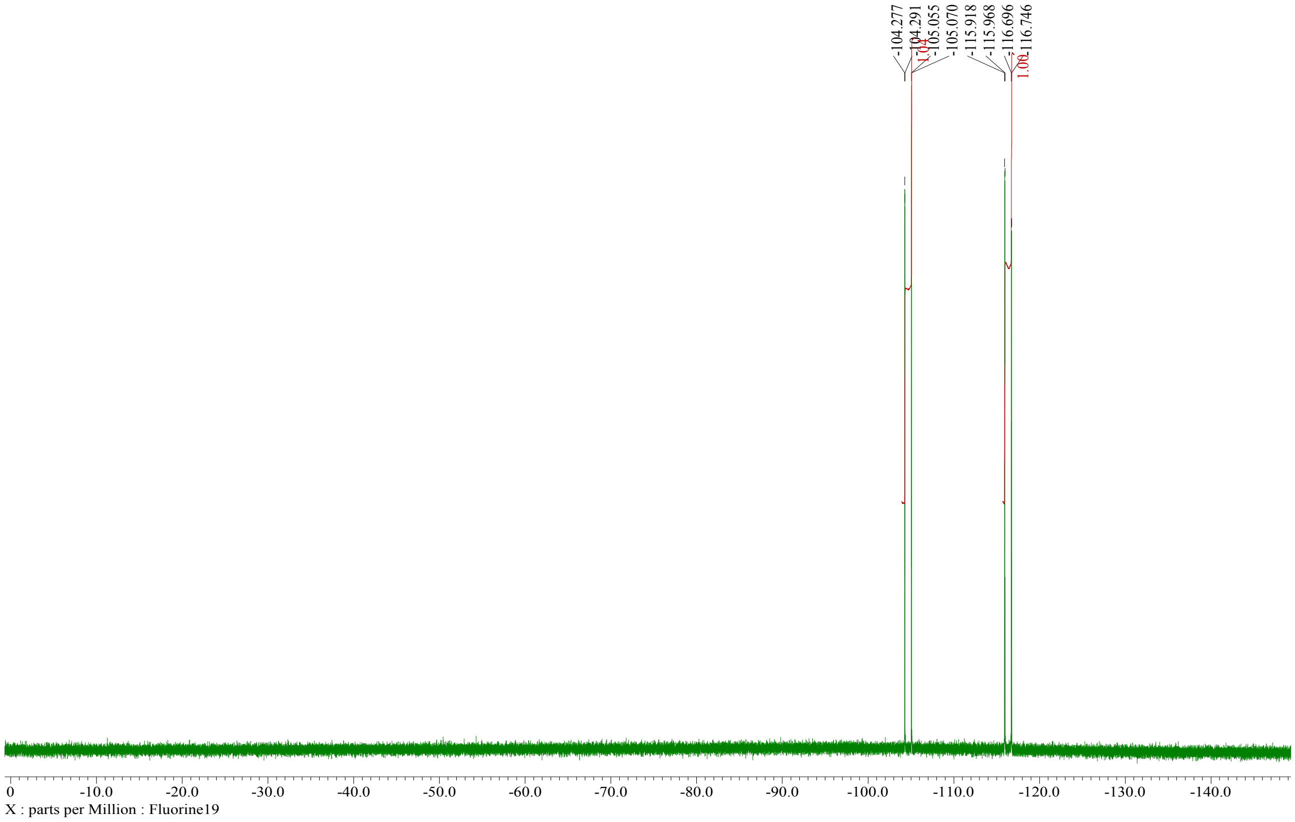

2,2-Difluoro-3-hydroxy-1-phenyl-3-(*p*-tolyl)propan-1-one (6ab)

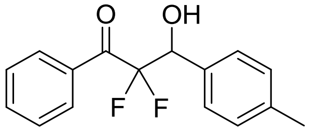

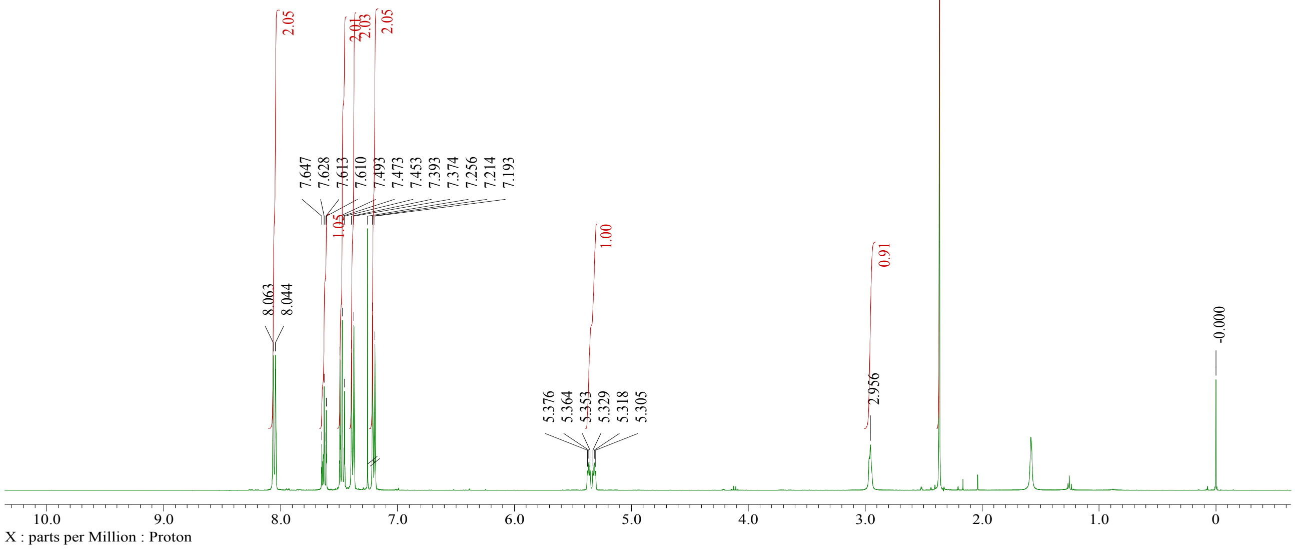

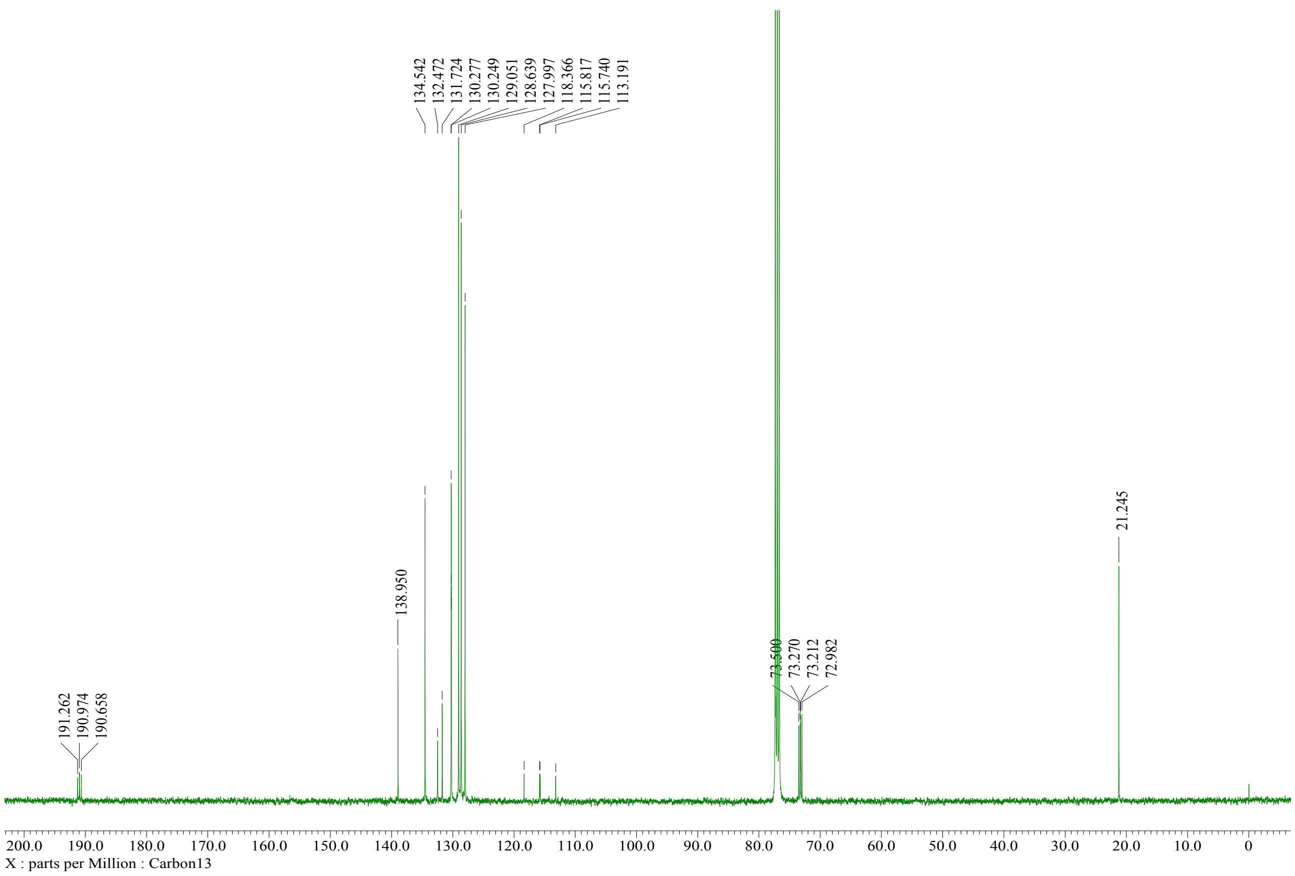
191.262
190.974
190.658
138.950
134.542
132.472
131.724
130.277
130.249
129.051
128.639
127.997
118.366
115.817
115.740
113.191
73.500
73.270
73.212
72.982
21.245
200.0 190.0 180.0 170.0 160.0 150.0 140.0 130.0 120.0 110.0 100.0 90.0 80.0 70.0 60.0 50.0 40.0 30.0 20.0 10.0 0
X : parts per Million : Carbon13

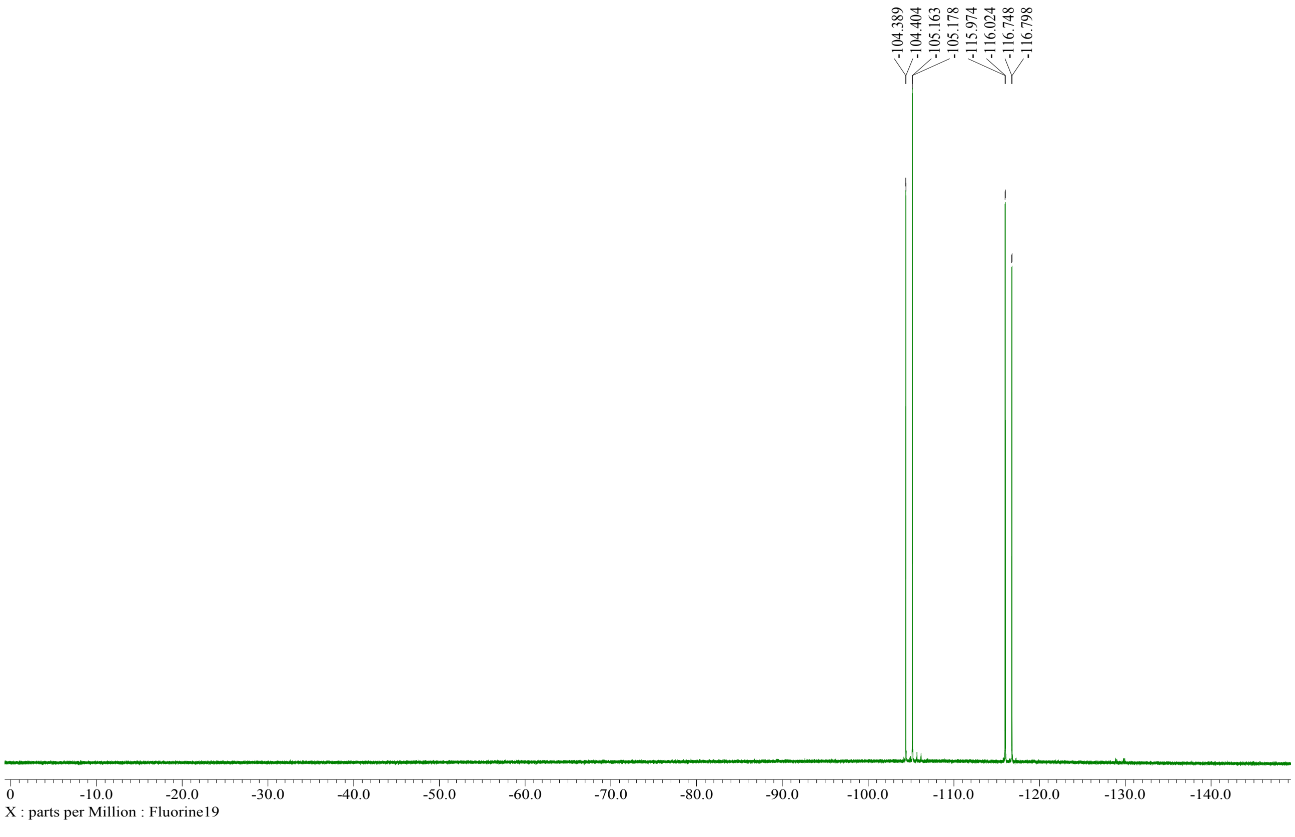
-104.389
-104.404
-105.163
-105.178
-115.974
-116.024
-116.748
-116.798
0 -10.0 -20.0 -30.0 -40.0 -50.0 -60.0 -70.0 -80.0 -90.0 -100.0 -110.0 -120.0 -130.0 -140.0
X : parts per Million : Fluorine19

2,2-Difluoro-3-hydroxy-3-(4-methoxyphenyl)-1-phenylpropan-1-one (6ac)

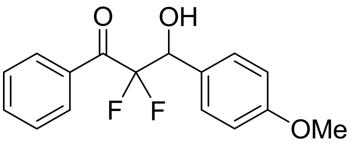

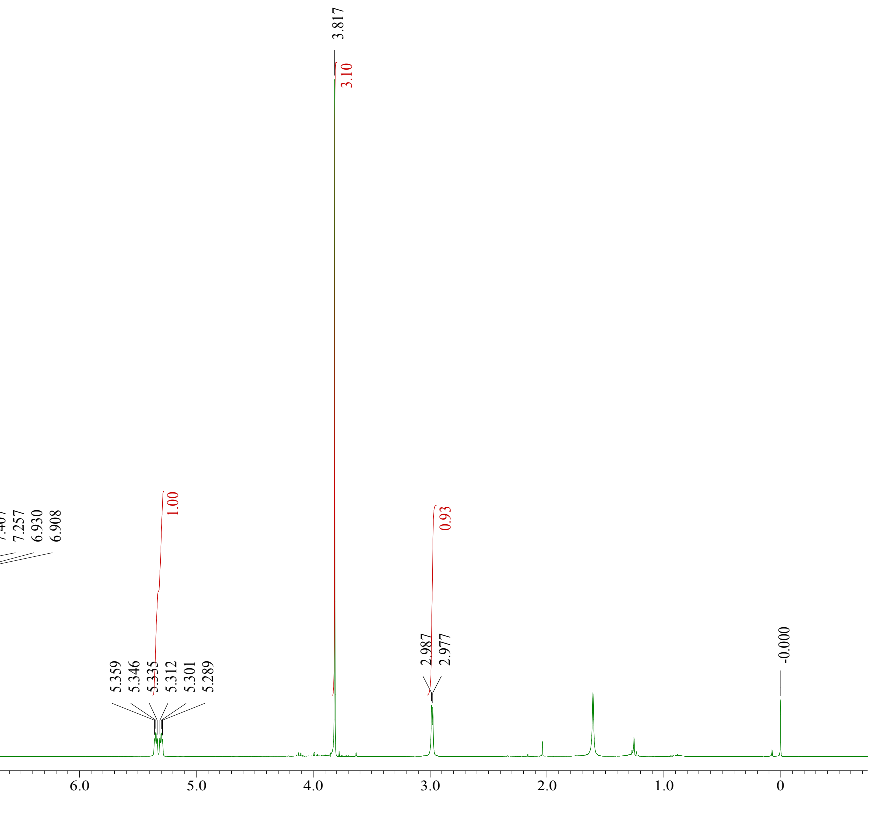

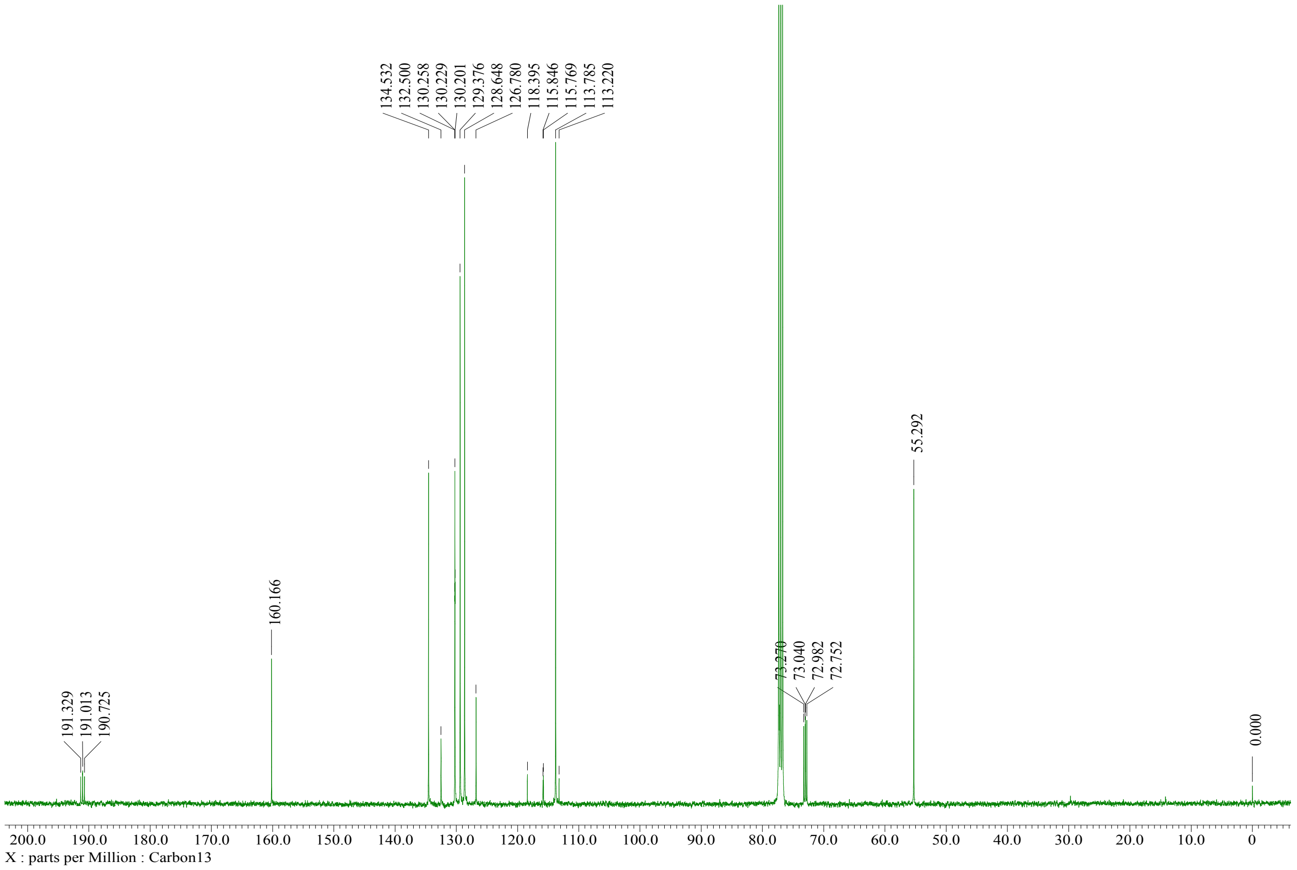

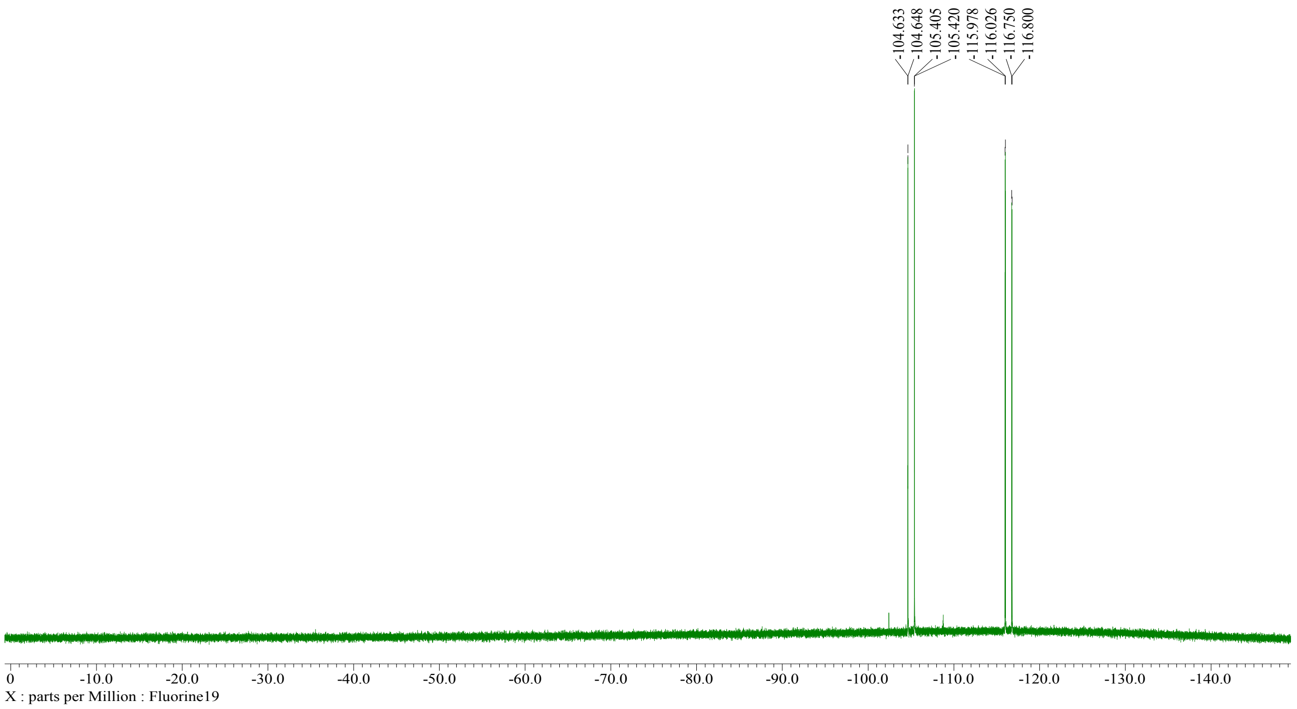

2,2-Difluoro-3-hydroxy-1-phenyl-3-(4-(trifluoromethyl)phenyl)propan-1-one (6ad)

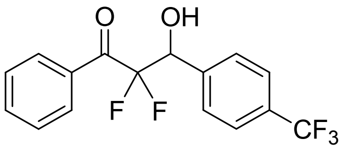

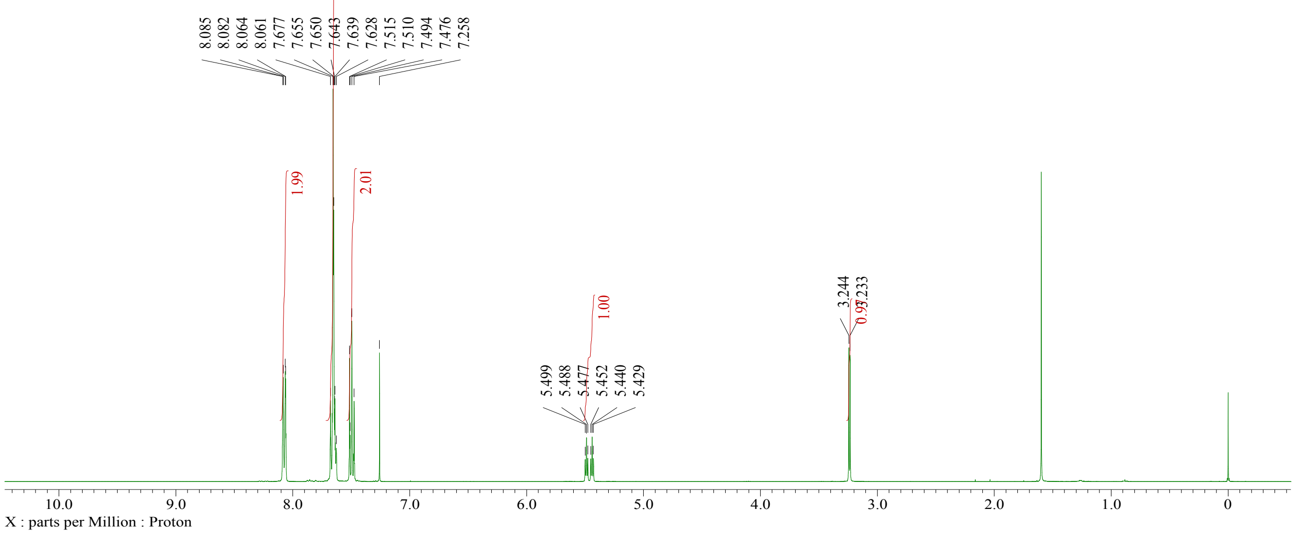

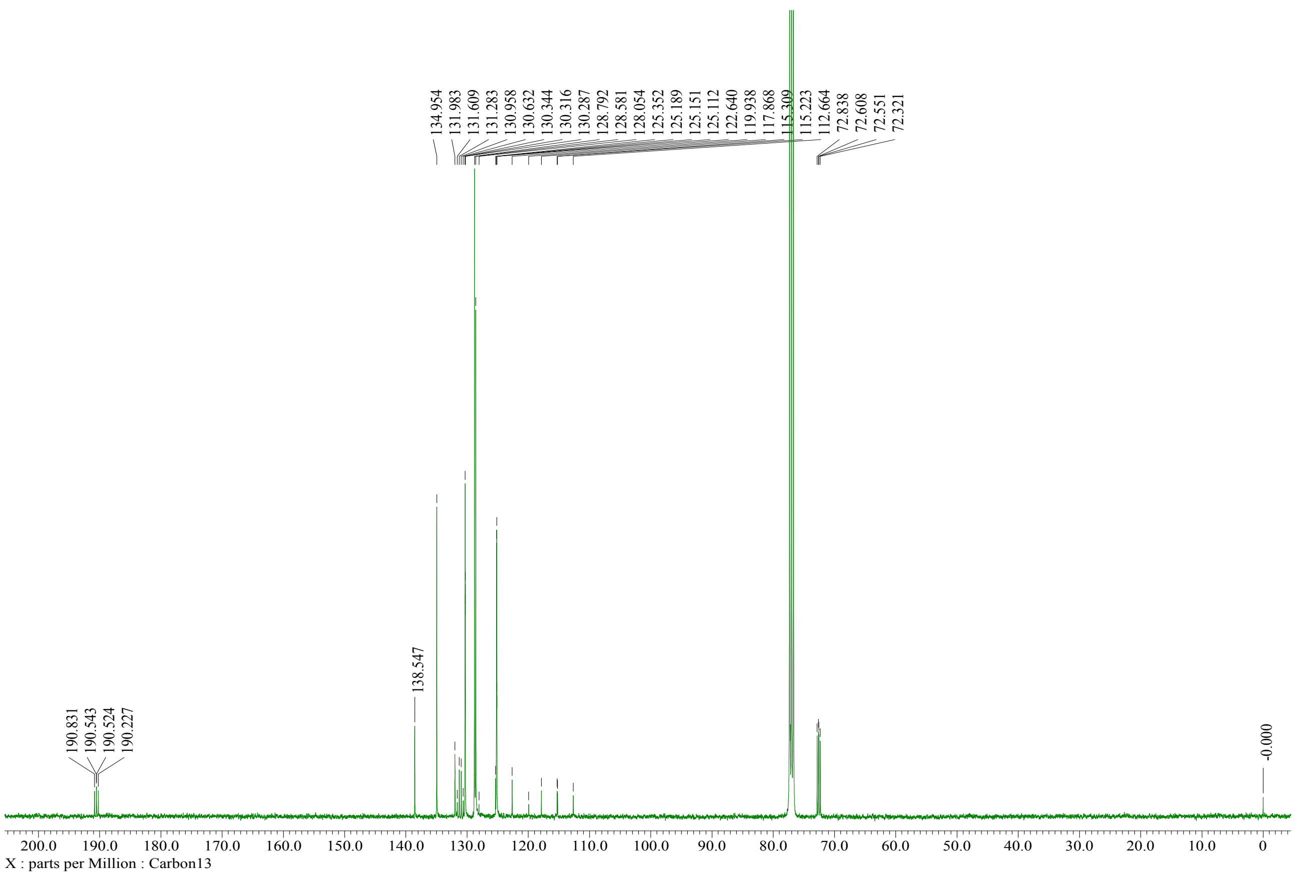

190.831
190.543
190.524
190.227
138.547
134.954
131.983
131.609
131.283
130.958
130.632
130.344
130.316
130.287
128.792
128.581
128.054
125.352
125.189
125.151
125.112
122.640
119.938
117.868
115.309
115.223
112.664
72.838
72.608
72.551
72.321
-0.000
200.0 190.0 180.0 170.0 160.0 150.0 140.0 130.0 120.0 110.0 100.0 90.0 80.0 70.0 60.0 50.0 40.0 30.0 20.0 10.0 0
X : parts per Million : Carbon13

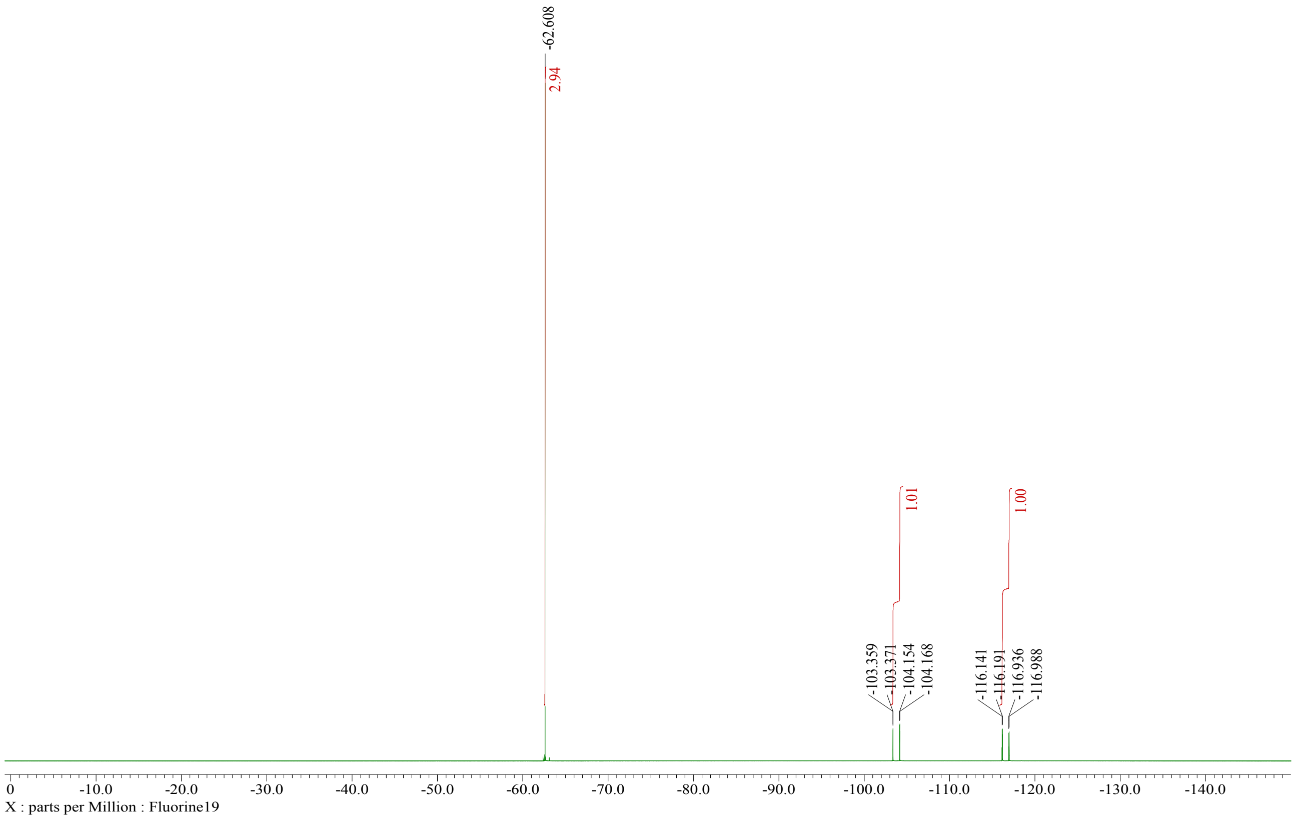

-62.608
2.94
1.01
1.00
-103.359
-103.371
-104.154
-104.168
-116.141
-116.191
-116.936
-116.988
0 -10.0 -20.0 -30.0 -40.0 -50.0 -60.0 -70.0 -80.0 -90.0 -100.0 -110.0 -120.0 -130.0 -140.0
X : parts per Million : Fluorine19

3-(4-Bromophenyl)-2,2-difluoro-3-hydroxy-1-phenylpropan-1-one (6ae)

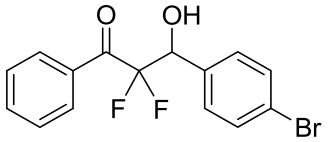

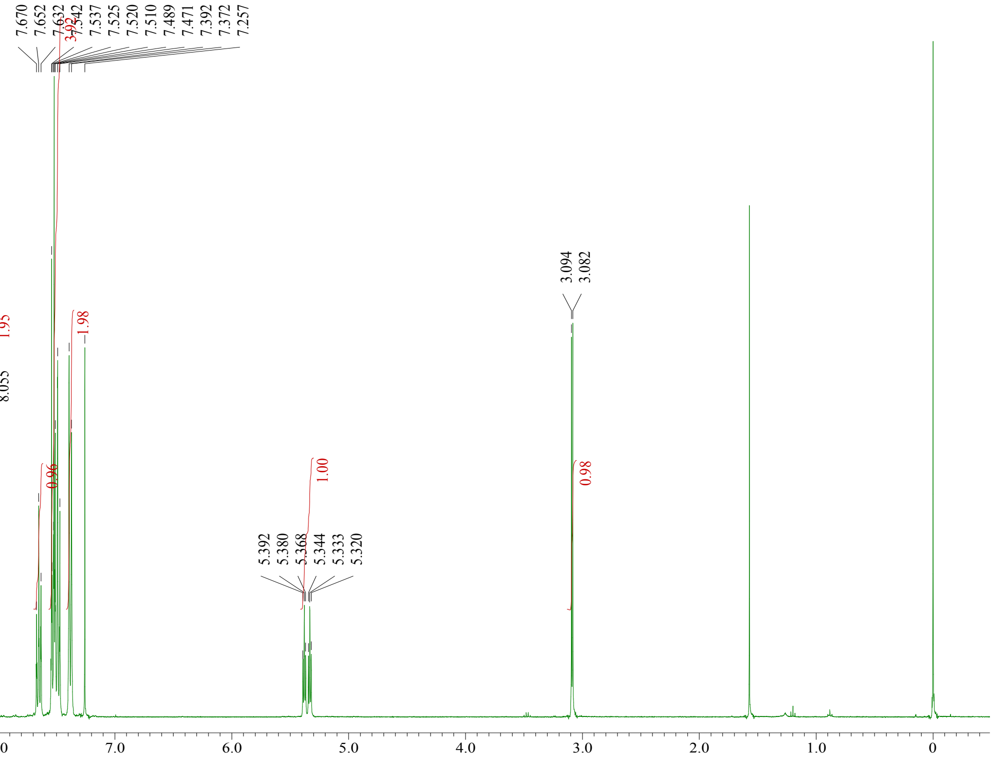

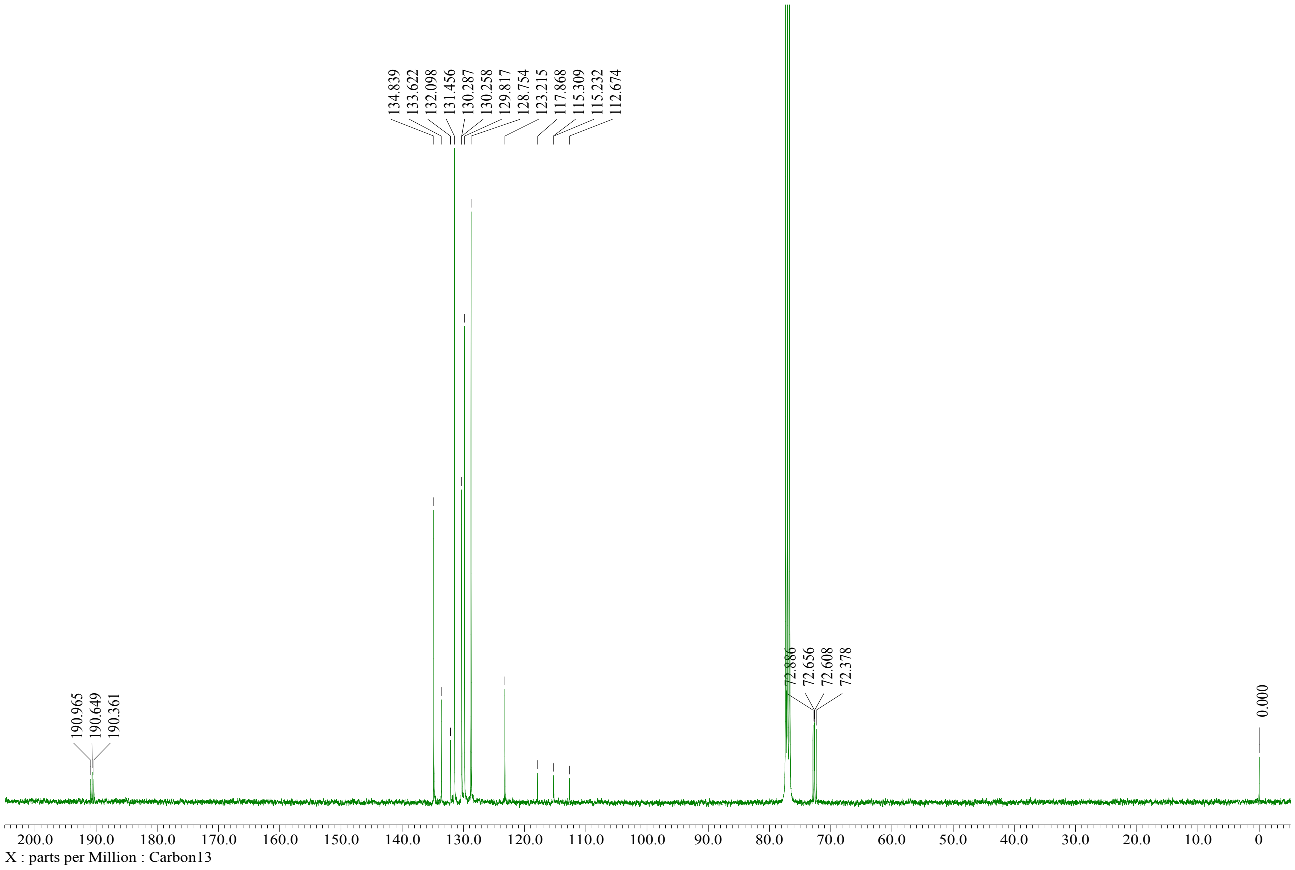

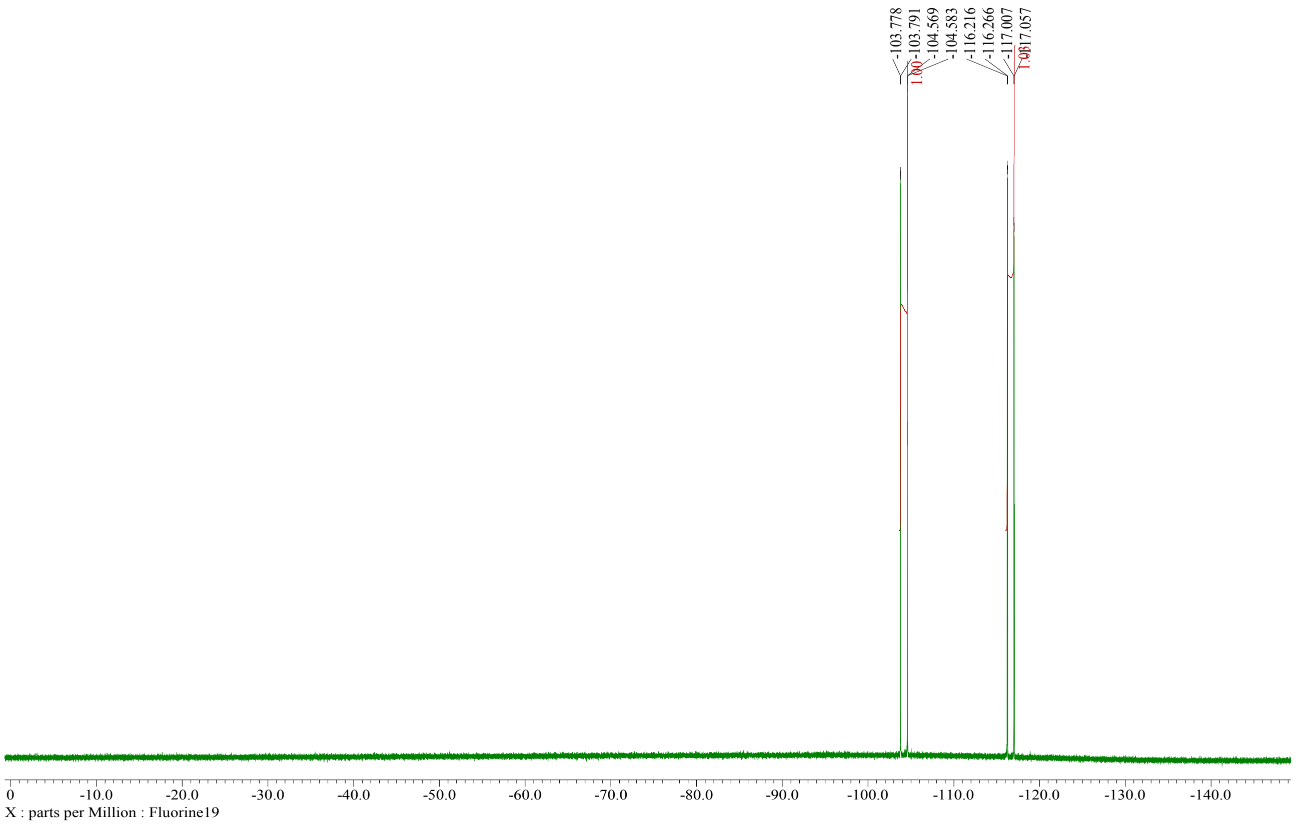

2,2-Difluoro-3-hydroxy-3-(4-nitrophenyl)-1-phenylpropan-1-one (6af)

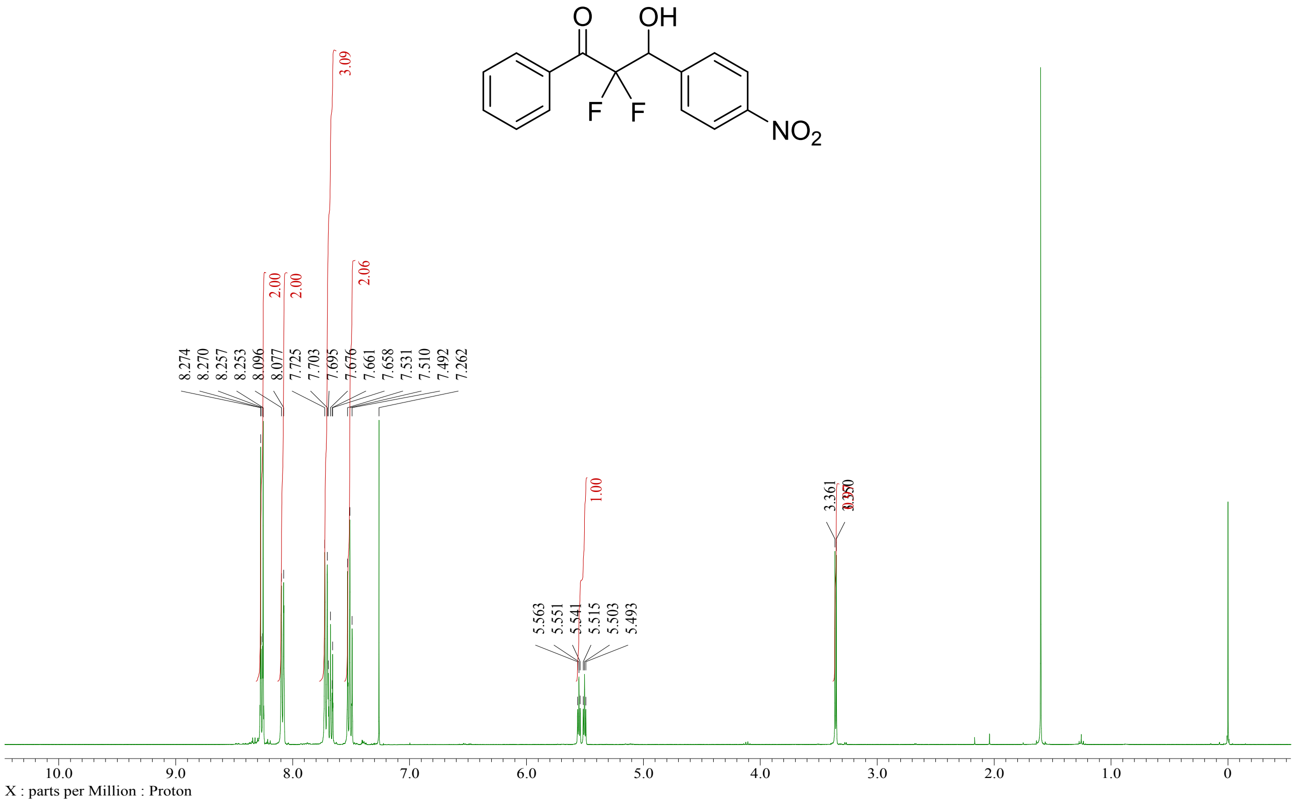

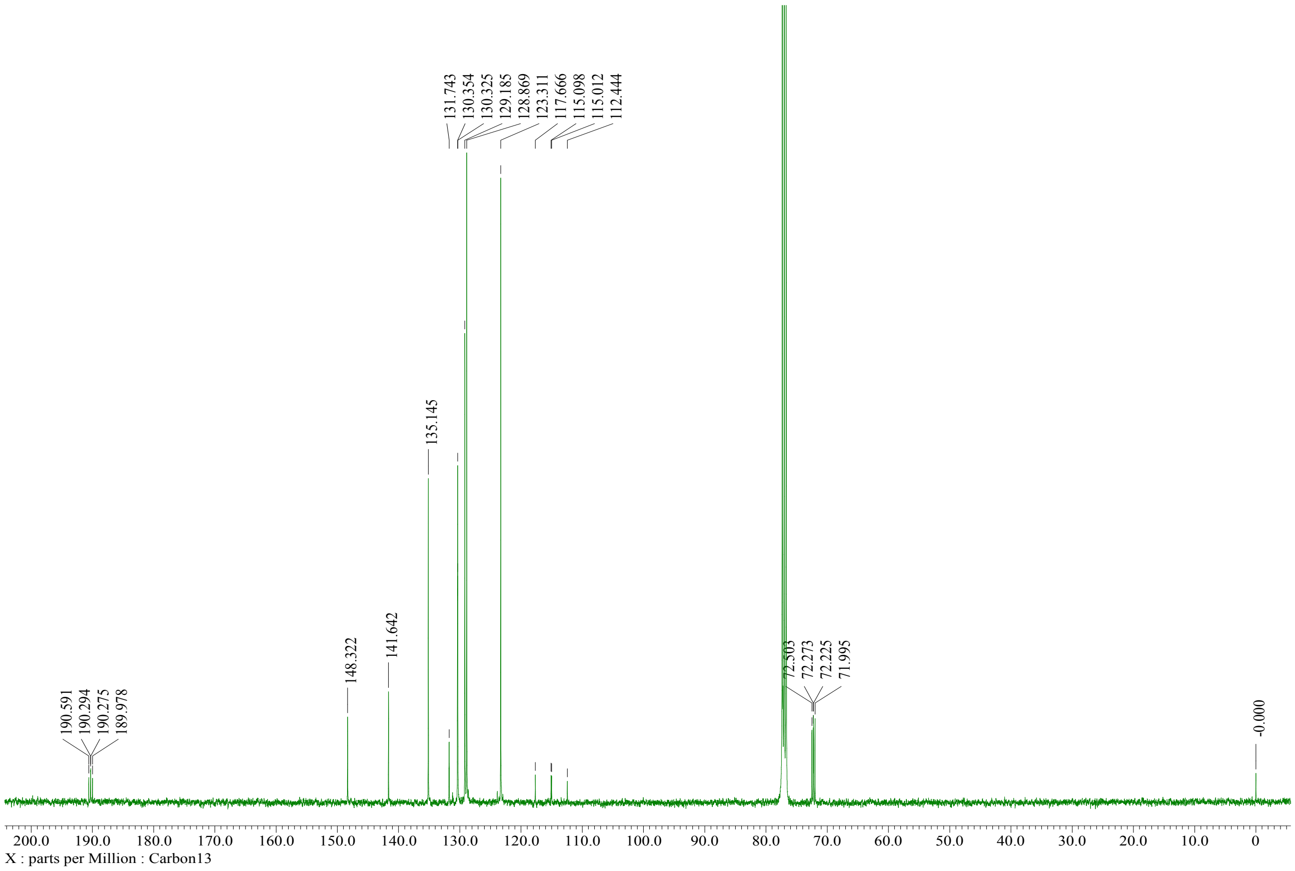
190.591
190.294
190.275
189.978
148.322
141.642
135.145
131.743
130.354
130.325
129.185
128.869
123.311
117.666
115.098
115.012
112.444
72.503
72.273
72.225
71.995
-0.000
200.0 190.0 180.0 170.0 160.0 150.0 140.0 130.0 120.0 110.0 100.0 90.0 80.0 70.0 60.0 50.0 40.0 30.0 20.0 10.0 0
X : parts per Million : Carbon13

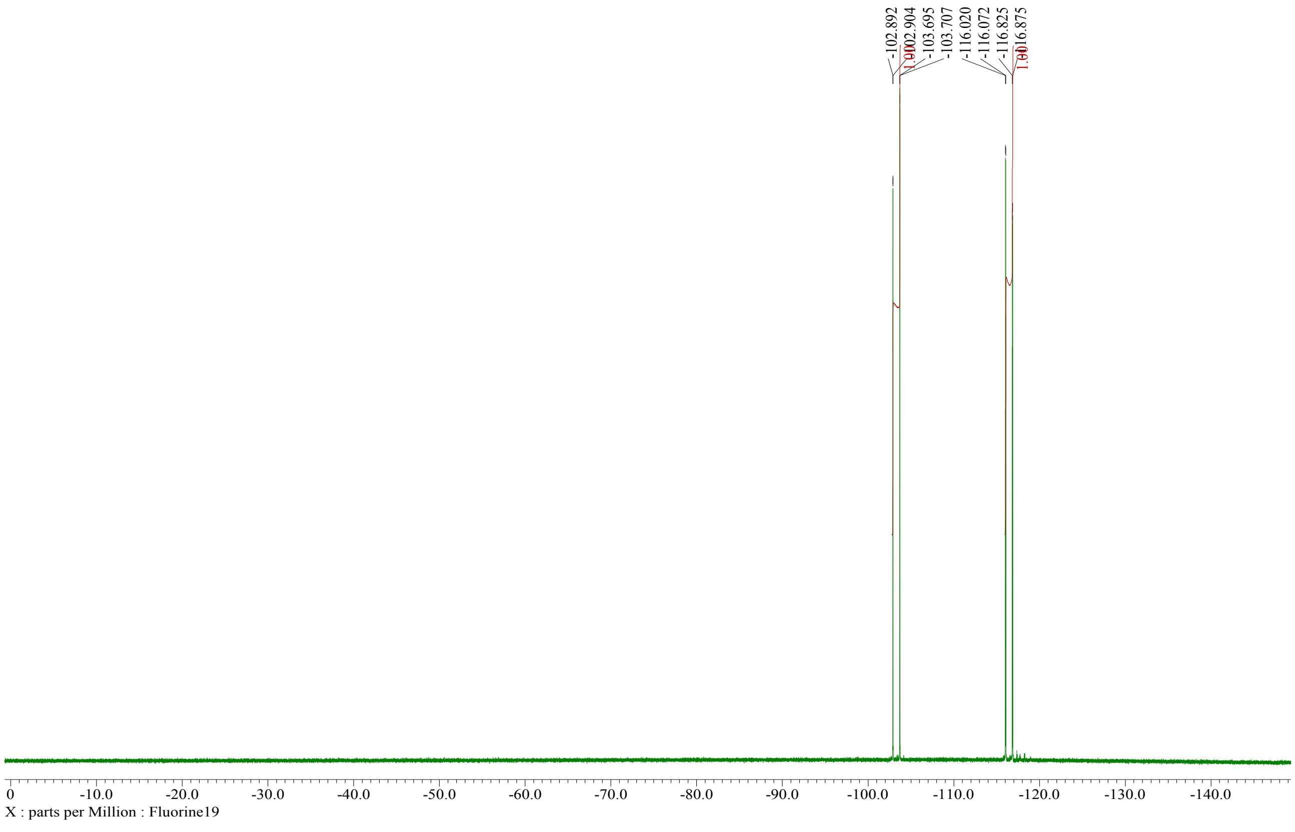
-102.892
-102.904
-103.695
-103.707
-116.020
-116.072
-116.825
-116.875
0 -10.0 -20.0 -30.0 -40.0 -50.0 -60.0 -70.0 -80.0 -90.0 -100.0 -110.0 -120.0 -130.0 -140.0
X : parts per Million : Fluorine19

Methyl 4-(2,2-difluoro-1-hydroxy-3-oxo-3-phenylpropyl)benzoate (6ag)

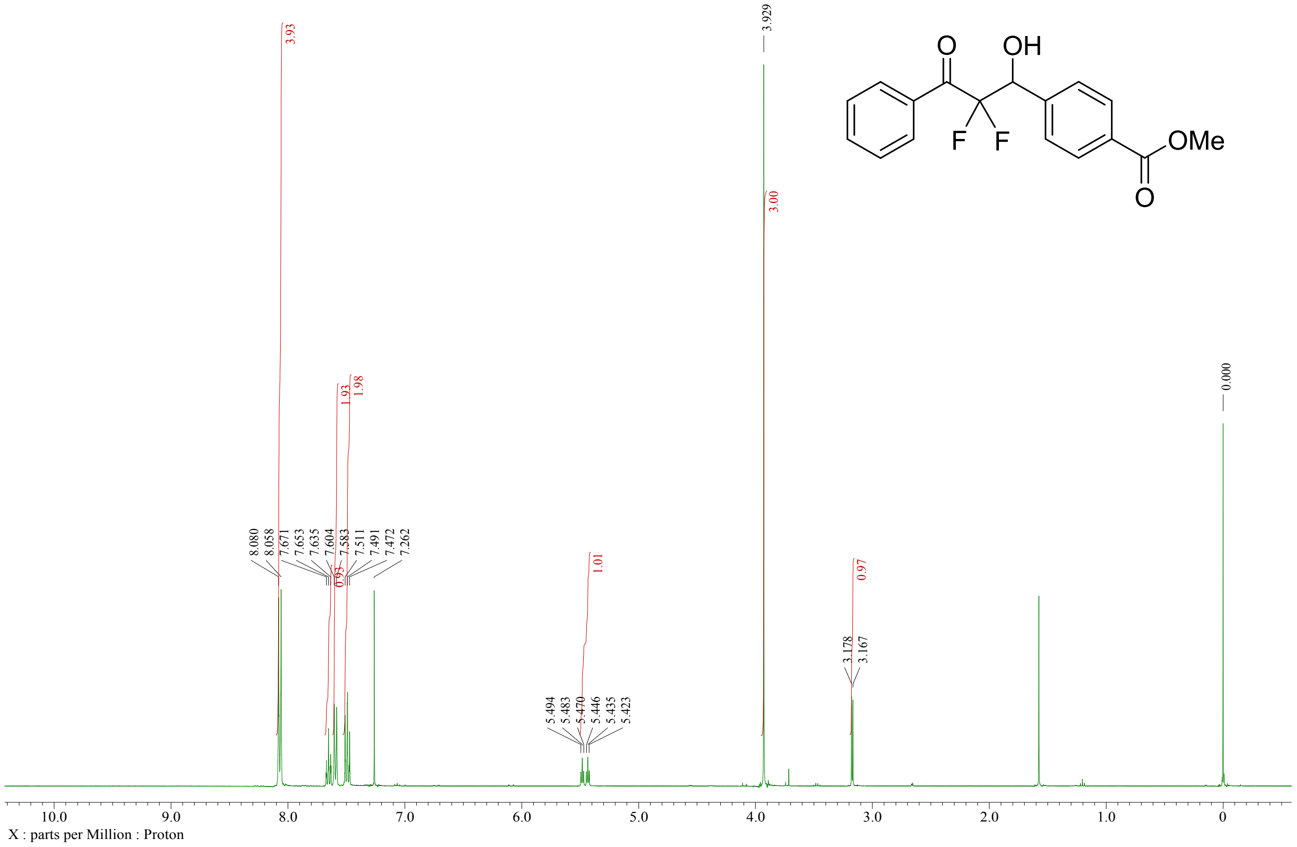

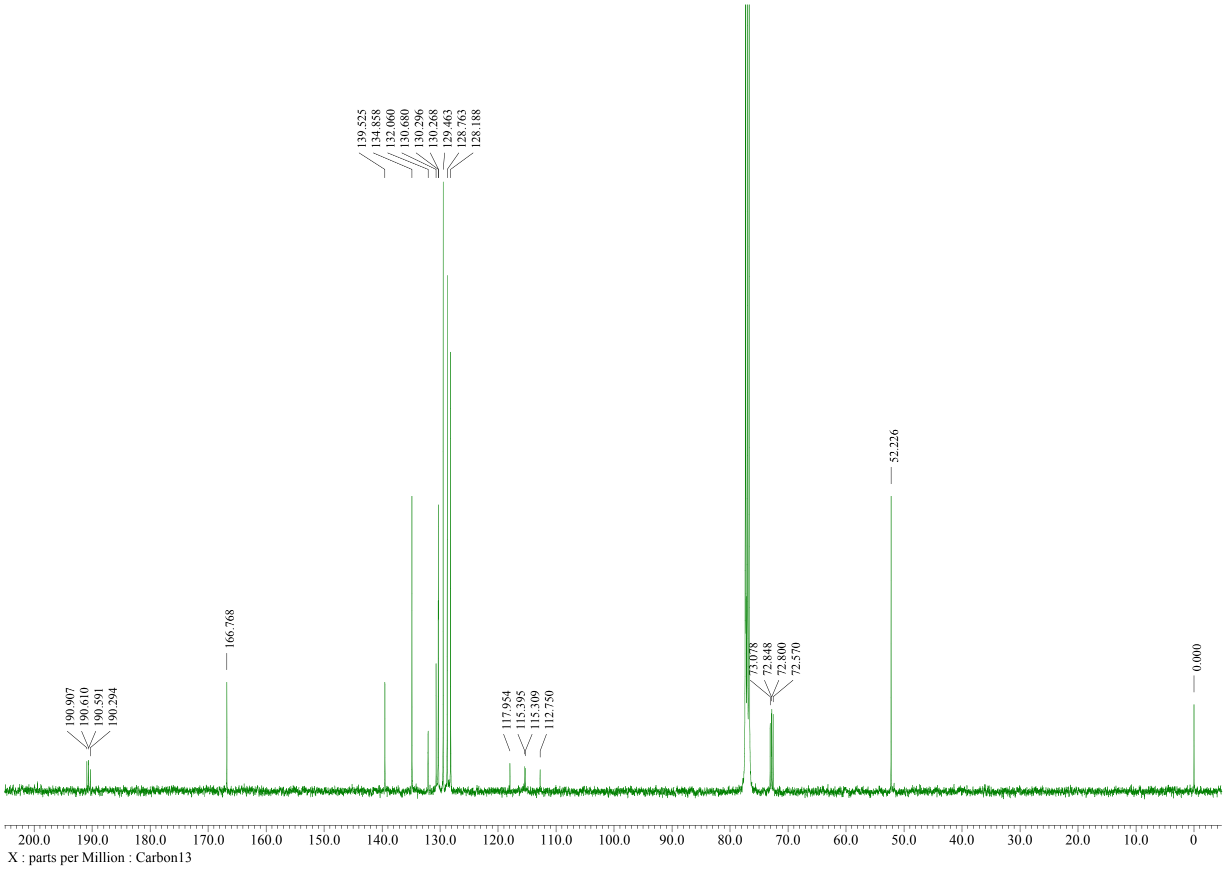

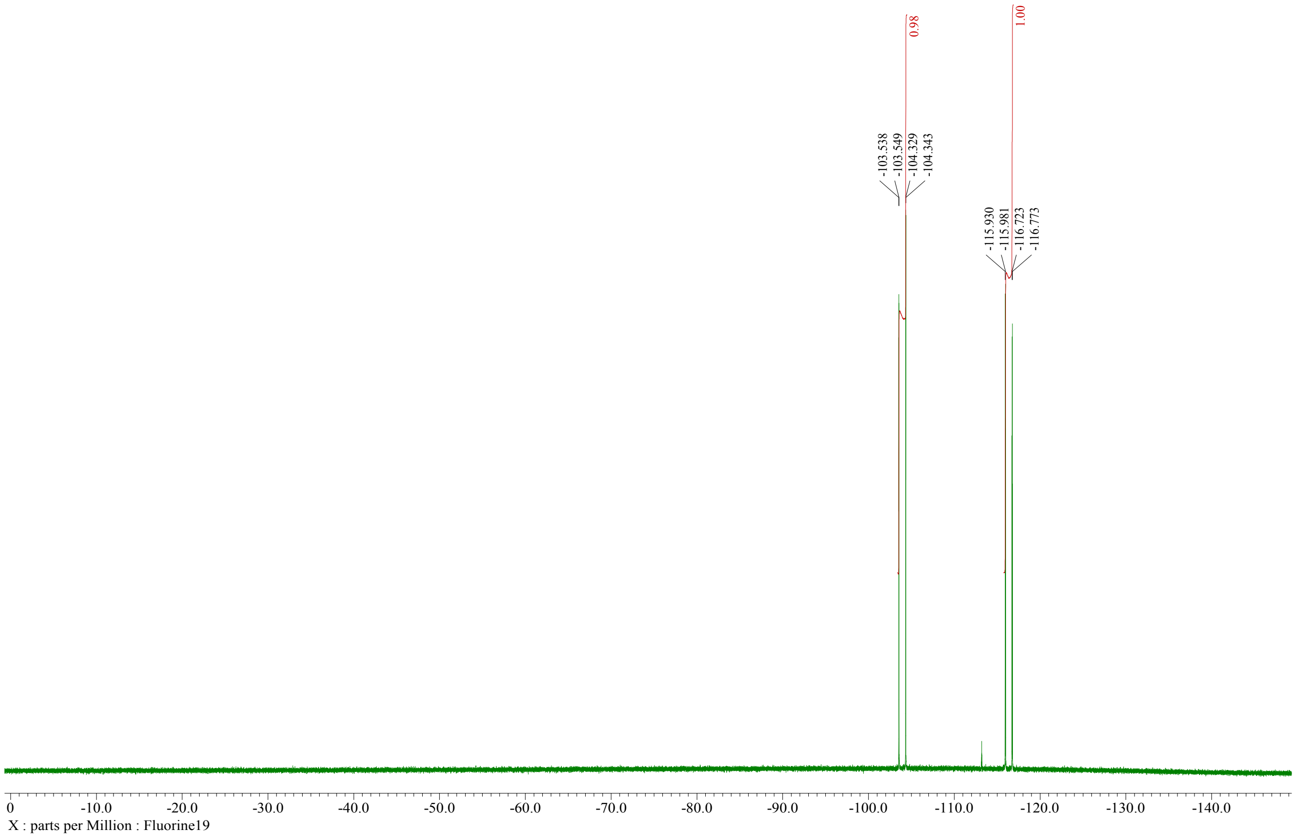

4-(2,2-Difluoro-1-hydroxy-3-oxo-3-phenylpropyl)benzonitrile (6ah)

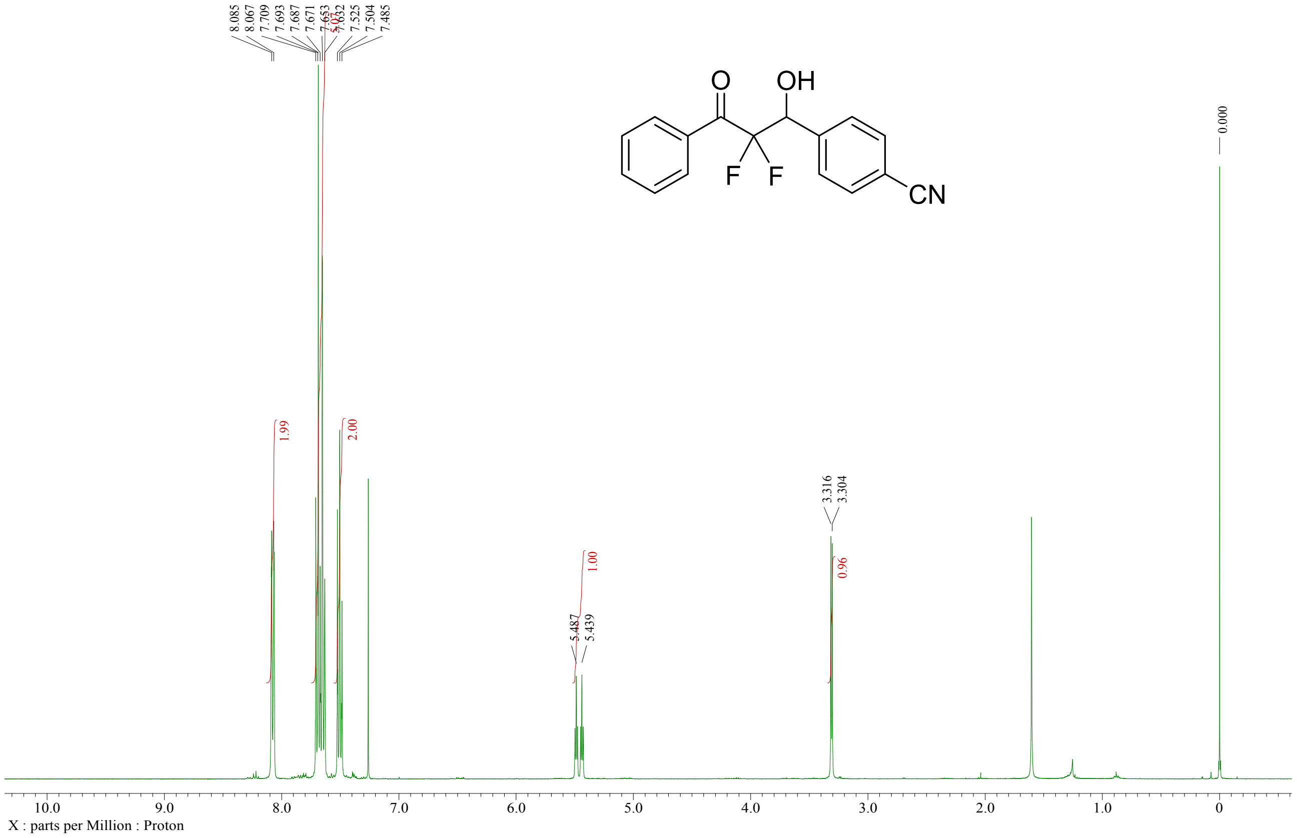

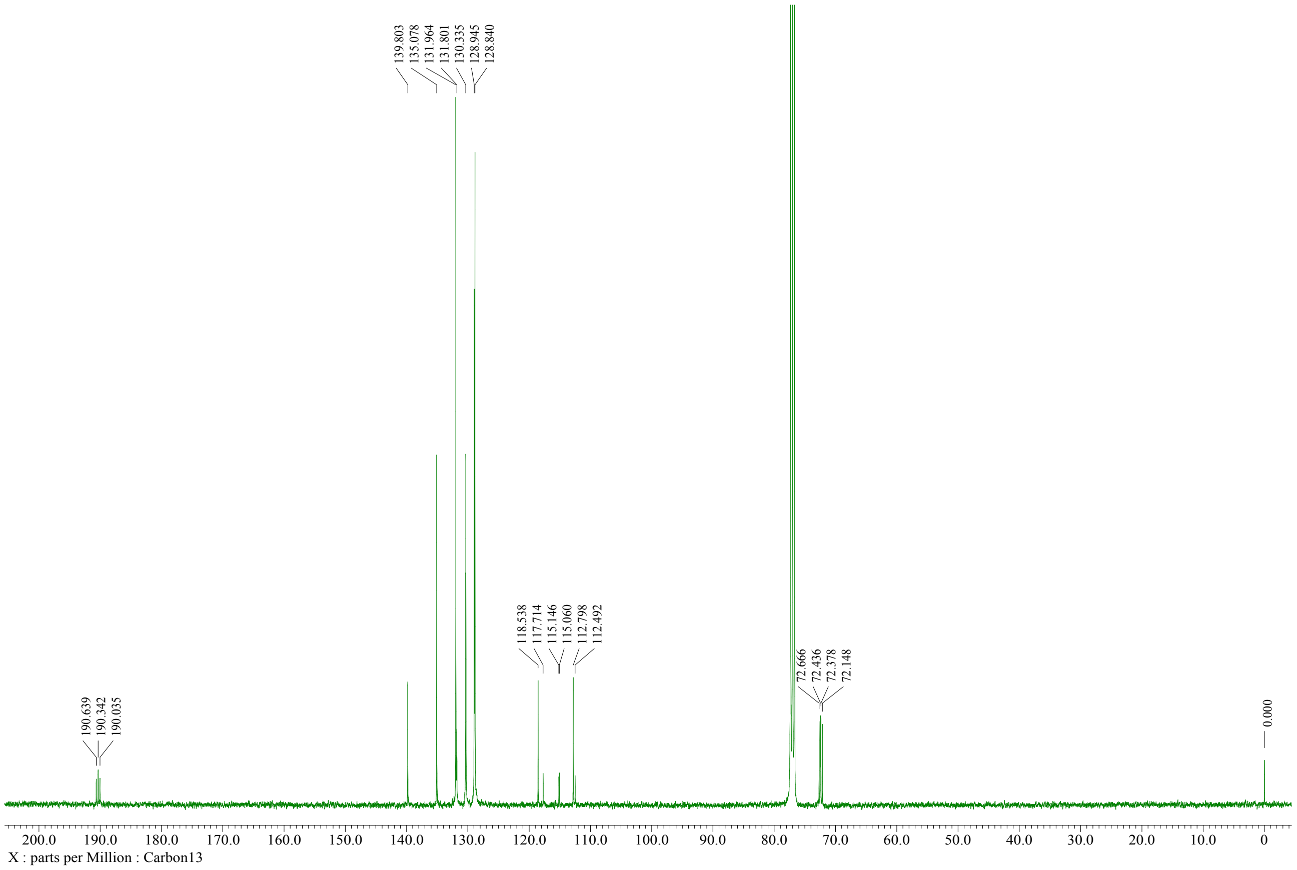

139.803
135.078
131.964
131.801
130.335
128.945
128.840
118.538
117.714
115.146
115.060
112.798
112.492
72.666
72.436
72.378
72.148
190.639
190.342
190.035
0.000
200.0 190.0 180.0 170.0 160.0 150.0 140.0 130.0 120.0 110.0 100.0 90.0 80.0 70.0 60.0 50.0 40.0 30.0 20.0 10.0 0
X : parts per Million : Carbon13

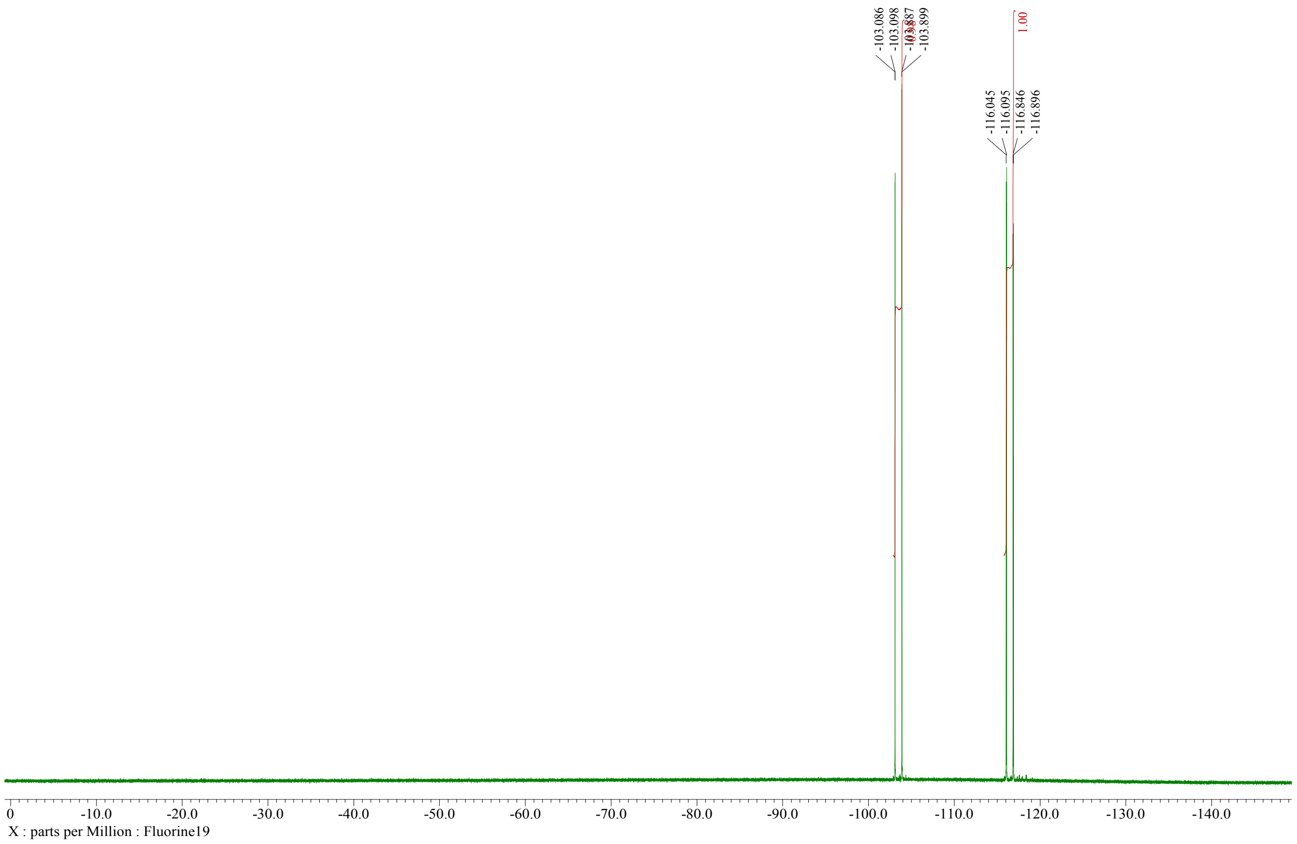

-103.086
-103.098
-103.887
-103.899
1.00
-116.045
-116.095
-116.846
-116.896
0 -10.0 -20.0 -30.0 -40.0 -50.0 -60.0 -70.0 -80.0 -90.0 -100.0 -110.0 -120.0 -130.0 -140.0
X : parts per Million : Fluorine19

2,2-Difluoro-3-hydroxy-1-phenyl-3-(*o*-tolyl)propan-1-one (6ai)

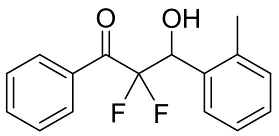

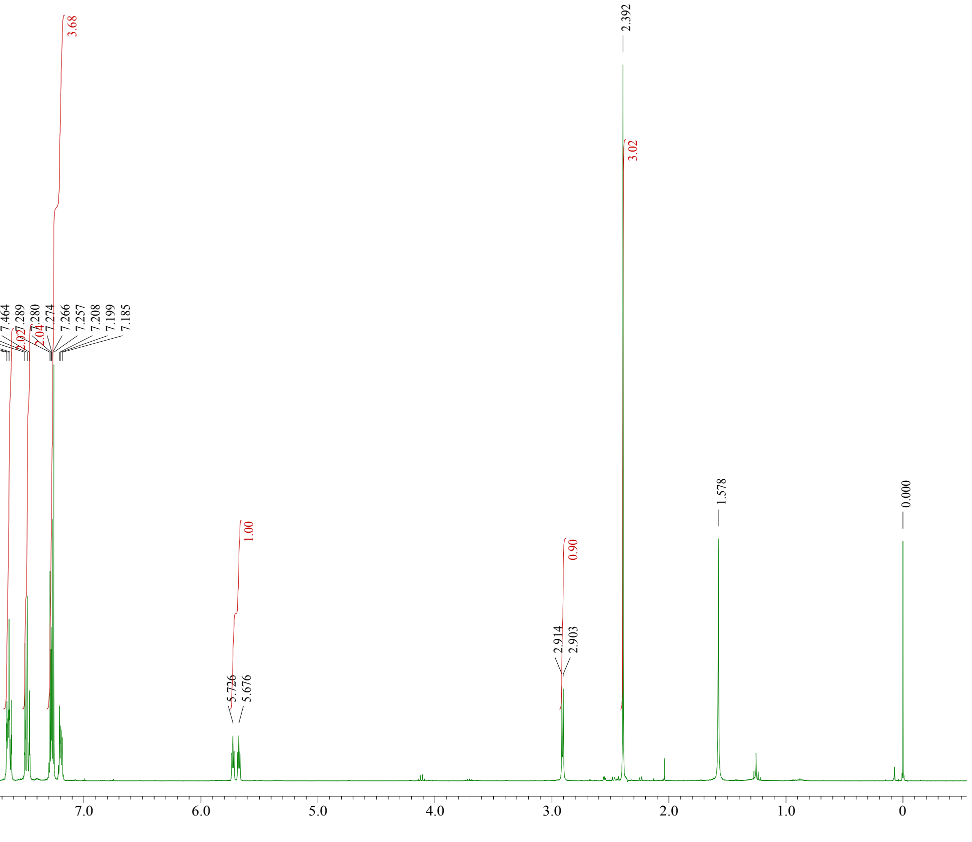

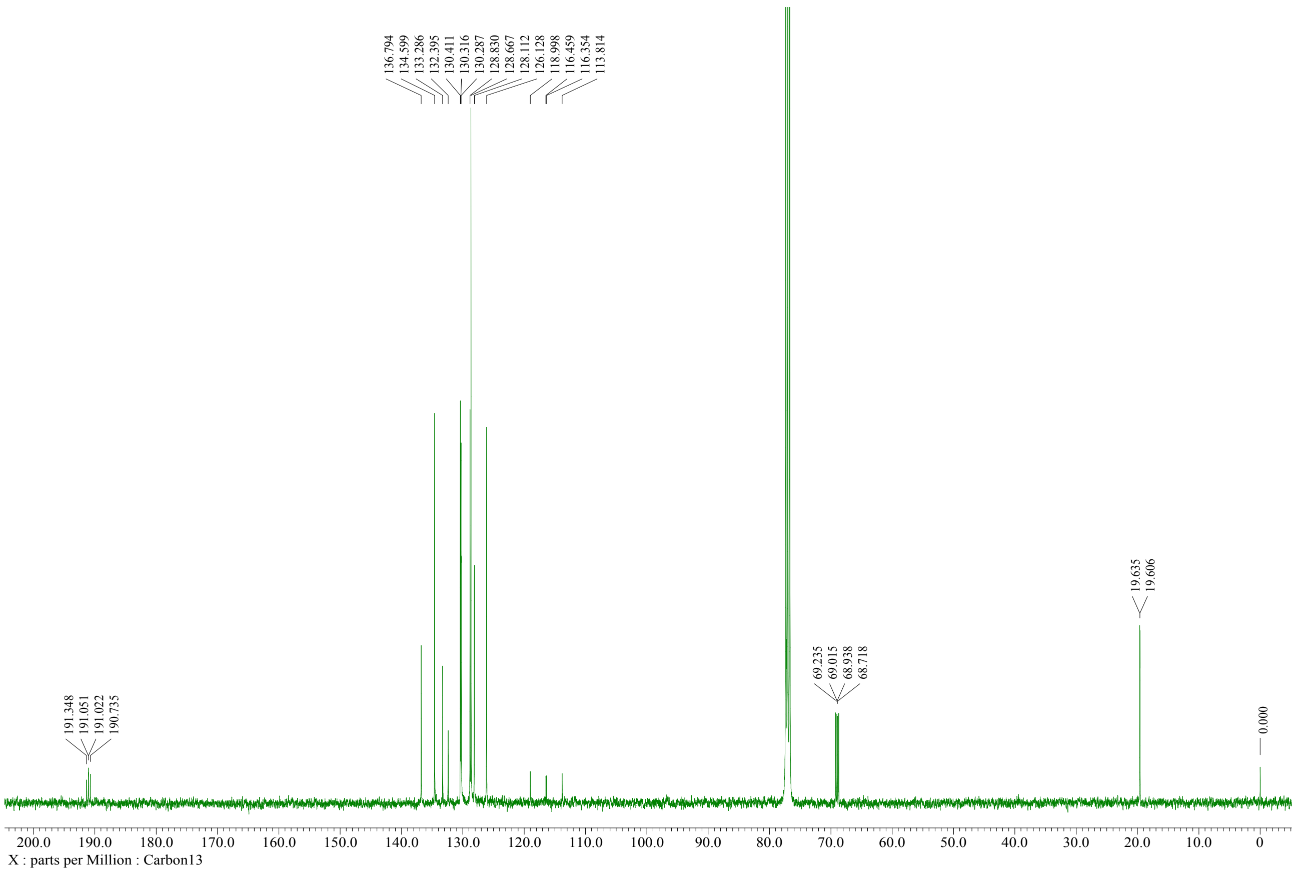

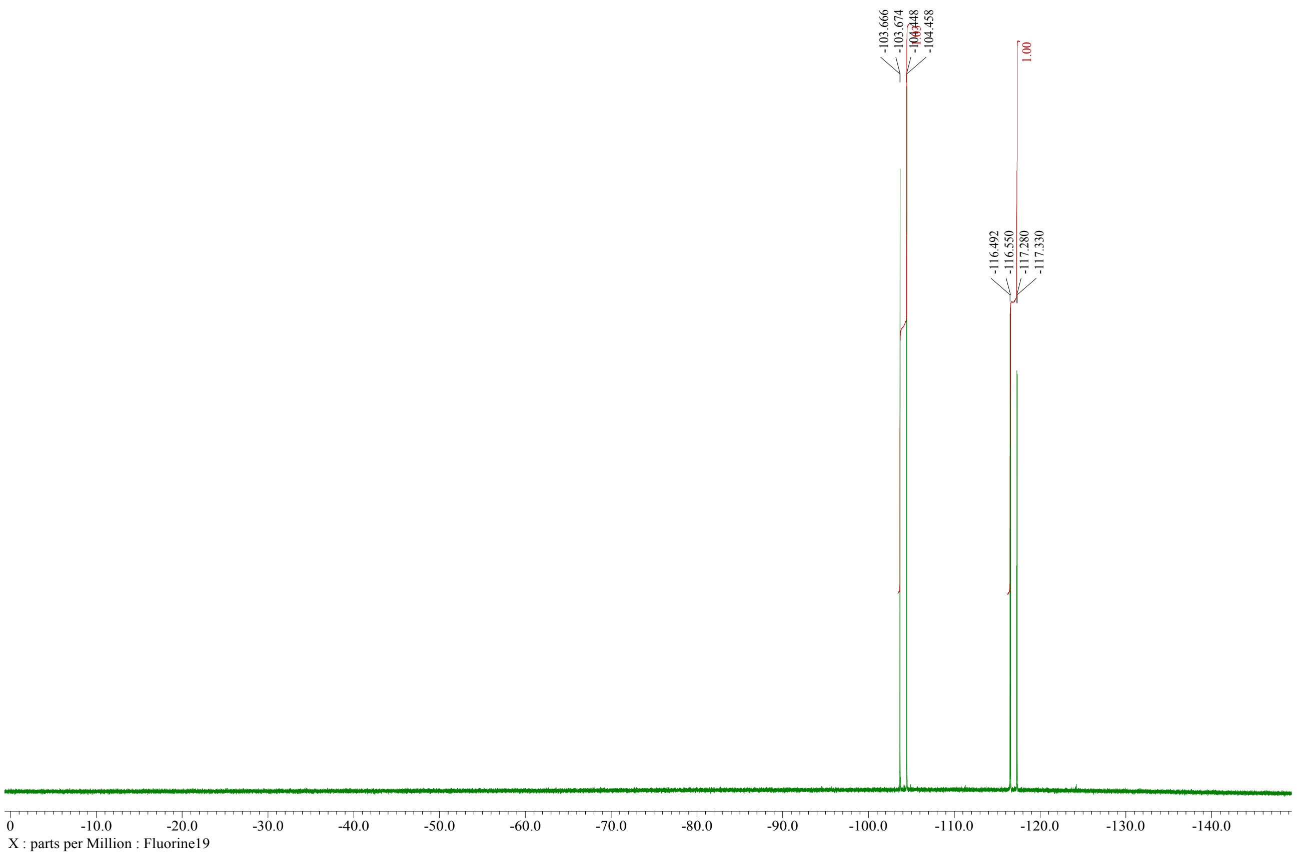

2,2-Difluoro-3-hydroxy-3-(2-methoxyphenyl)-1-phenylpropan-1-one (6aj)

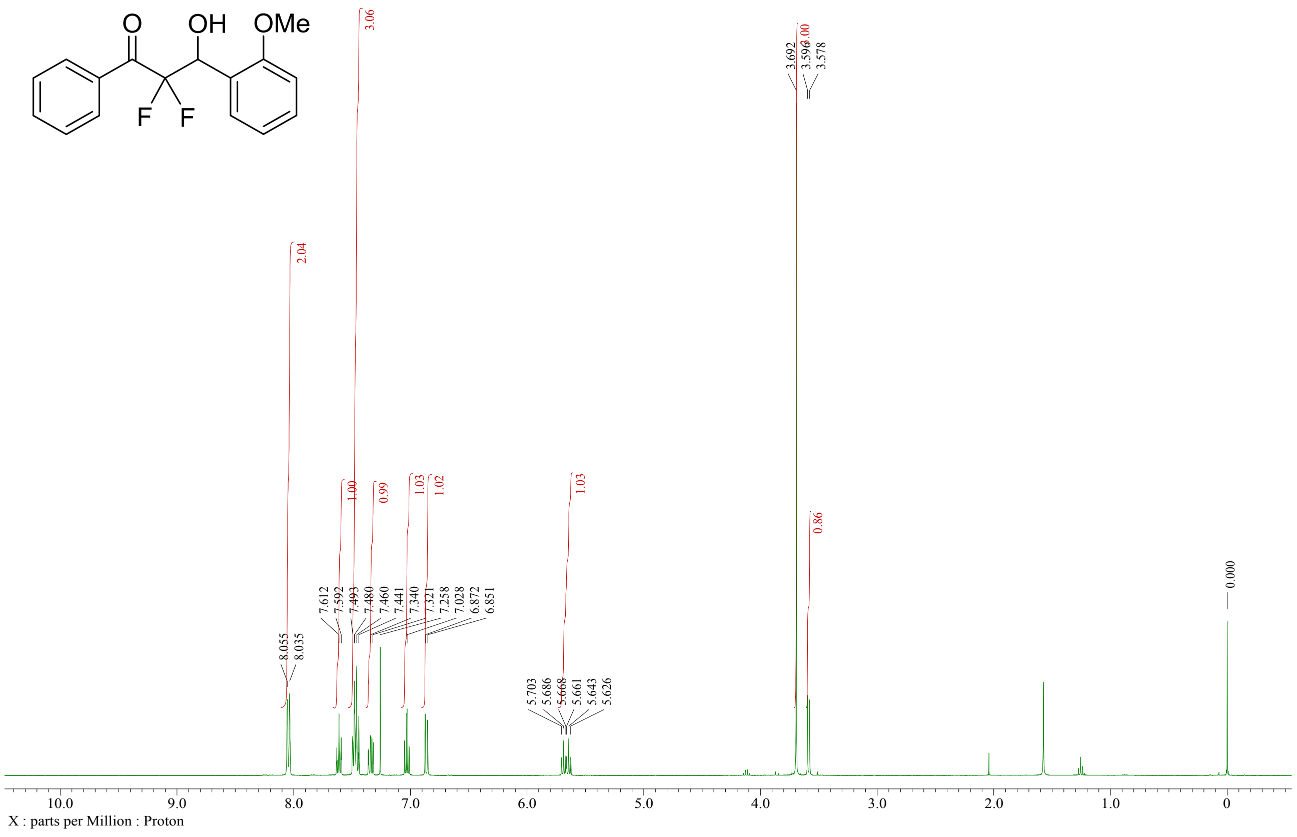

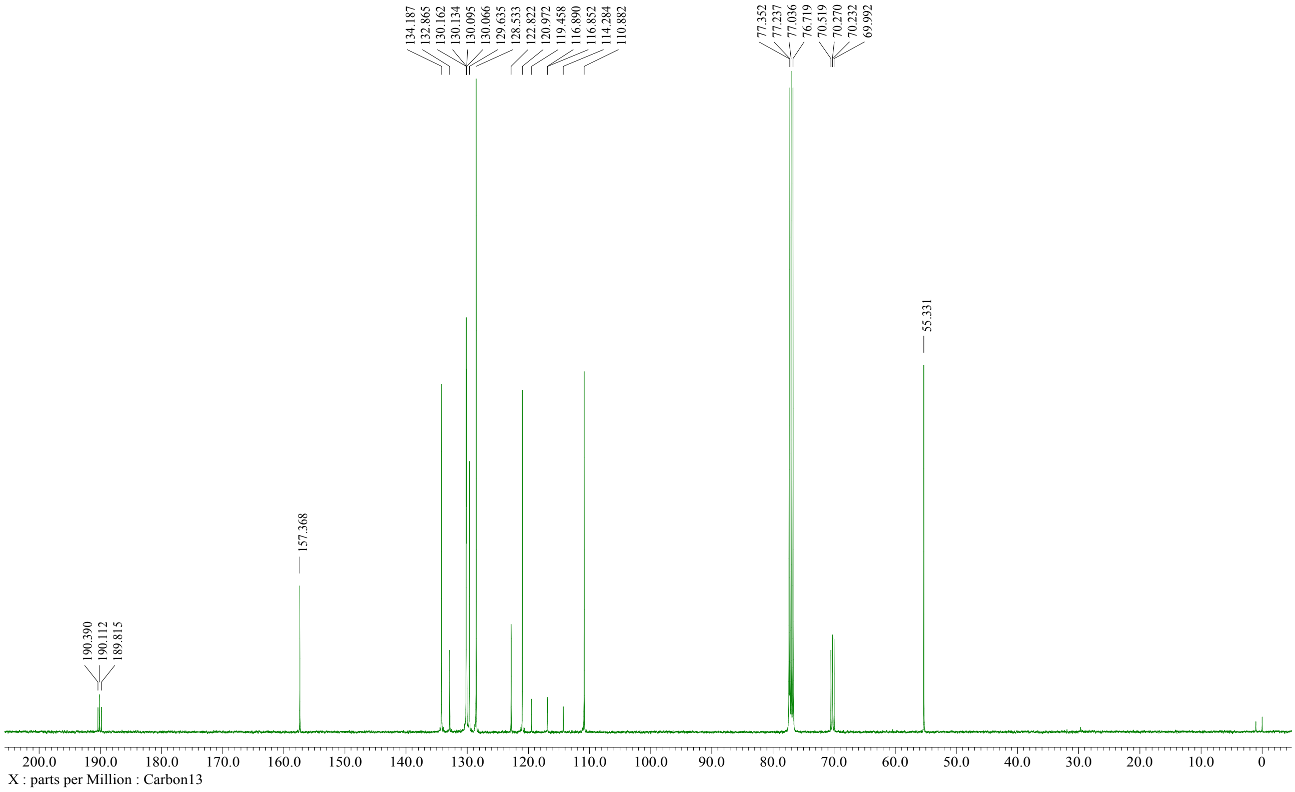
134.187
132.865
130.162
130.134
130.095
130.066
129.635
128.533
122.822
120.972
119.458
116.890
116.852
114.284
110.882
77.352
77.237
77.036
76.719
70.519
70.270
70.232
69.992
55.331
157.368
190.390
190.112
189.815
200.0 190.0 180.0 170.0 160.0 150.0 140.0 130.0 120.0 110.0 100.0 90.0 80.0 70.0 60.0 50.0 40.0 30.0 20.0 10.0 0
X : parts per Million : Carbon13

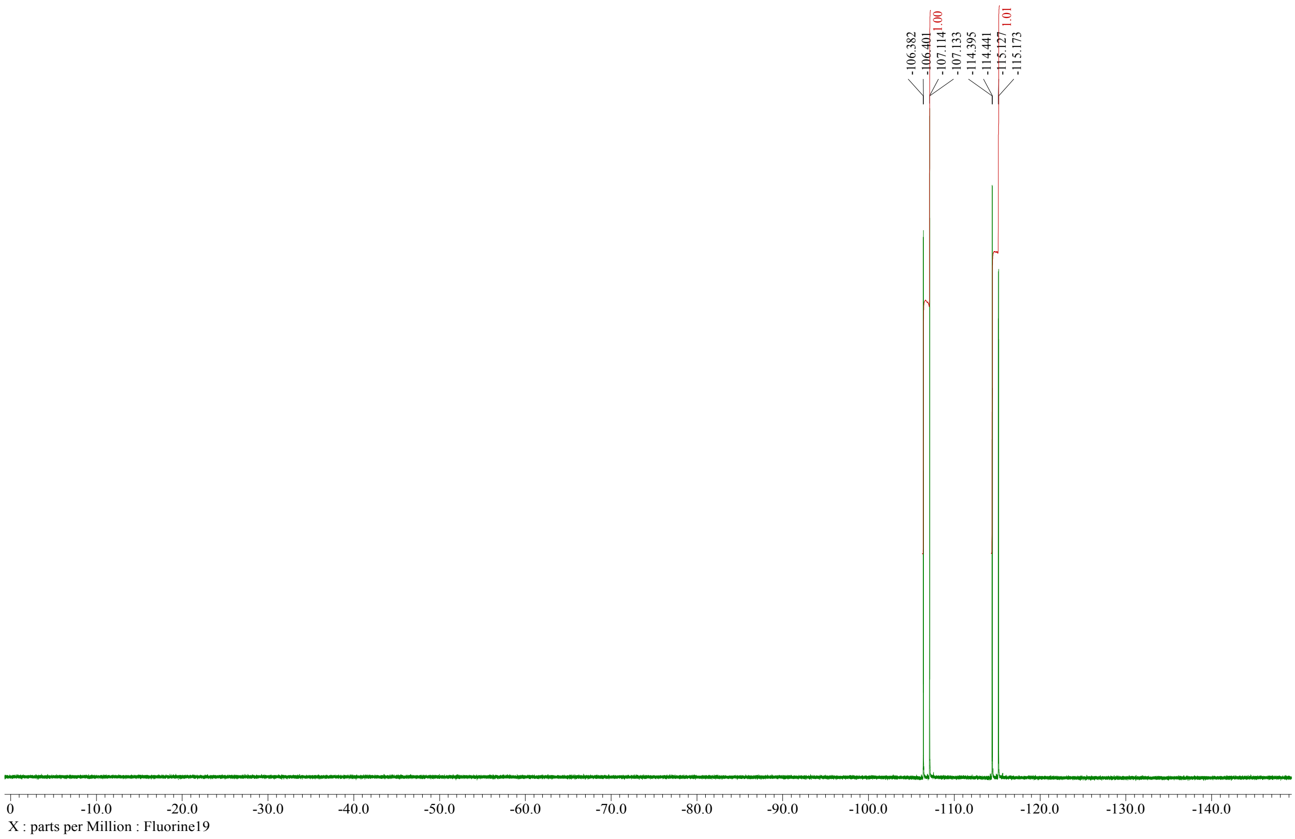
-106.382
-106.401
-107.114
-107.133
-114.395
-114.441
-115.127
-115.173
1.00
1.01
0 -10.0 -20.0 -30.0 -40.0 -50.0 -60.0 -70.0 -80.0 -90.0 -100.0 -110.0 -120.0 -130.0 -140.0
X : parts per Million : Fluorine19

2,2-Difluoro-3-hydroxy-1-phenyl-3-(*m*-tolyl)propan-1-one (6ak)

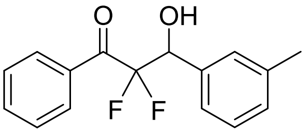

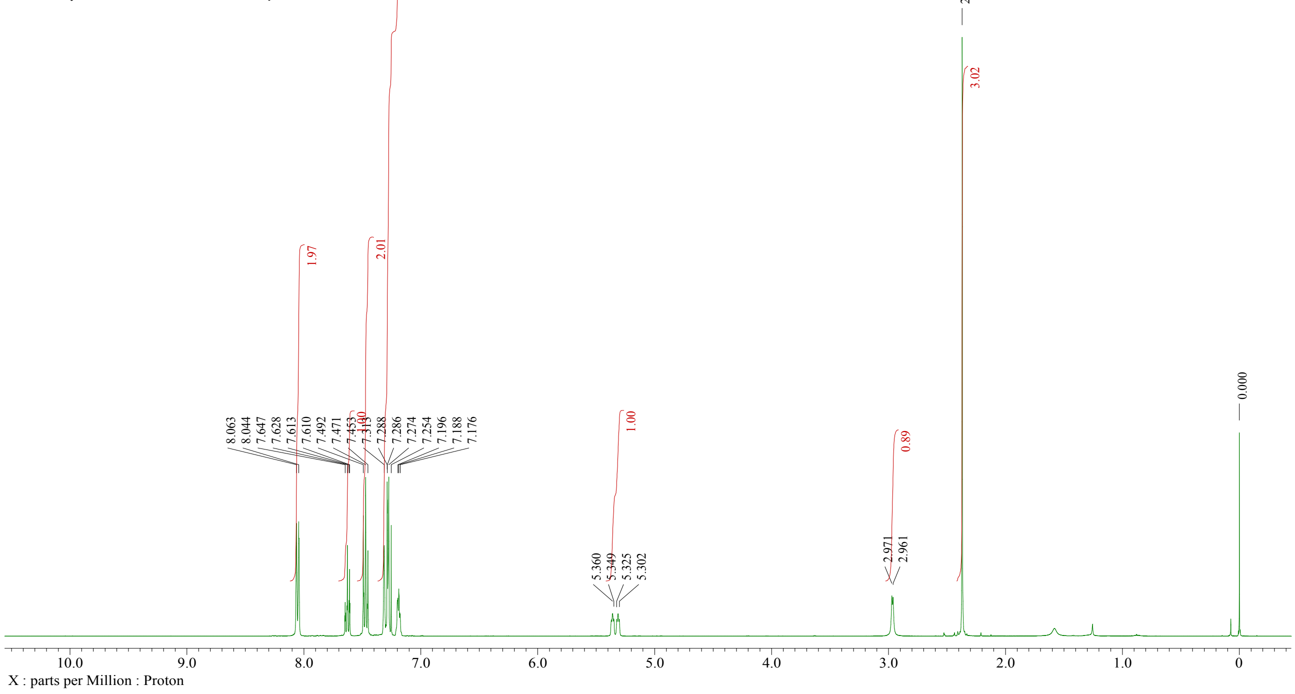

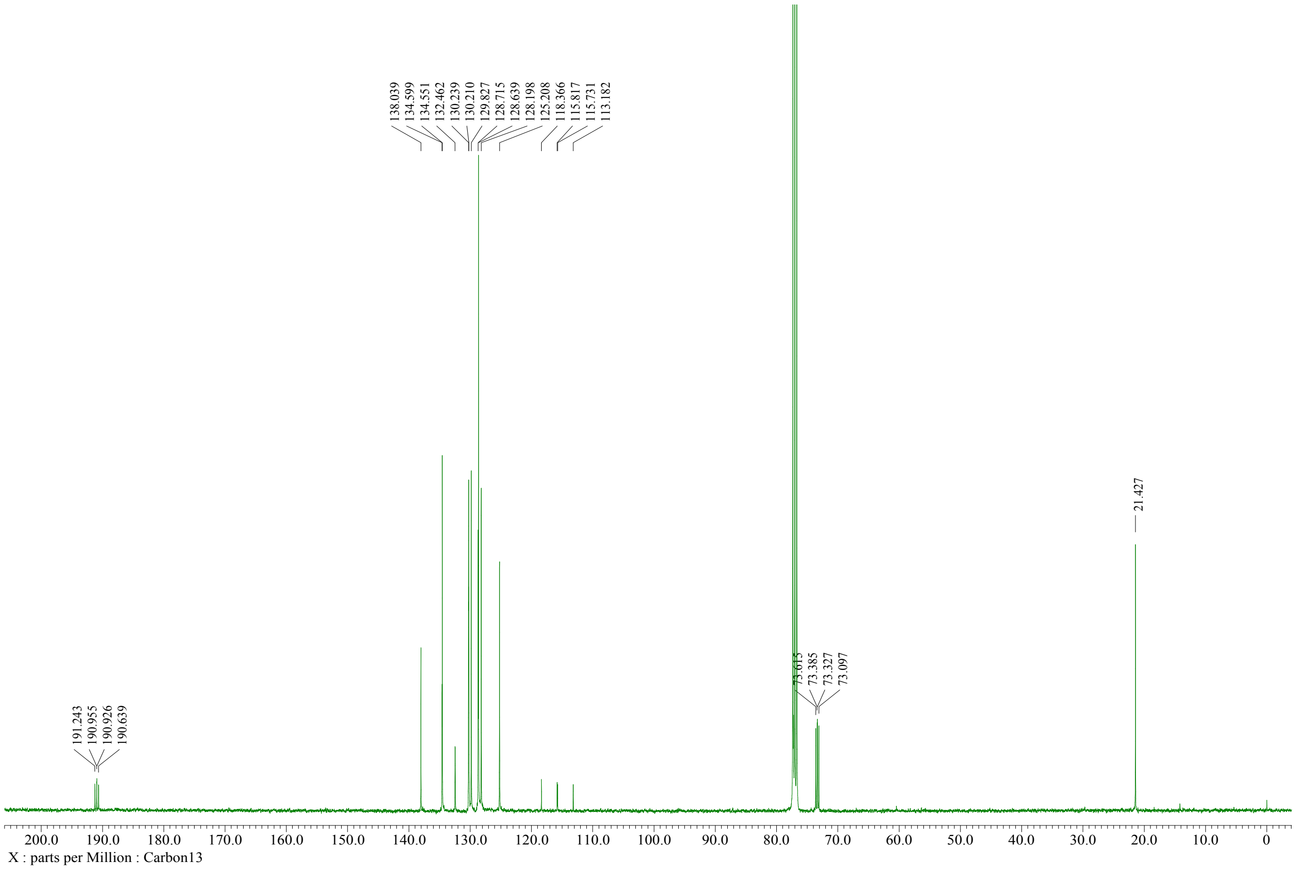

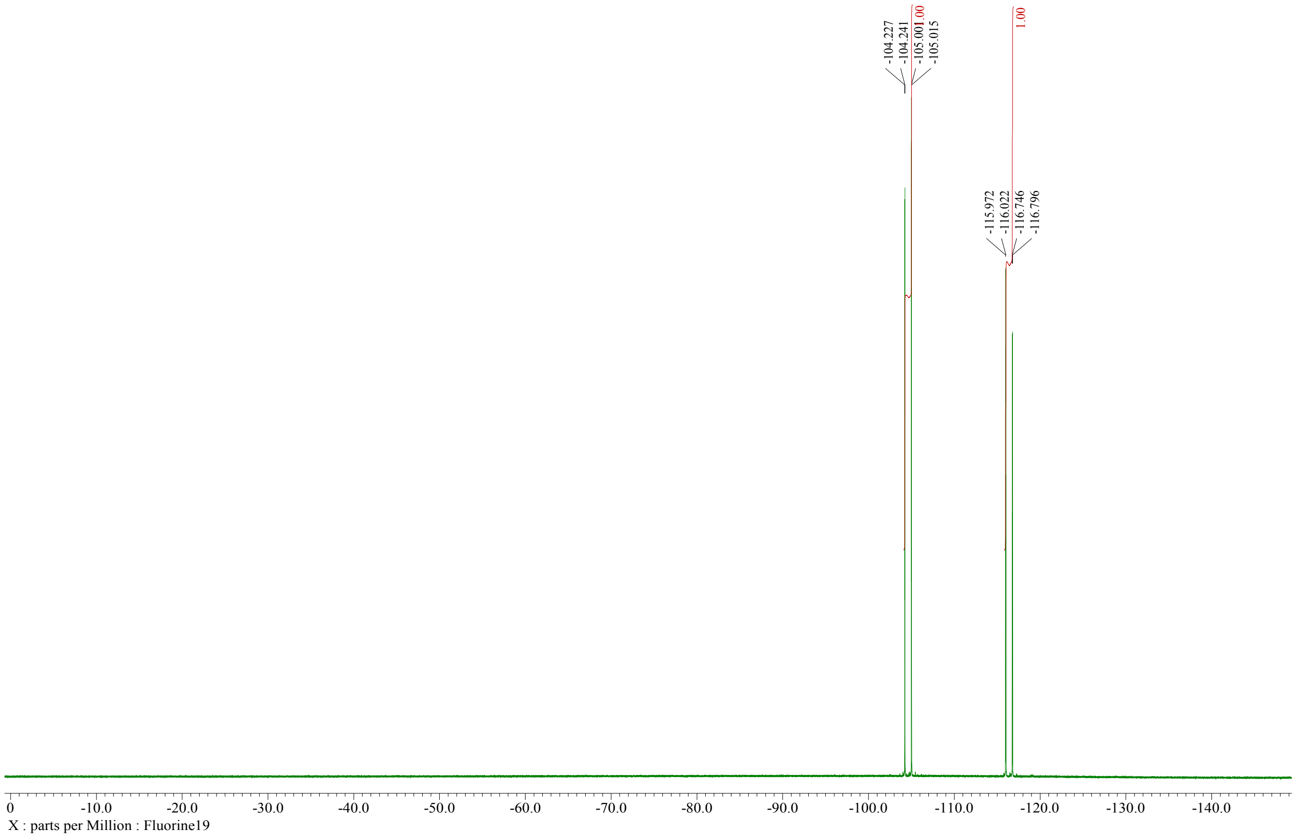

2,2-Difluoro-3-hydroxy-3-(2,4,6-trimethylphenyl)-1-phenylpropan-1-one (6al)

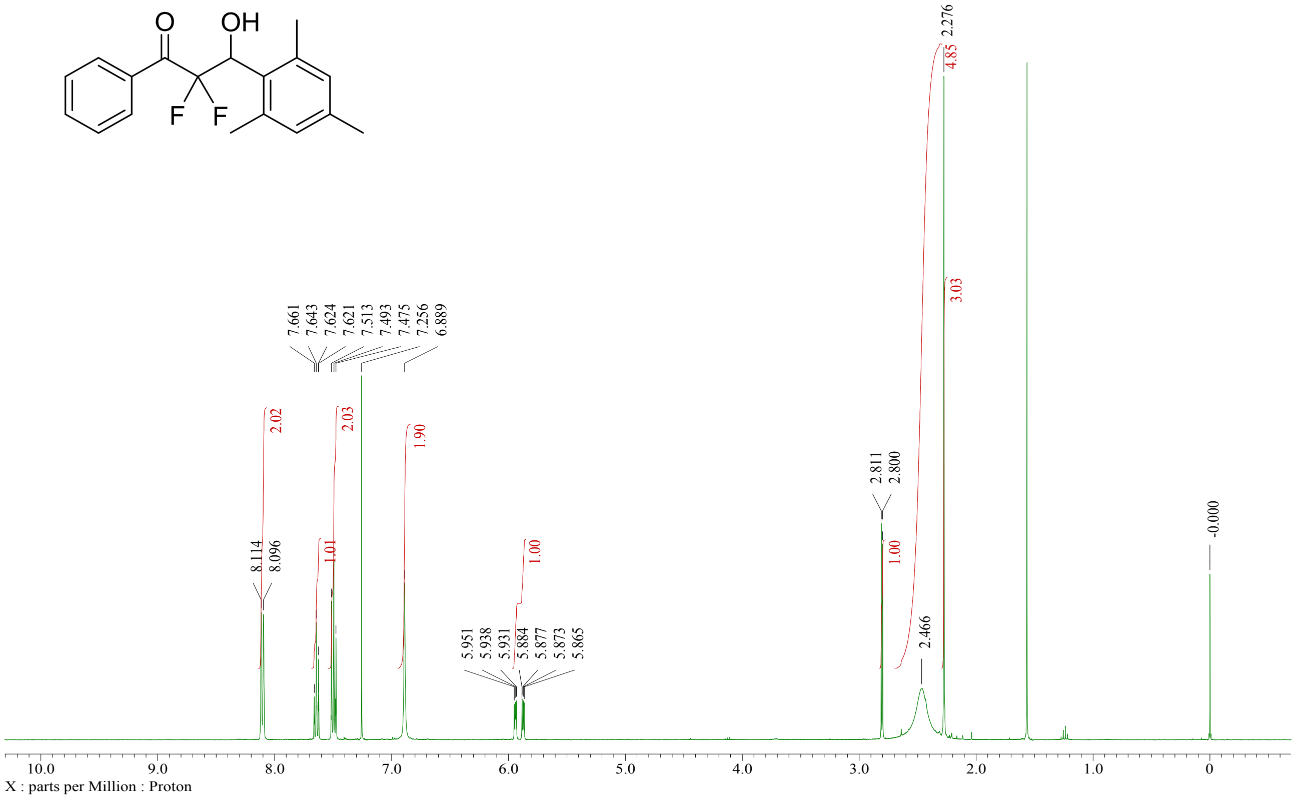

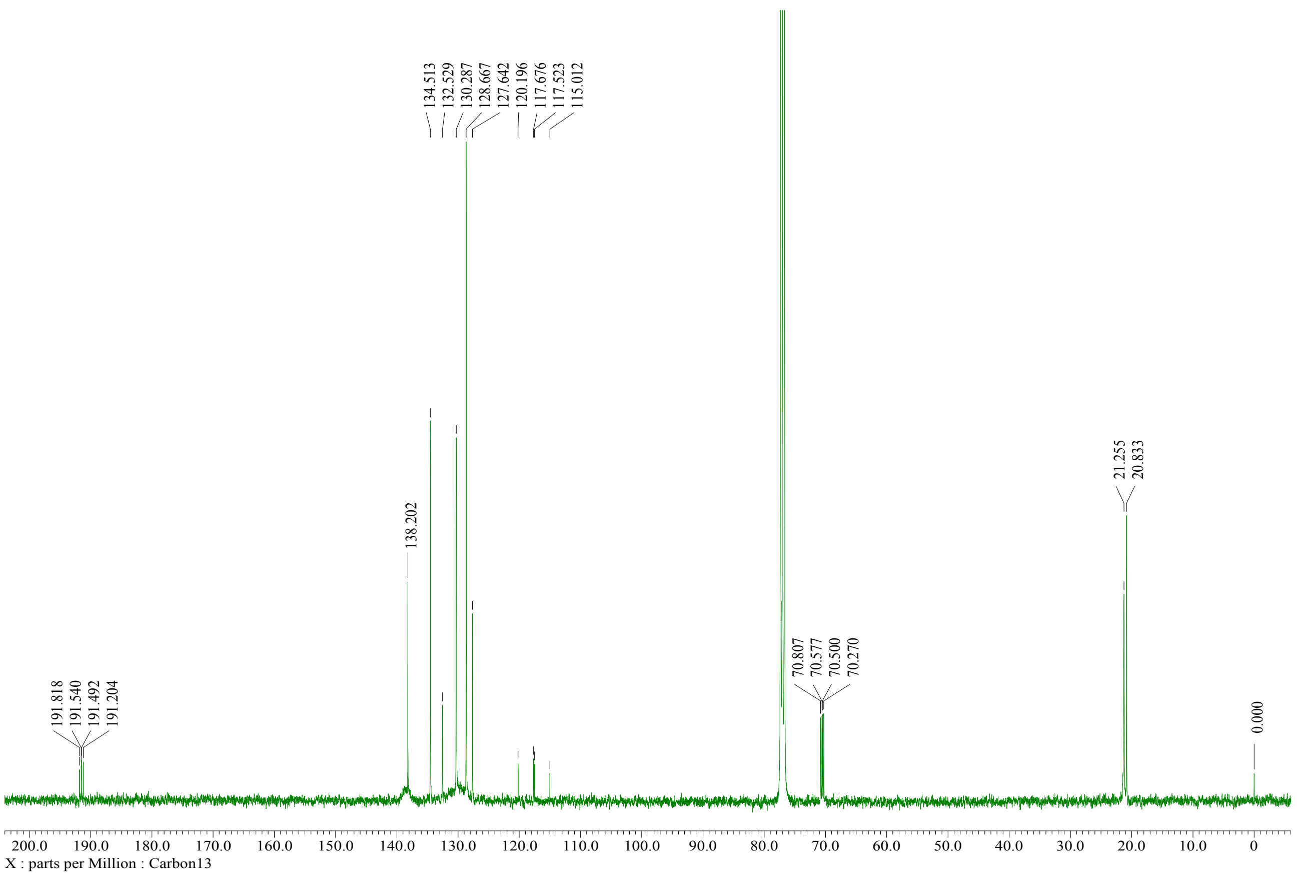

191.818
191.540
191.492
191.204
138.202
134.513
132.529
130.287
128.667
127.642
120.196
117.676
117.523
115.012
70.807
70.577
70.500
70.270
21.255
20.833
0.000
200.0 190.0 180.0 170.0 160.0 150.0 140.0 130.0 120.0 110.0 100.0 90.0 80.0 70.0 60.0 50.0 40.0 30.0 20.0 10.0 0
X : parts per Million : Carbon13

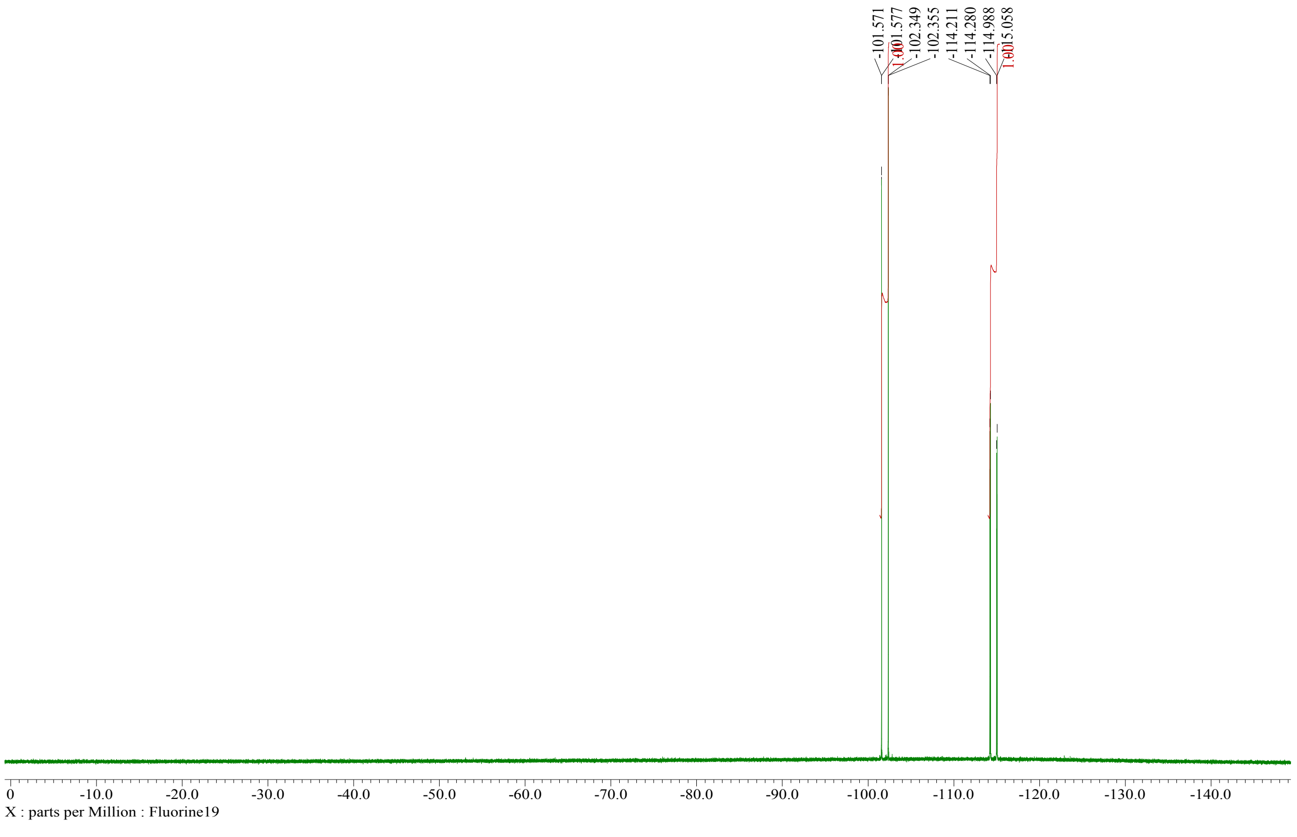

-101.571
-101.577
-102.349
-102.355
-114.211
-114.280
-114.988
-115.058
0 -10.0 -20.0 -30.0 -40.0 -50.0 -60.0 -70.0 -80.0 -90.0 -100.0 -110.0 -120.0 -130.0 -140.0
X : parts per Million : Fluorine19

2,2-Difluoro-3-hydroxy-1-phenyl-3-(pyridin-2-yl)propan-1-one (6am)

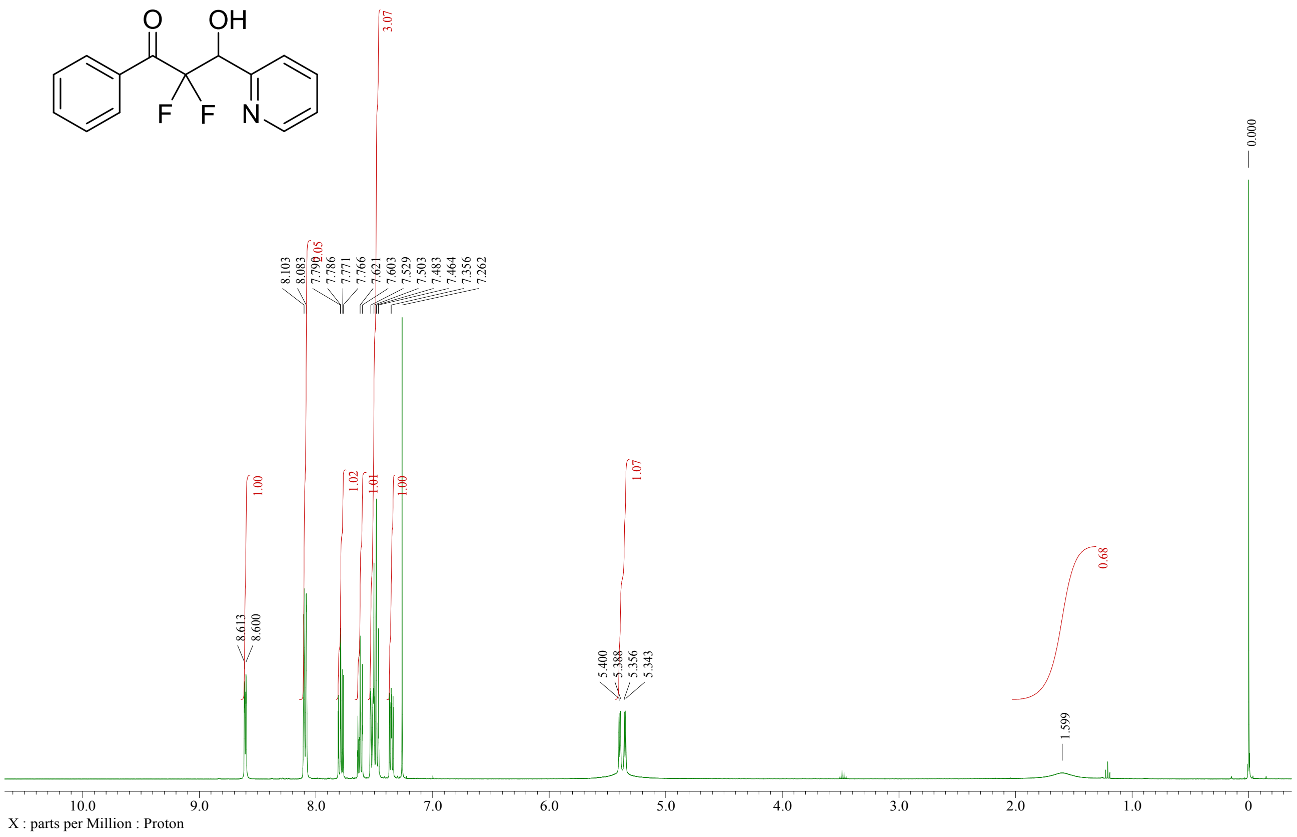

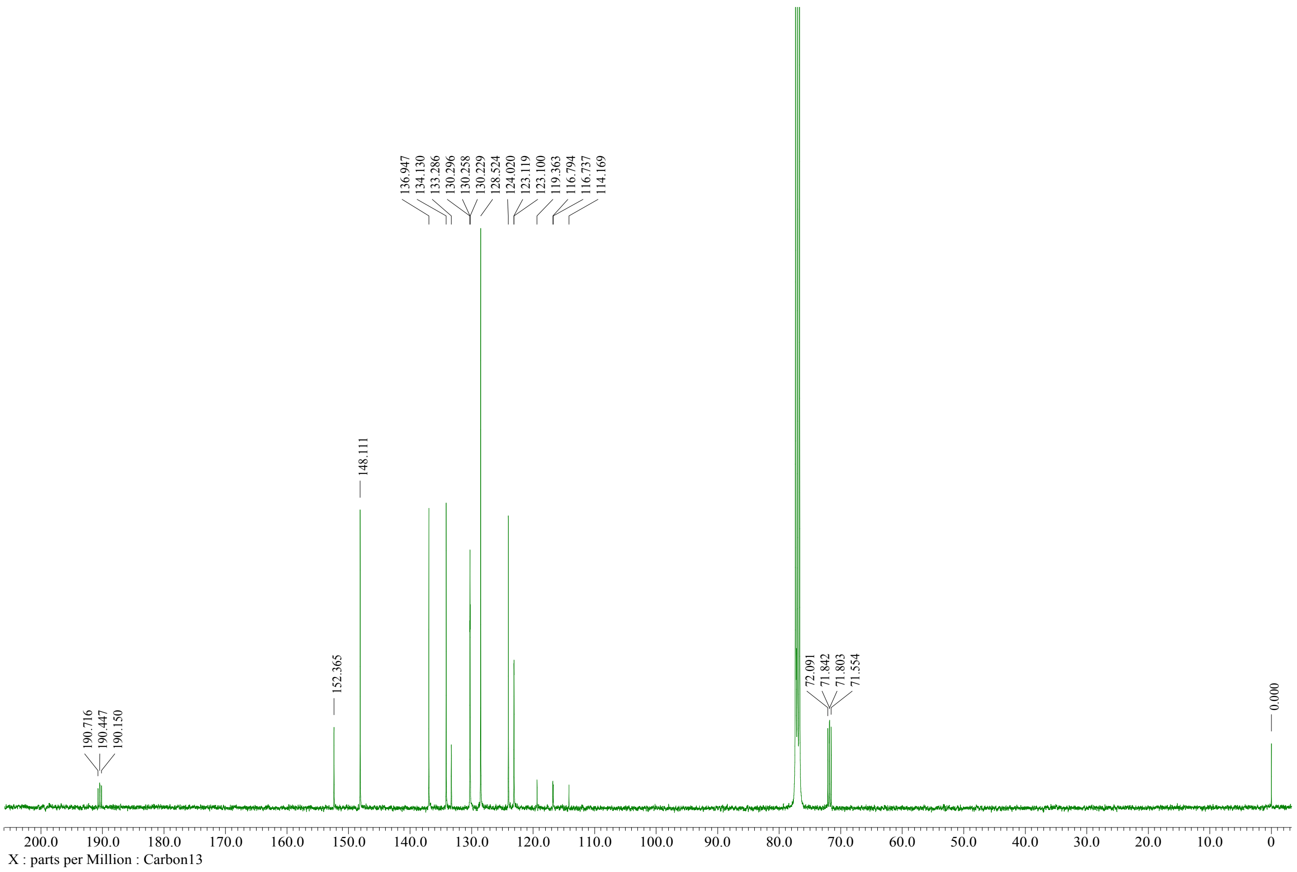

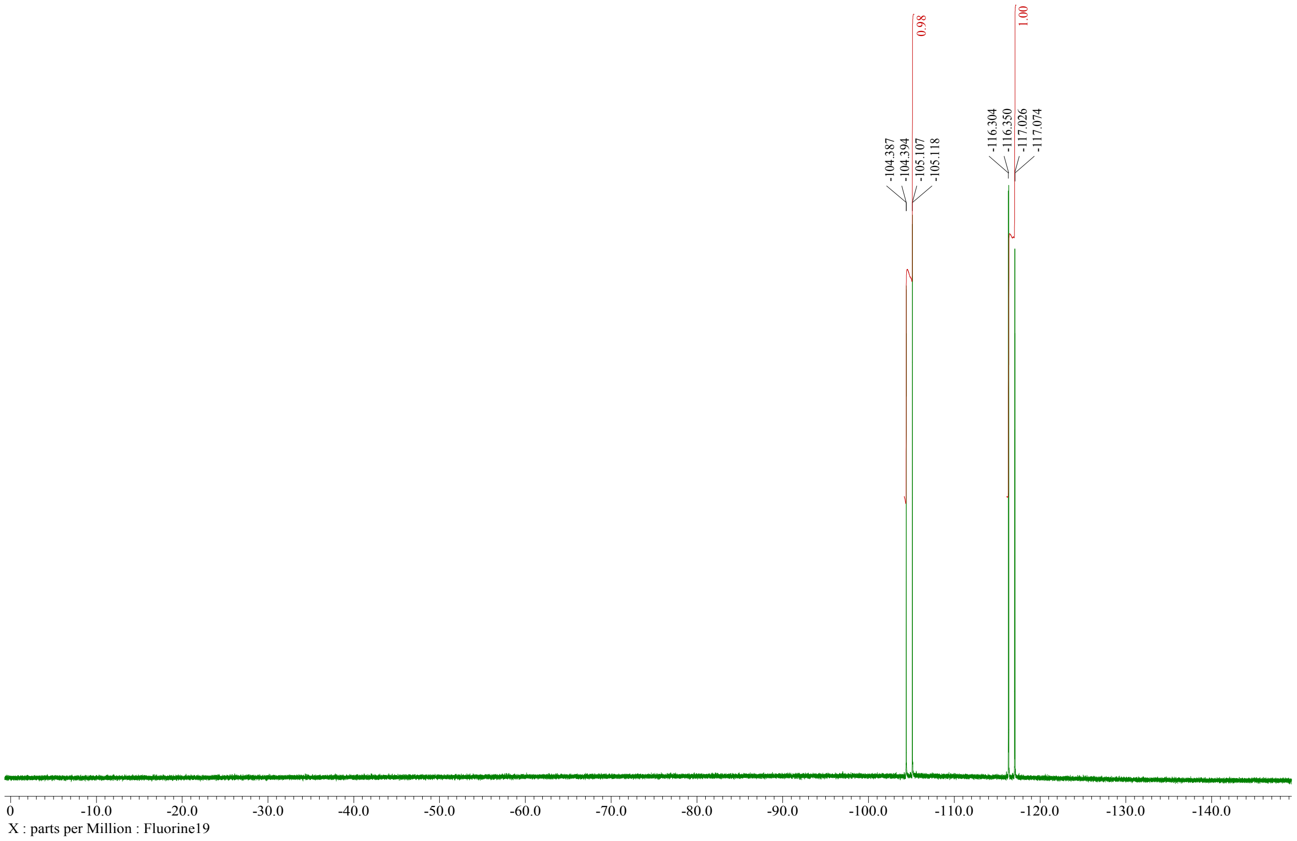

3-Cyclohexyl-2,2-difluoro-3-hydroxy-1-phenylpropan-1-one (6an)

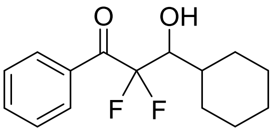

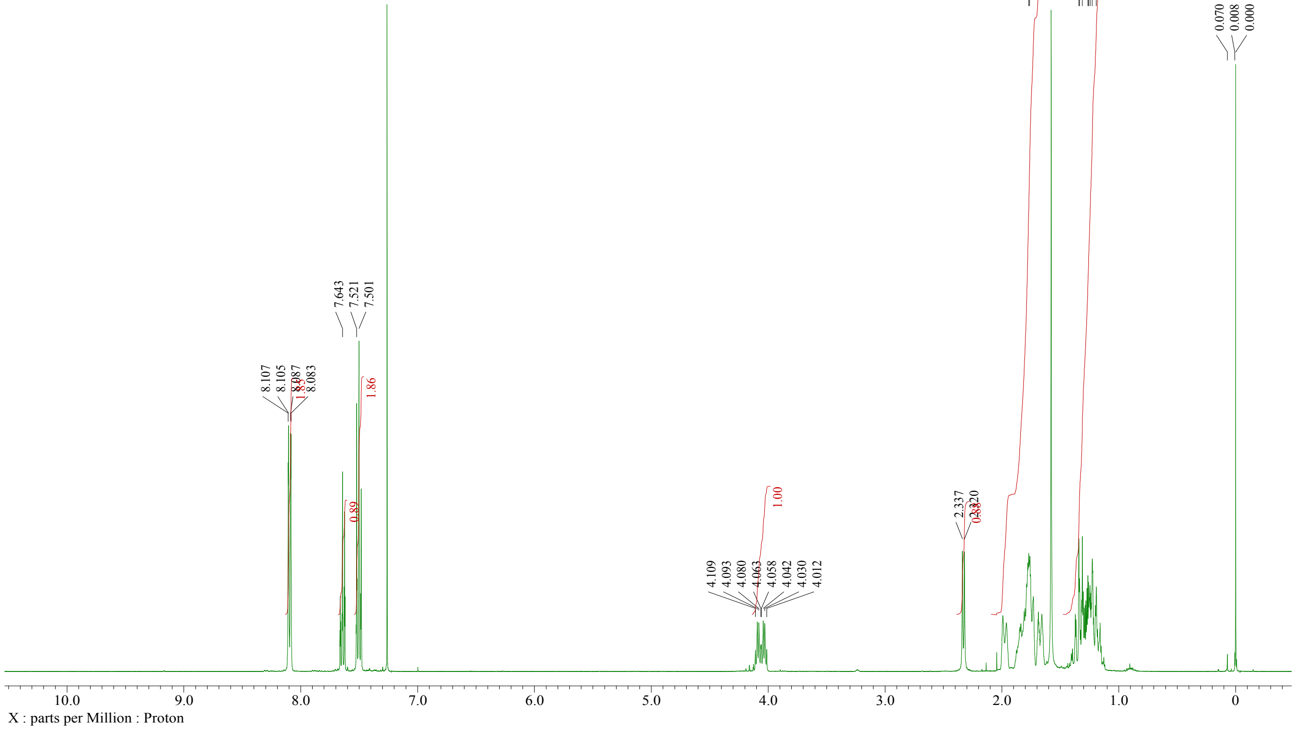

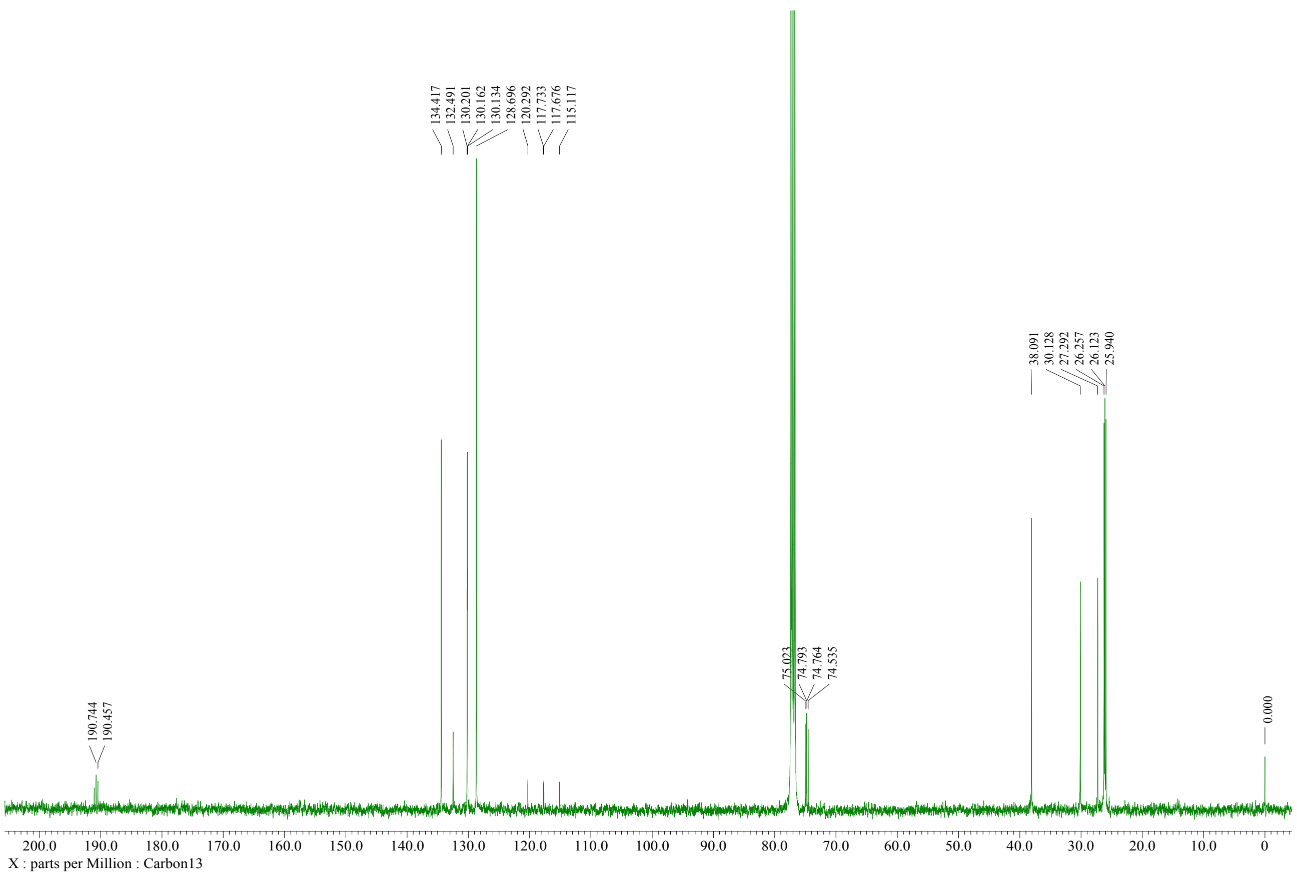

134.417
132.491
130.201
130.162
130.134
128.696
120.292
117.733
117.676
115.117
190.744
190.457
75.023
74.793
74.764
74.535
38.091
30.128
27.292
26.257
26.123
25.940
0.000
200.0 190.0 180.0 170.0 160.0 150.0 140.0 130.0 120.0 110.0 100.0 90.0 80.0 70.0 60.0 50.0 40.0 30.0 20.0 10.0 0
X : parts per Million : Carbon13

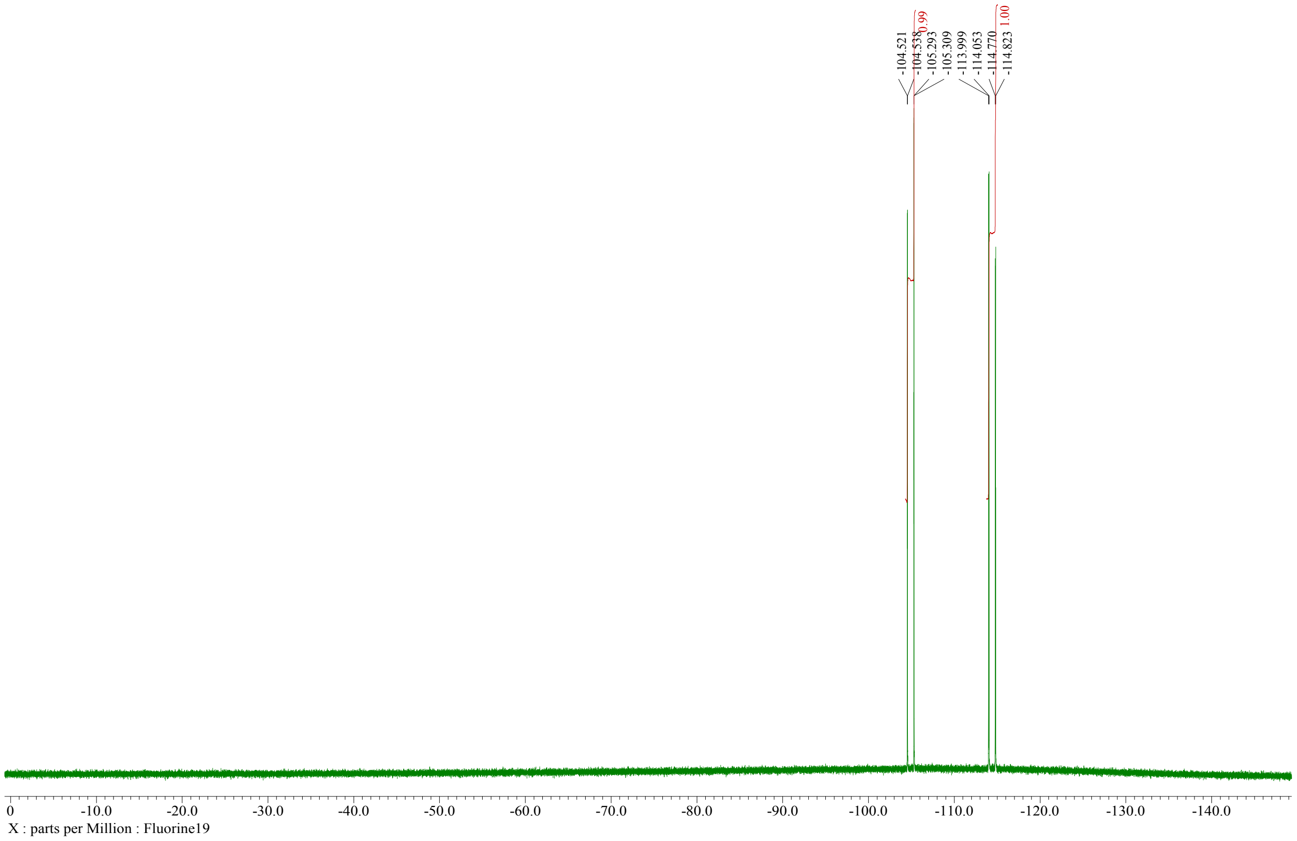

-104.521
-104.538
-105.293
-105.309
-113.999
-114.053
-114.770
-114.823
0.99
1.00
0 -10.0 -20.0 -30.0 -40.0 -50.0 -60.0 -70.0 -80.0 -90.0 -100.0 -110.0 -120.0 -130.0 -140.0
X : parts per Million : Fluorine19

2,2-Difluoro-3-hydroxy-1,5-diphenylpentan-1-one (6ao)

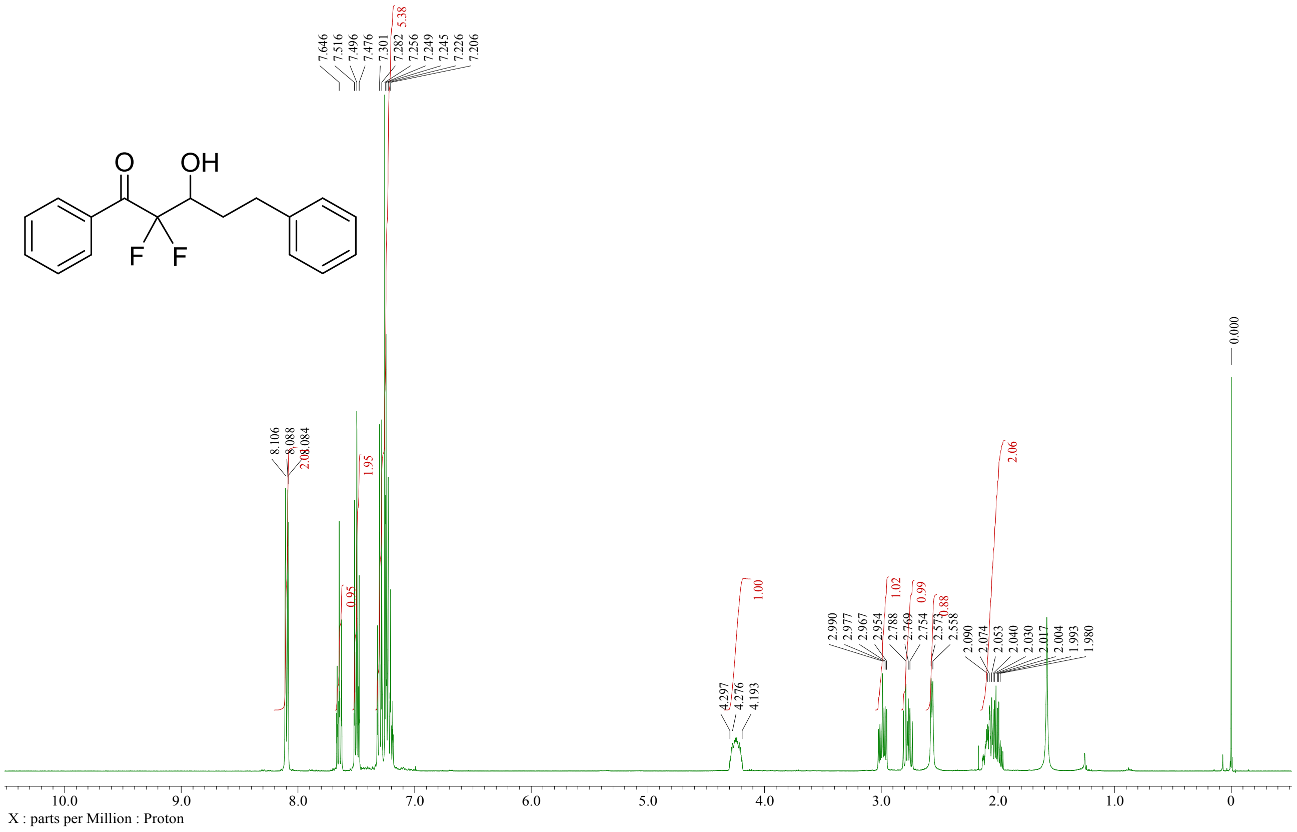

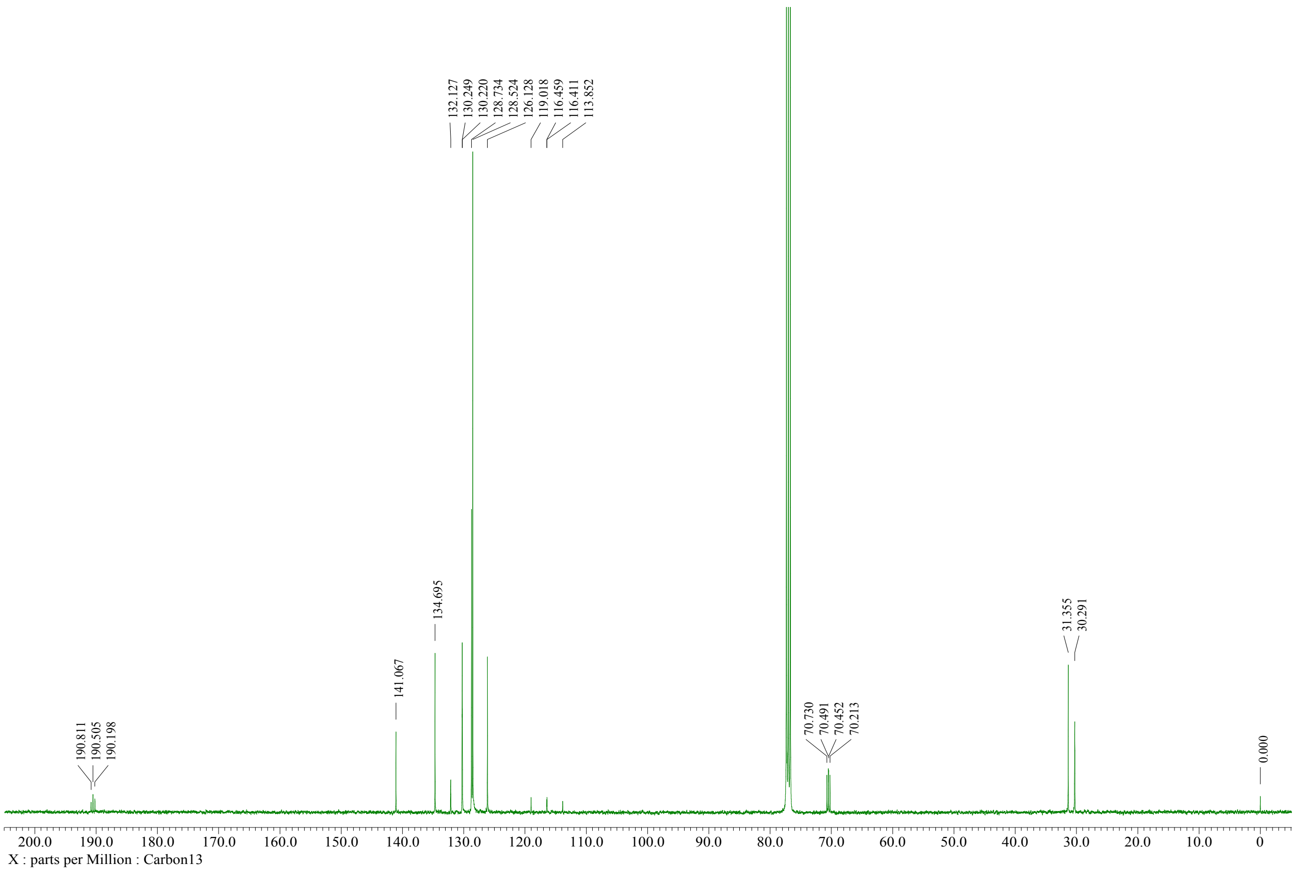

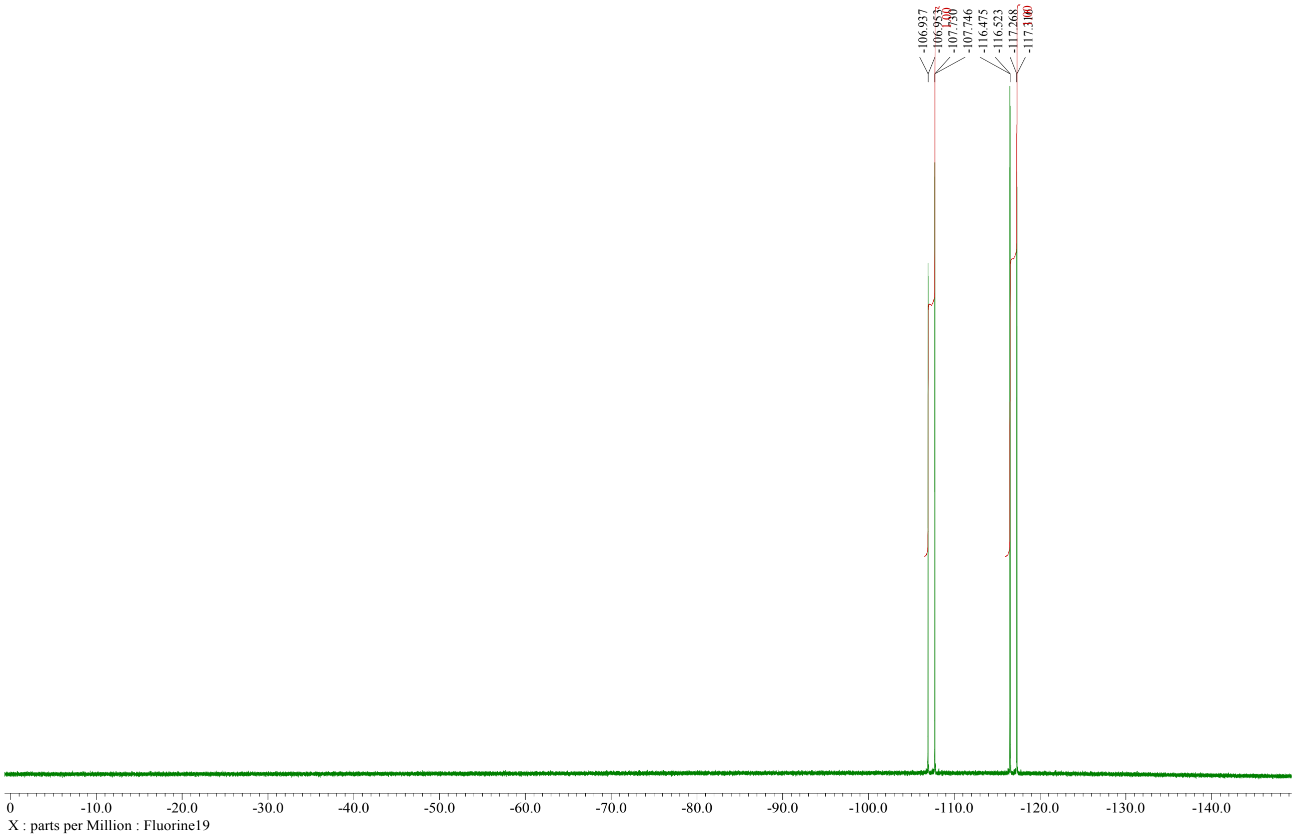

2,2-Difluoro-3-hydroxy-1-(4-methoxyphenyl)-3-phenylpropan-1-one (6ba)

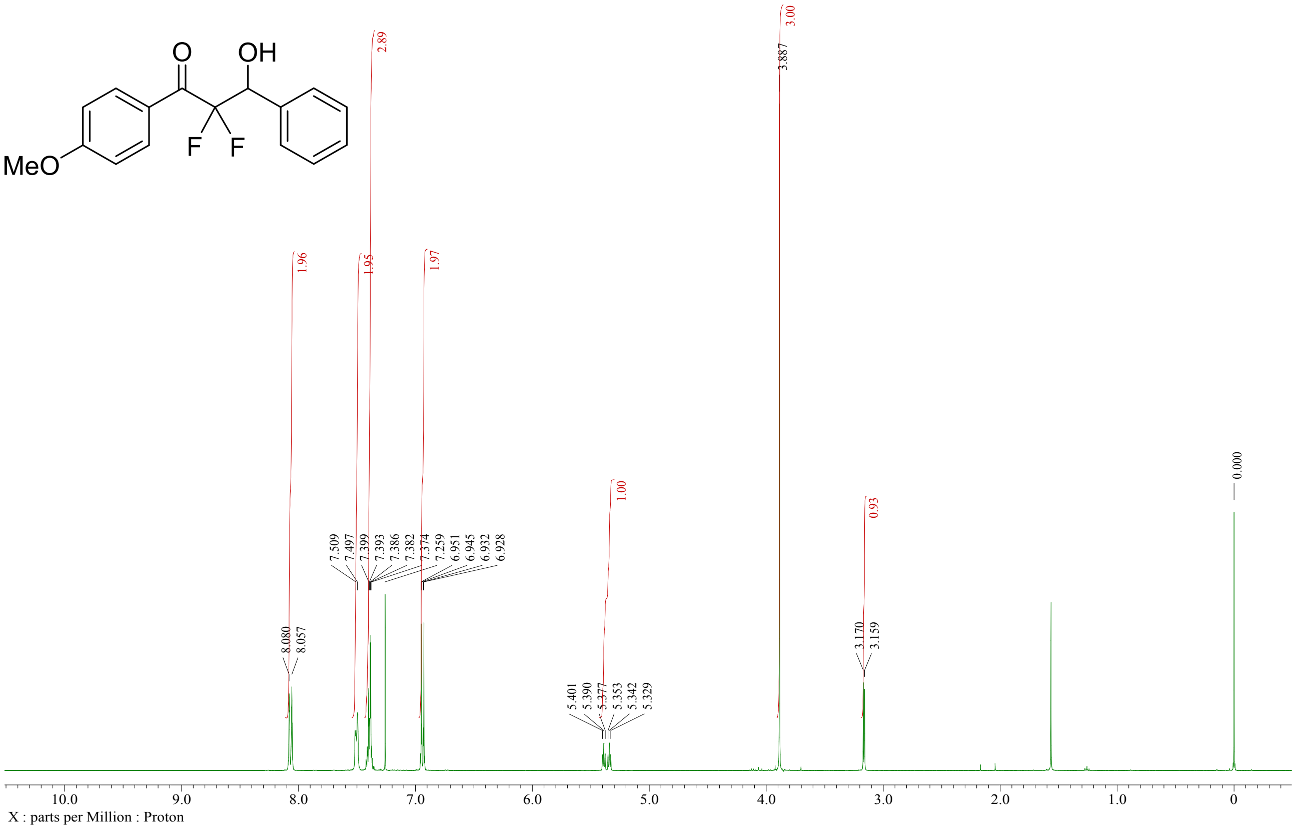

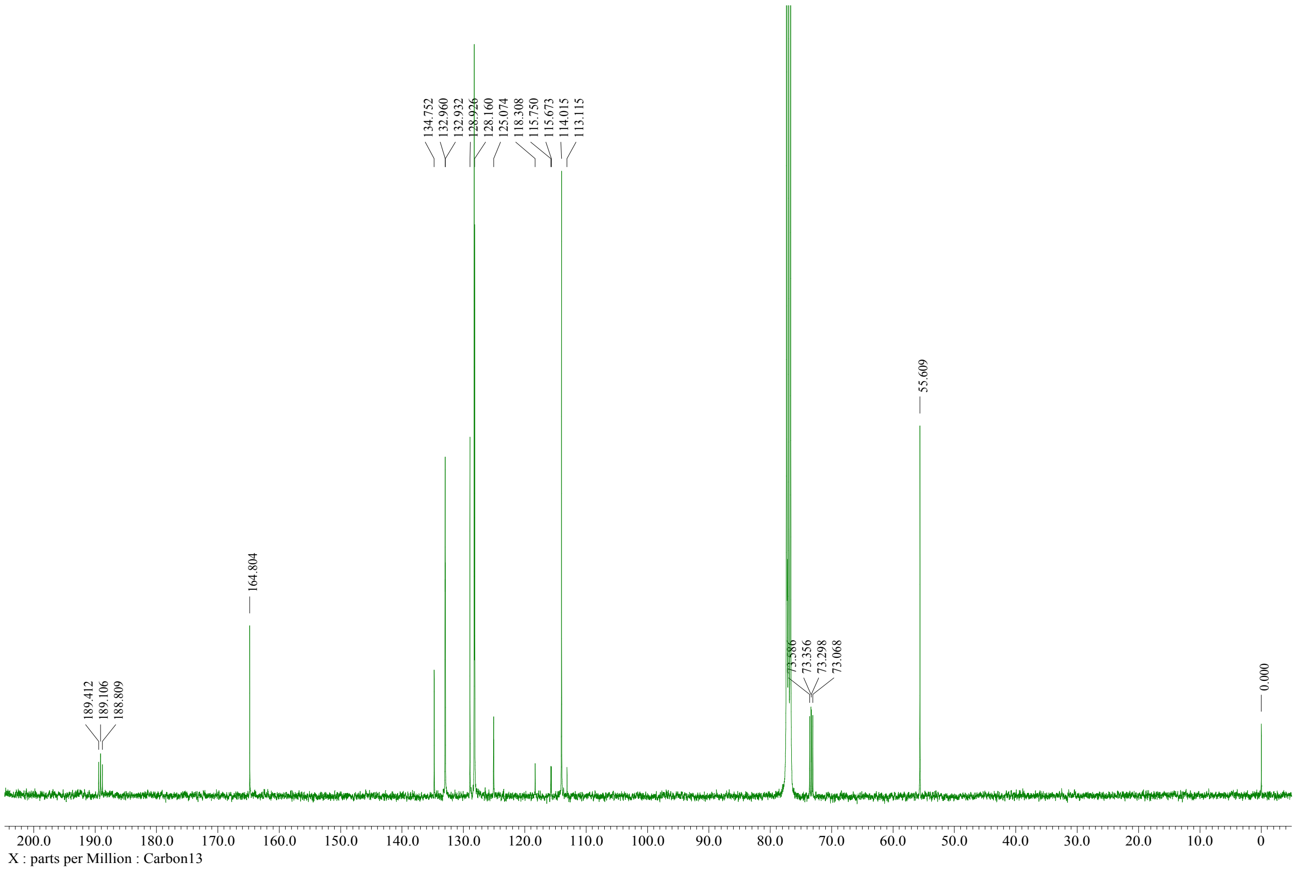

189.412
189.106
188.809
164.804
134.752
132.960
132.932
128.926
128.160
125.074
118.308
115.750
115.673
114.015
113.115
73.586
73.356
73.298
73.068
55.609
0.000
200.0 190.0 180.0 170.0 160.0 150.0 140.0 130.0 120.0 110.0 100.0 90.0 80.0 70.0 60.0 50.0 40.0 30.0 20.0 10.0 0
X : parts per Million : Carbon13

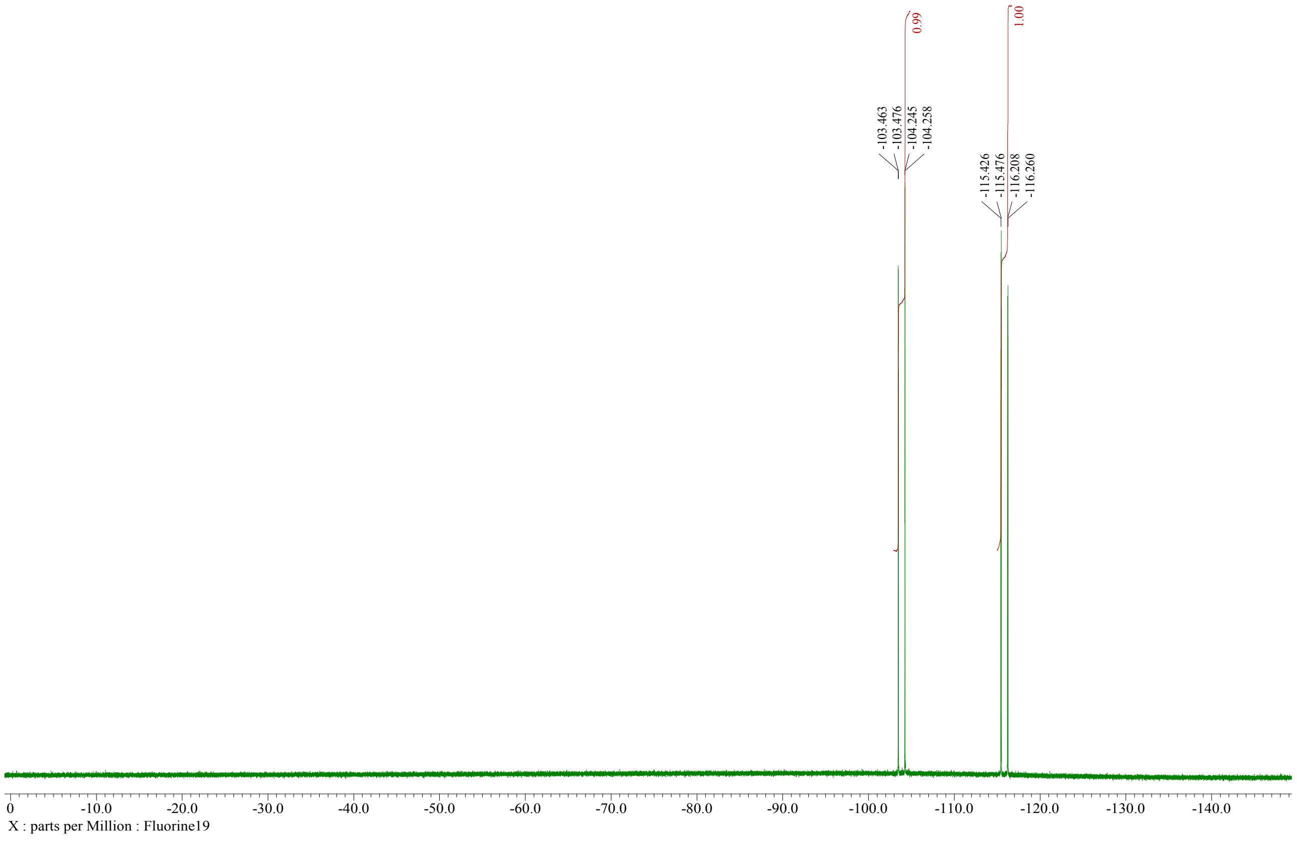

0.99
1.00
-103.463
-103.476
-104.245
-104.258
-115.426
-115.476
-116.208
-116.260
0 -10.0 -20.0 -30.0 -40.0 -50.0 -60.0 -70.0 -80.0 -90.0 -100.0 -110.0 -120.0 -130.0 -140.0
X : parts per Million : Fluorine19

1-(4-Chlorophenyl)-2,2-difluoro-3-hydroxy-3-phenylpropan-1-one (6ca)

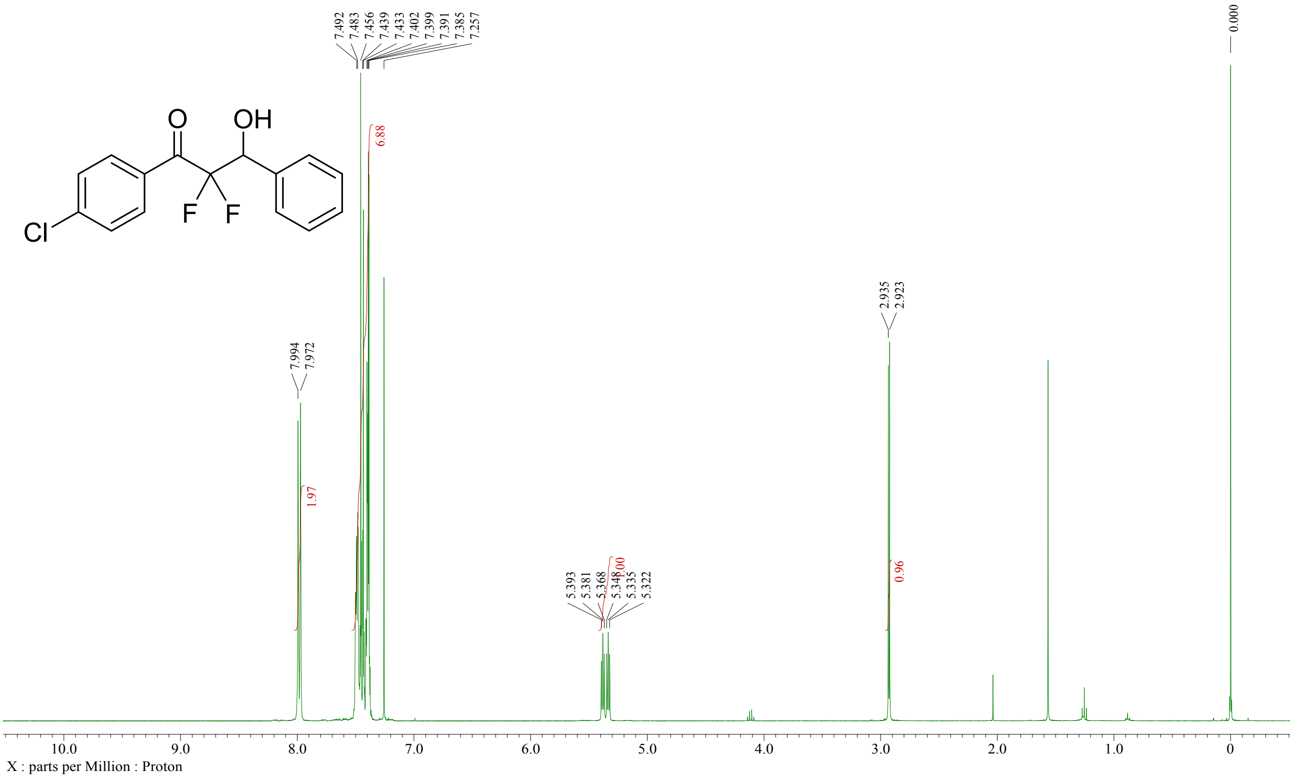

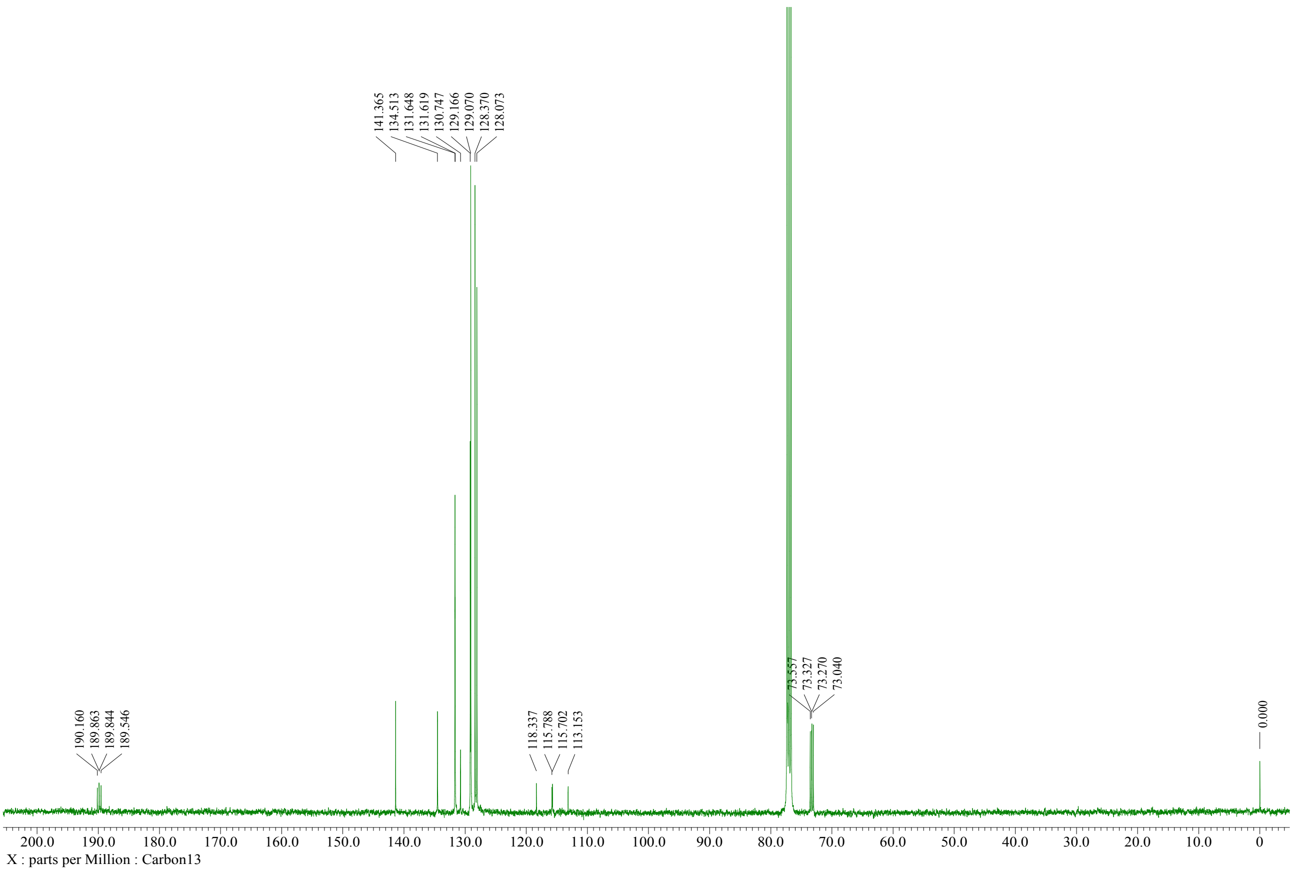

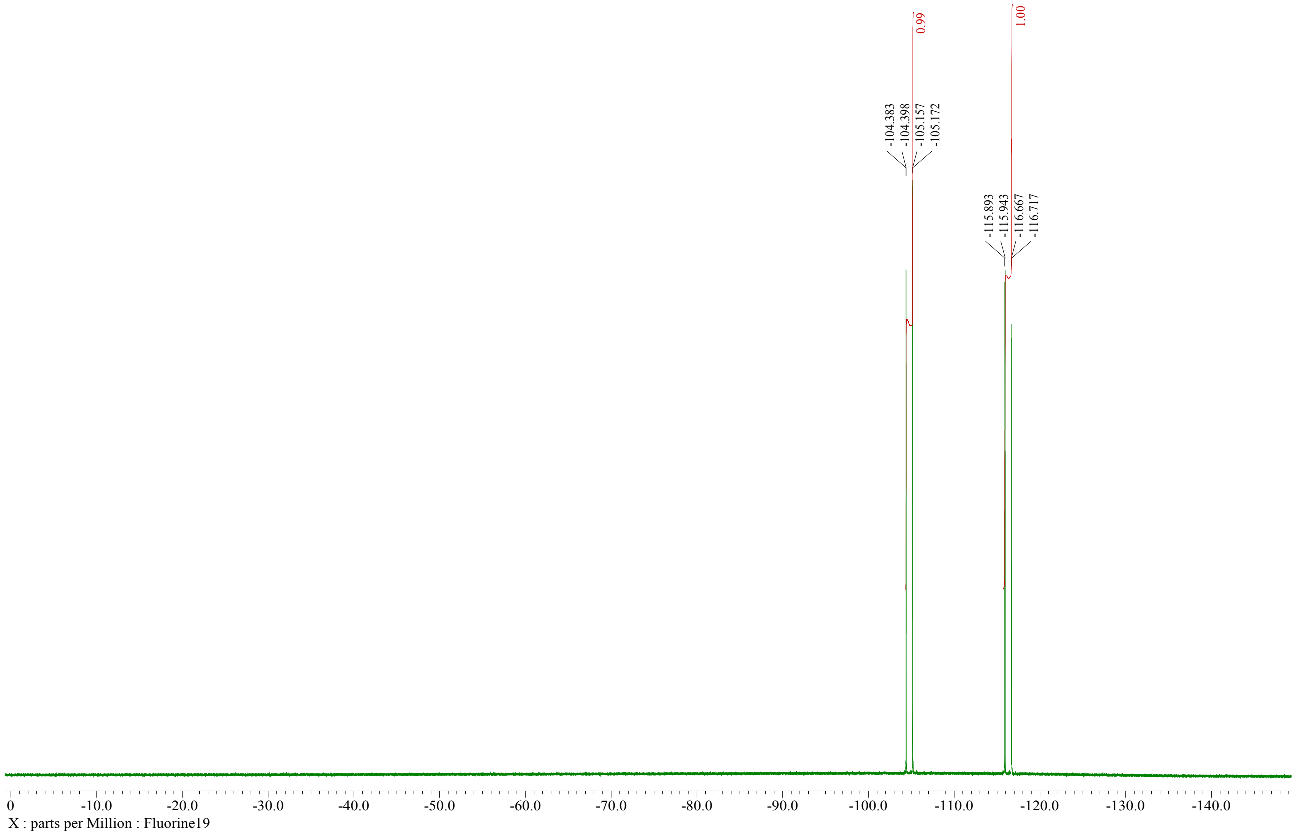

1-Cyclohexyl-2,2-difluoro-3-hydroxy-3-phenylpropan-1-one (6da)

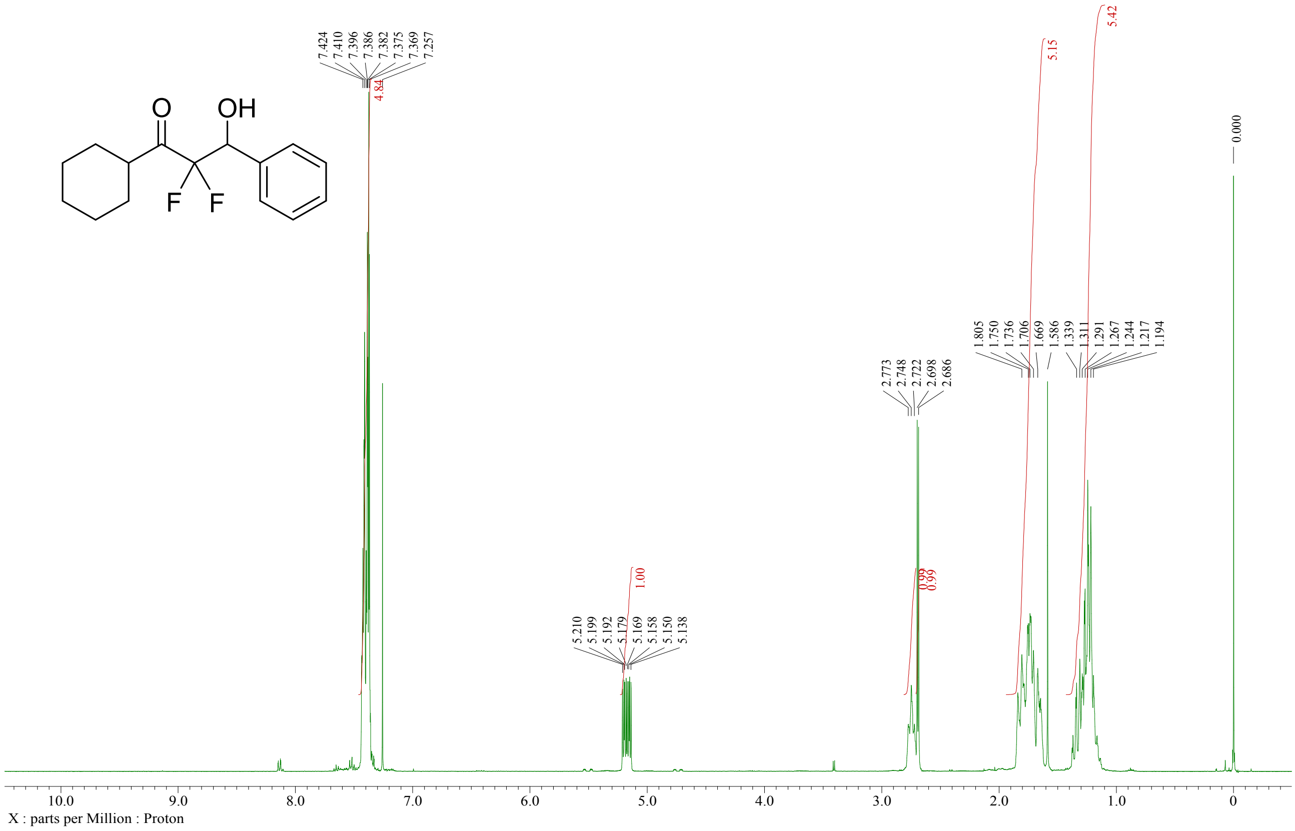

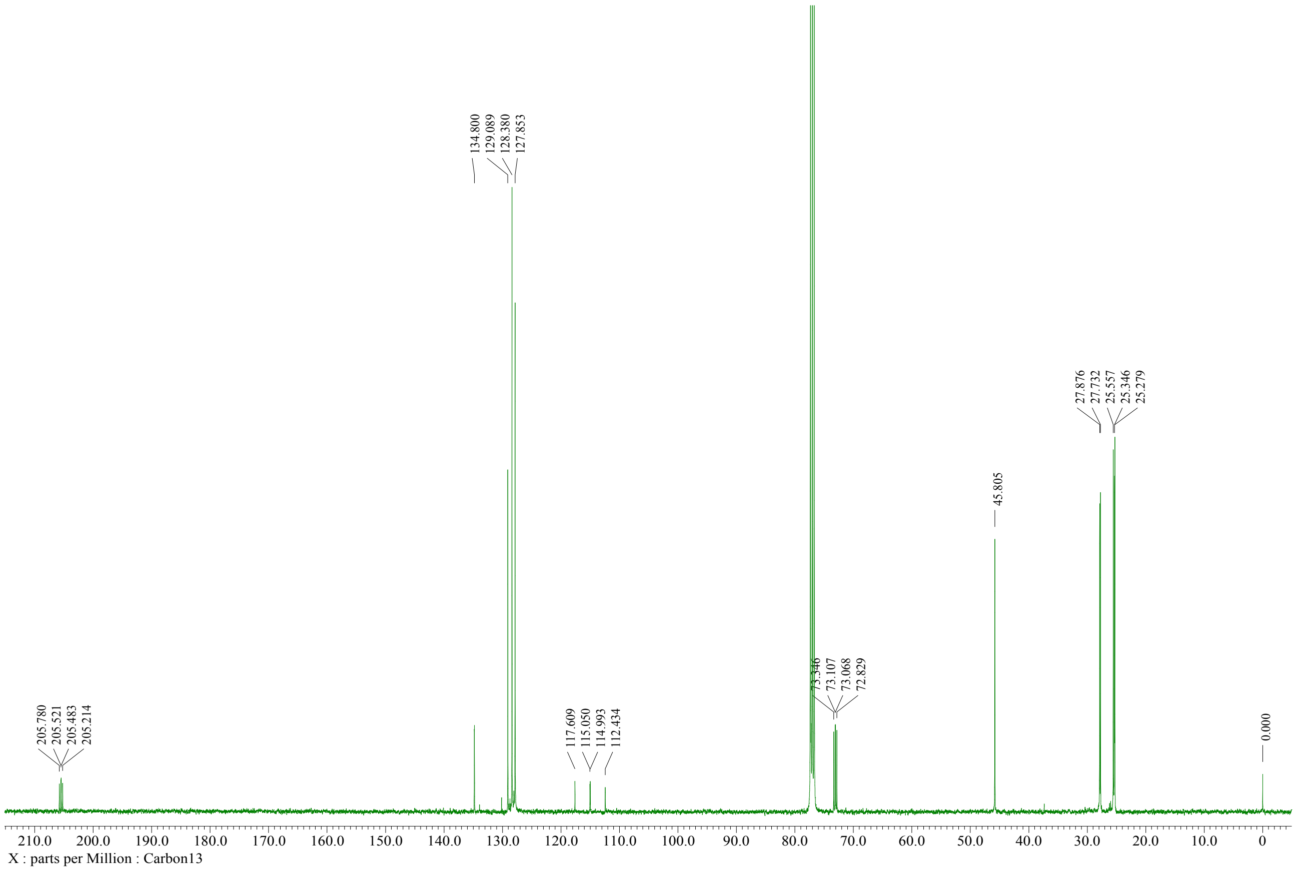
205.780
205.521
205.483
205.214
134.800
129.089
128.380
127.853
117.609
115.050
114.993
112.434
73.346
73.107
73.068
72.829
45.805
27.876
27.732
25.557
25.346
25.279
0.000
210.0 200.0 190.0 180.0 170.0 160.0 150.0 140.0 130.0 120.0 110.0 100.0 90.0 80.0 70.0 60.0 50.0 40.0 30.0 20.0 10.0 0
X : parts per Million : Carbon13

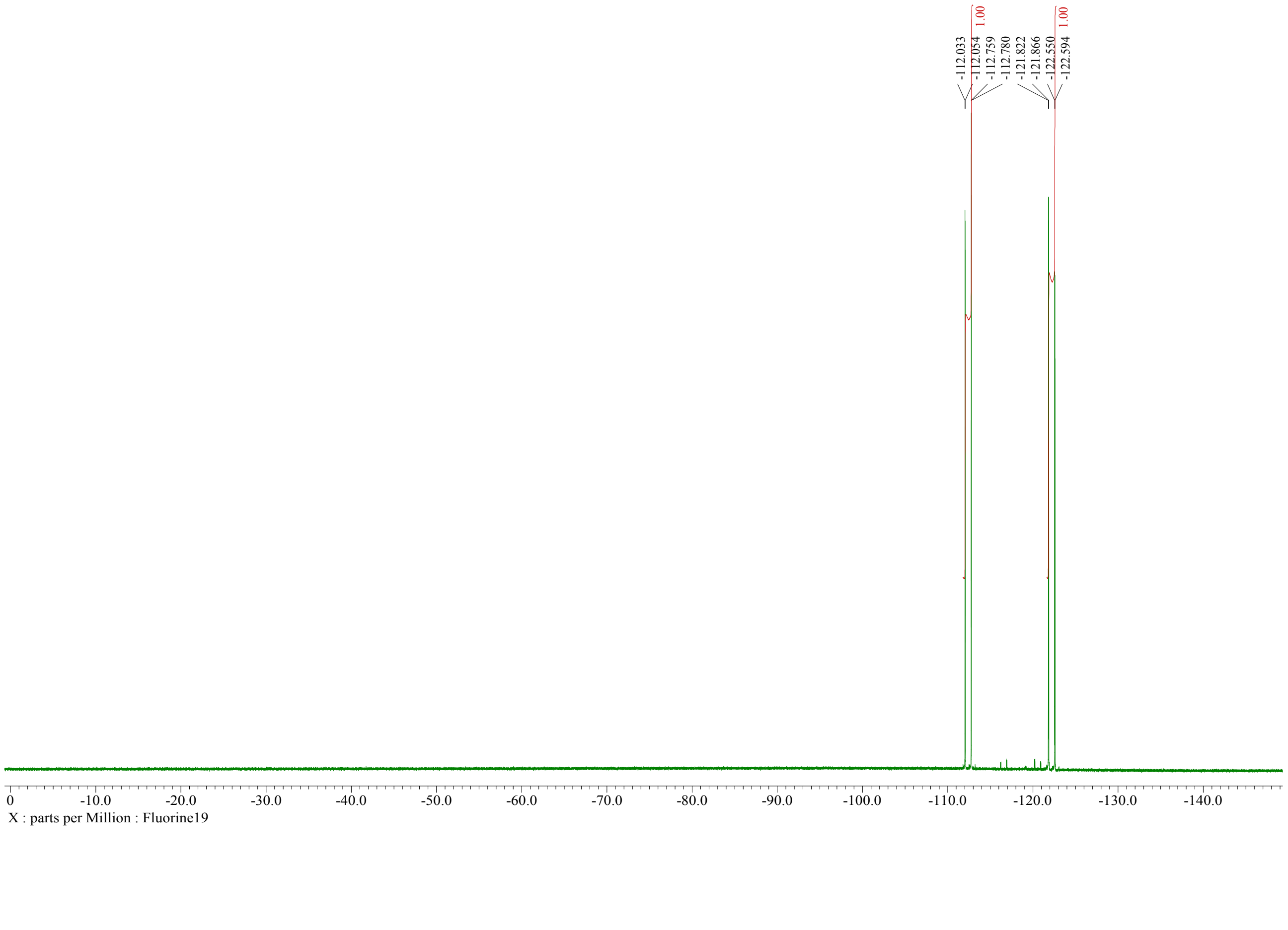
1.00
1.00
-112.033
-112.054
-112.759
-112.780
-121.822
-121.866
-122.550
-122.594
0 -10.0 -20.0 -30.0 -40.0 -50.0 -60.0 -70.0 -80.0 -90.0 -100.0 -110.0 -120.0 -130.0 -140.0
X : parts per Million : Fluorine19

2,2-Difluoro-3-hydroxy-1,3-diphenylbutan-1-on (6ap)

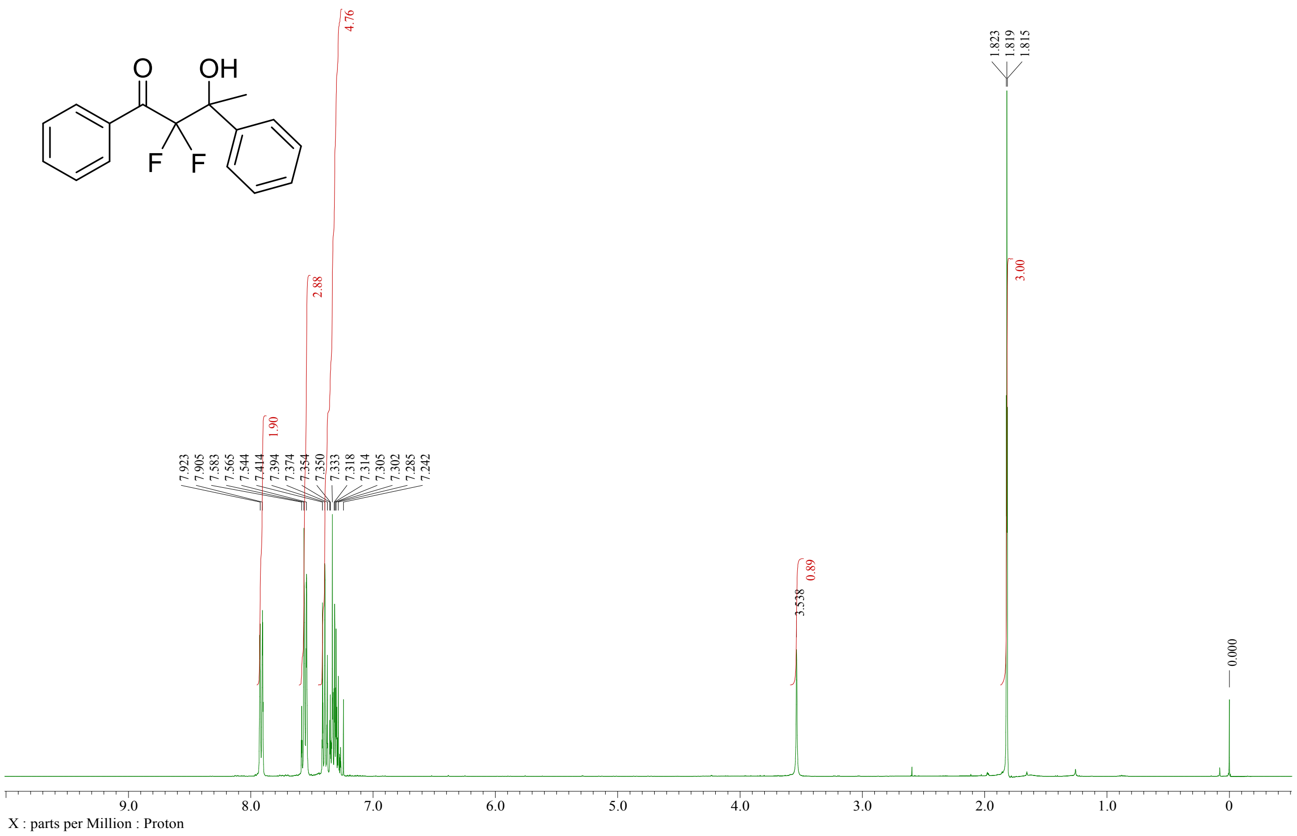

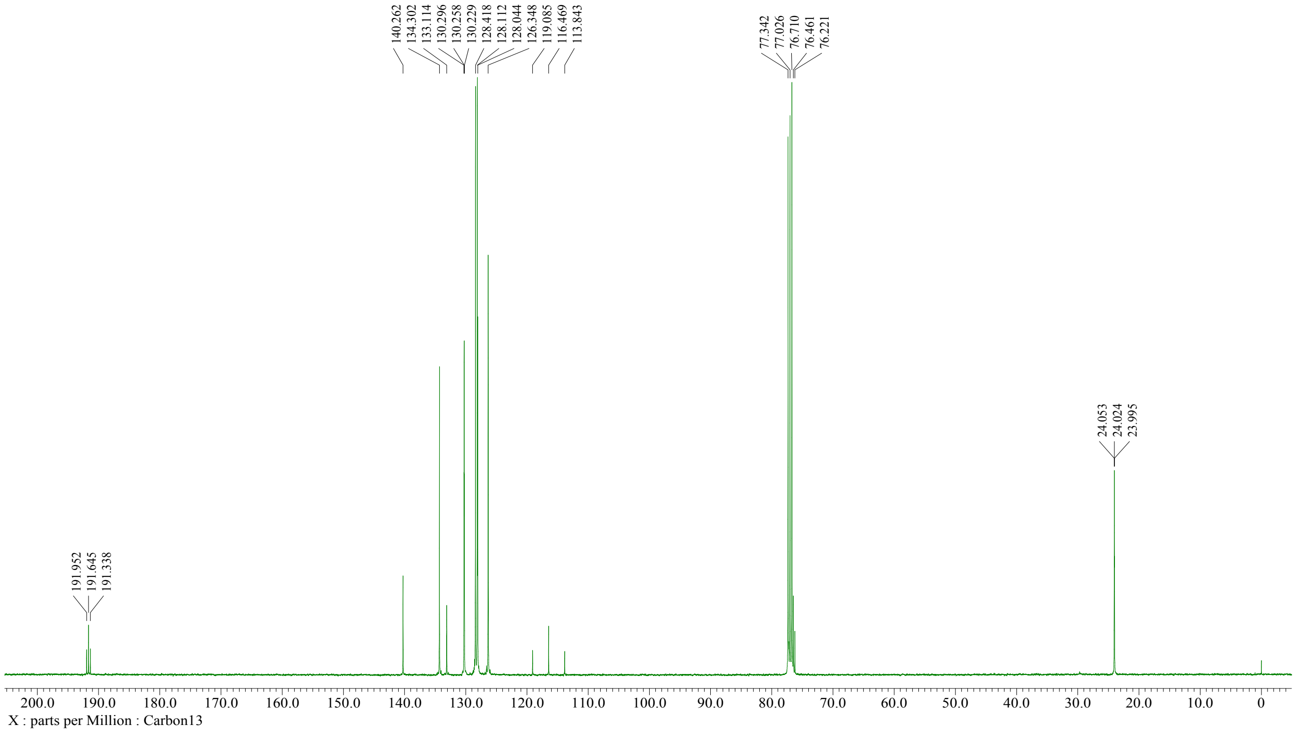

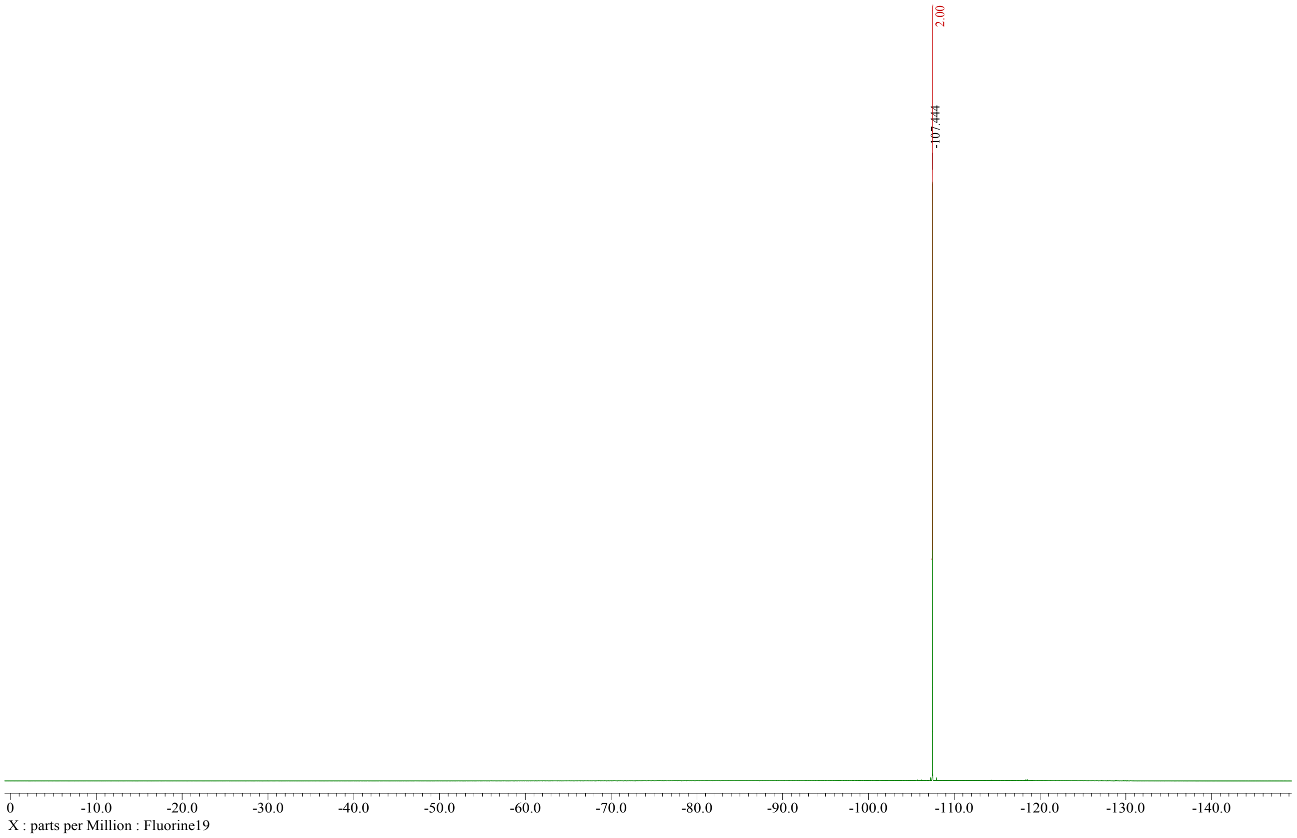

2,2-Difluoro-3-hydroxy-3-methyl-1-phenylbutan-1-one (6aq)

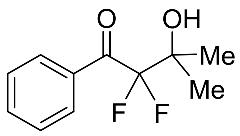

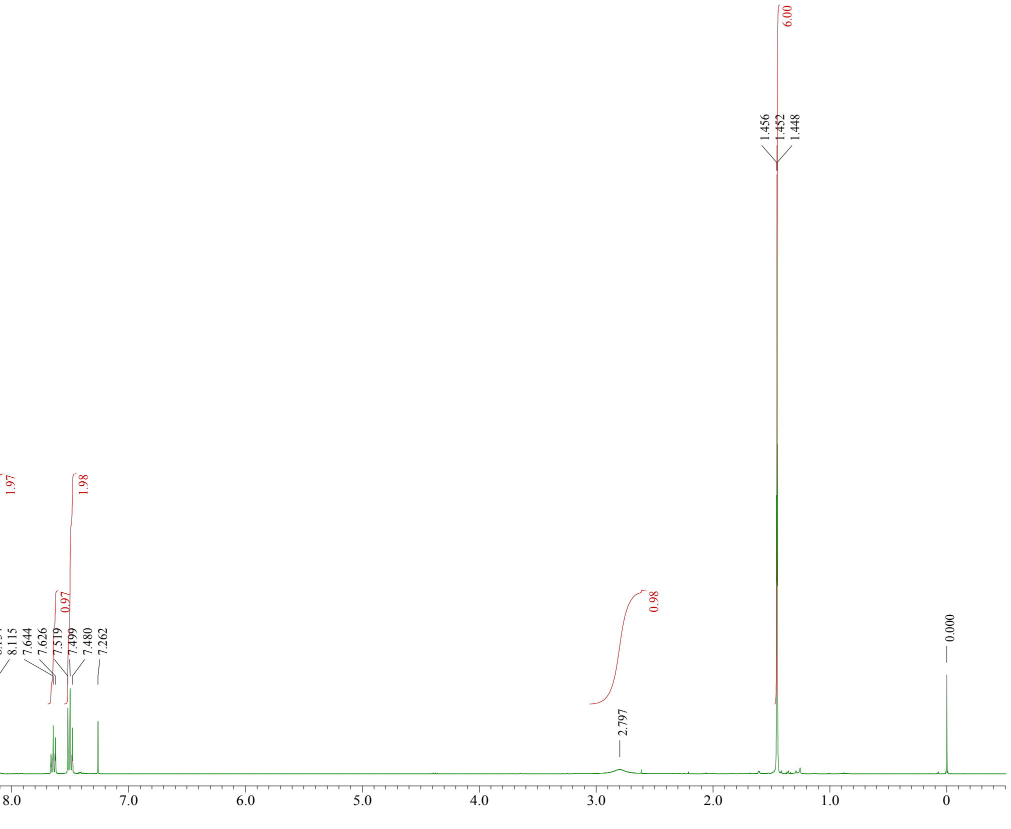

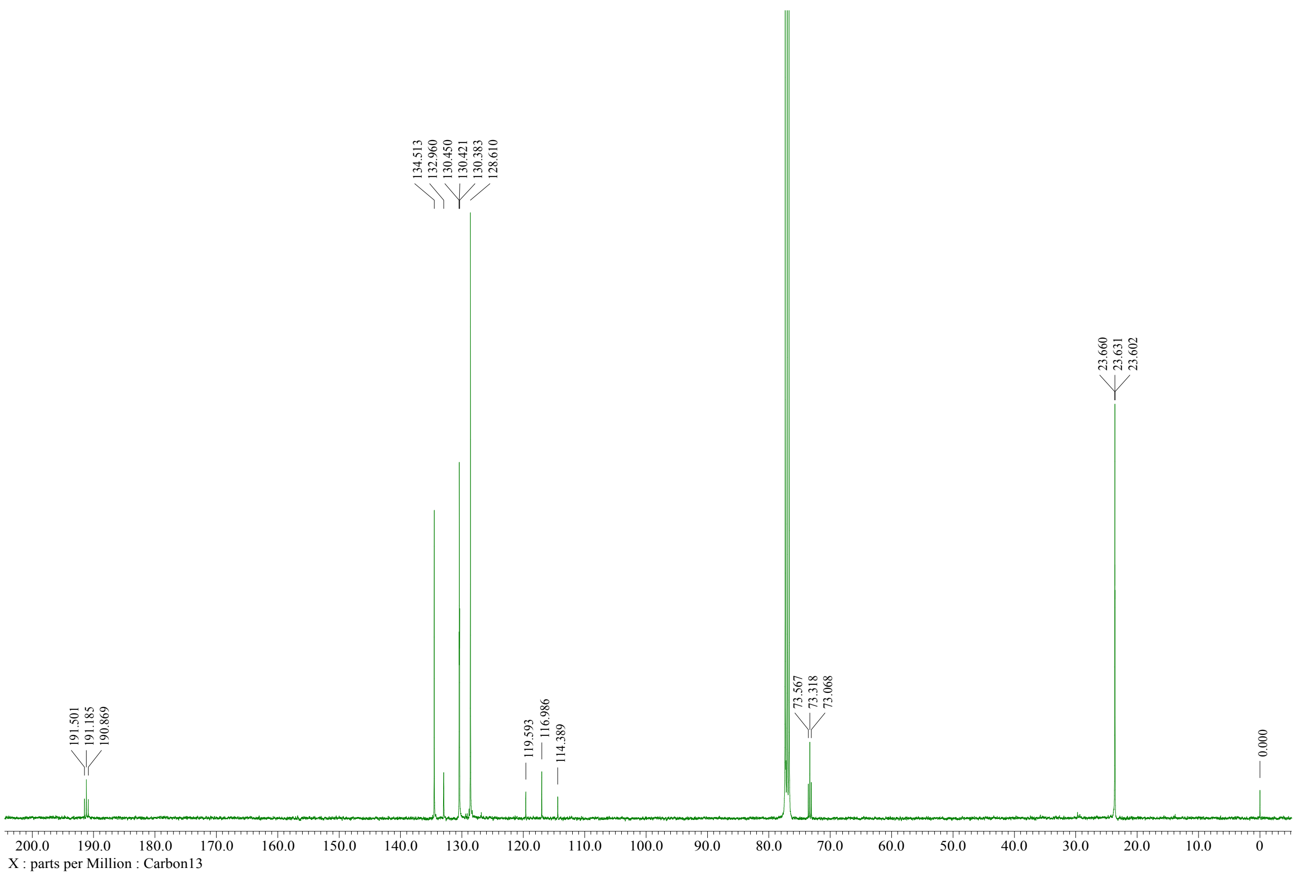
191.501
191.185
190.869
134.513
132.960
130.450
130.421
130.383
128.610
119.593
116.986
114.389
73.567
73.318
73.068
23.660
23.631
23.602
0.000
200.0 190.0 180.0 170.0 160.0 150.0 140.0 130.0 120.0 110.0 100.0 90.0 80.0 70.0 60.0 50.0 40.0 30.0 20.0 10.0 0
X : parts per Million : Carbon13

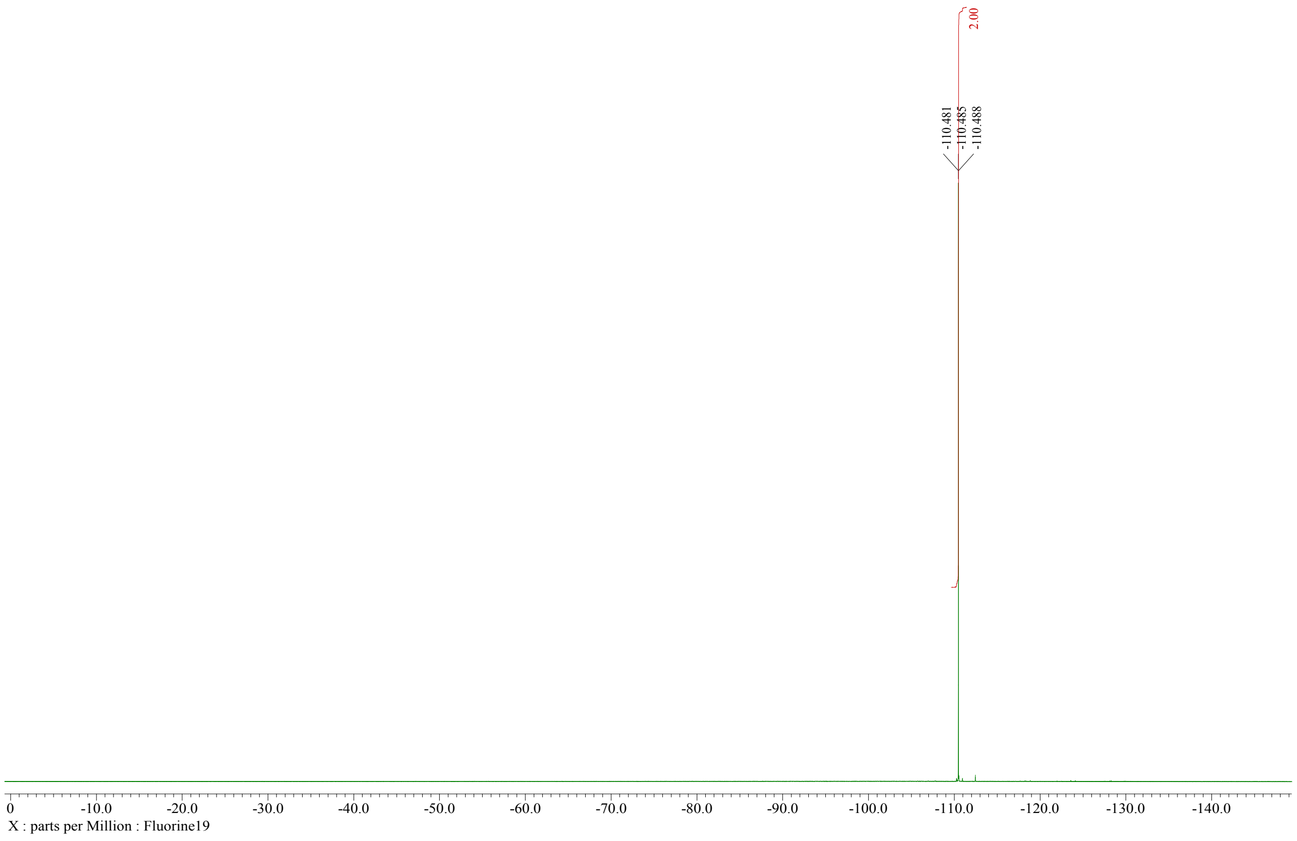
2.00
-110.481
-110.485
-110.488
0 -10.0 -20.0 -30.0 -40.0 -50.0 -60.0 -70.0 -80.0 -90.0 -100.0 -110.0 -120.0 -130.0 -140.0
X : parts per Million : Fluorine19
